# Sex-Biased Transcriptome of *Schistosoma mansoni*: Host-Parasite Interaction, Genetic Determinants and Epigenetic Regulators Are Associated with Sexual Differentiation

**DOI:** 10.1371/journal.pntd.0004930

**Published:** 2016-09-27

**Authors:** Marion A. L. Picard, Jérôme Boissier, David Roquis, Christoph Grunau, Jean-François Allienne, David Duval, Eve Toulza, Nathalie Arancibia, Conor R. Caffrey, Thavy Long, Sabine Nidelet, Marine Rohmer, Céline Cosseau

**Affiliations:** 1 Univ. Perpignan Via Domitia, IHPE UMR 5244, CNRS, IFREMER, Univ. Montpellier, Perpignan, France; 2 Center for Discovery and Innovation in Parasitic Diseases, Skaggs School of Pharmacy and Pharmaceutical Sciences, University of California San Diego, La Jolla, California, United States of America; 3 CNRS, GenomiX IBiSA, Montpellier, France; University of Cambridge, UNITED KINGDOM

## Abstract

**Background:**

Among more than 20,000 species of hermaphroditic trematodes, *Schistosomatidae* are unusual since they have evolved gonochorism. In schistosomes, sex is determined by a female heterogametic system, but phenotypic sexual dimorphism appears only after infection of the vertebrate definitive host. The completion of gonad maturation occurs even later, after pairing. To date, the molecular mechanisms that trigger the sexual differentiation in these species remain unknown, and *in vivo* studies on the developing schistosomulum stages are lacking. To study the molecular basis of sex determination and sexual differentiation in schistosomes, we investigated the whole transcriptome of the human parasite *Schistosoma mansoni* in a stage- and sex-comparative manner.

**Methodology/ Principal Findings:**

We performed a RNA-seq on males and females for five developmental stages: cercariae larvae, three *in vivo* schistosomulum stages and adults. We detected 7,168 genes differentially expressed between sexes in at least one of the developmental stages, and 4,065 of them were functionally annotated. Transcriptome data were completed with H3K27me3 histone modification analysis using ChIP-Seq before (in cercariae) and after (in adults) the phenotypic sexual dimorphism appearance. In this paper we present (i) candidate determinants of the sexual differentiation, (ii) sex-biased players of the interaction with the vertebrate host, and (iii) different dynamic of the H3K27me3 histone mark between sexes as an illustration of sex-biased epigenetic landscapes.

**Conclusions/ Significance:**

Our work presents evidence that sexual differentiation in *S*. *mansoni* is accompanied by distinct male and female transcriptional landscapes of known players of the host-parasite crosstalk, genetic determinants and epigenetic regulators. Our results suggest that such combination could lead to the optimized sexual dimorphism of this parasitic species. As *S*. *mansoni* is pathogenic for humans, this study represents a promising source of therapeutic targets, providing not only data on the parasite development in interaction with its vertebrate host, but also new insights on its reproductive function.

## Author Summary

Parasitic flatworms include more than 20,000 species that are classically hermaphrodites. Among them, the roughly hundred species of *Schistosomatidae* are intriguing because they are gonochoric. Schistosomes are responsible of the second most important parasitic disease worldwide, and eggs are the main cause of the inflammatory symptoms. Thus, studying the sexual reproduction mechanisms of schistosomes is of particular interest for drug development. Schistosome’s sex is genetically determined by the presence of sex chromosomes: ZZ in males or ZW in females. There is, however, no phenotypic dimorphism in the larval stages: sexual dimorphism appears only in the vertebrate host. In order to understand the molecular mechanisms underlying phenotypic sexual dimorphism, we performed a transcriptome analysis (RNA-Seq) in five different stages of the parasite lifecycle as well as a chromatin status analysis (ChIP-Seq) in the non-differentiated stage cercariae and in the adult differentiated stage, for males and females separately. Our work presents evidence that sexual differentiation in *S*. *mansoni* is accompanied by distinct male and female transcriptional landscapes of known players of the host-parasite crosstalk, developmental pathways and epigenetic regulators. Our sex-comparative approach provides therefore new potential therapeutic targets to affect development and sexual reproduction of parasite.

## Introduction

Among the hundred species of *Schistosomatidae*, the *Schistosoma* genus is of particular medical importance as it is pathogenic in humans. Seven *Schistosoma* species are responsible for schistosomiasis (or bilharziasis) [1,2], which represents the second most important parasitic disease after malaria and affects at least 240 million people worldwide [3]. *Schistosoma mansoni*, responsible for the intestinal schistosomiasis, is endemic in Africa and South America [1] and has been a study model for the *Schistosomatidae*. *S*. *mansoni* has a genome of 364.5MB (genome version 5.2), containing 10,852 genes, seven pairs of autosomal chromosomes and one pair of ZZ/ZW sex chromosomes [4,5]. *S*. *mansoni* has a complex life cycle in which a freshwater snail from *Biomphalaria* genus serves as intermediate host and primates or rodent species as definitive host. By definition, sexual reproduction occurs in the vertebrate host. The parasite’s eggs are released in freshwater *via* the feces. Free-living larvae (miracidia) hatch out, and infect the mollusc intermediate host where they transform into sporocysts that release human infecting cercariae after asexual multiplication. These larvae actively seek definitive host skin contact and penetrate the epidermis. During skin penetration, the cercariae lose their tail, and their head undergo drastic morphological and physiological transformations. Within two hours, the free-living larvae become obligatory endoparasitic schistosomula. They leave the dermis to reach the bloodstream and migrate to the liver *via* the lungs [6,7]. At the first steps of the life cycle, some life-history traits have been shown to be different between males and females [8], but no apparent phenotypic sexual dimorphism exists from the eggs to the early stages of schistosomula, in spite of the fact that the parasites possess genetically different sexes. After two to five weeks within the definitive host, and once they reach the hepatic portal system [9], the schistosomula develop from 150 μm juvenile sexually undifferentiated individuals into one centimeter differentiated male or female adult worms. This developmental step could be defined as “sexual differentiation”. Then, dimorphic males and females mate and migrate together to mesenteric venules where they intensely reproduce. Mating is critical for completing gonadal maturation of both sexes [10]. In the adults, the dimorphic phenotype is crucial for the fitness of the parasite: (i) the muscular male clasps the female into his gynaecophoric groove to resist the high blood pressure environment, (ii) the filiform female is able to insinuate into the tiniest venules of the intestine to lay the eggs one by one [11] up to 300 eggs per day. Approximately 50% of these eggs remain trapped in the vertebrate host, causing inflammatory chronic disease [12]. The paired adult parasites can remain for decades in their host, showing their highly adapted interaction with the vertebrate host and the success of the gonochoristic strategy.

In this context, the understanding of developmental and reproductive biology of schistosomes is crucial to fight schistosomiasis. Many studies have addressed the question of the development of the parasite within its vertebrate host and the establishment of the highly efficient reproductive system [13]. Particularly, numerous works have highlighted the responsiveness of the developing *S*. *mansoni* to the host blood microenvironment and shown that the parasite might exploit endocrine and host immune signals to accomplish its development [14–18]. The mating status (*i*.*e*. paired *vs* unpaired) has also been shown to play an essential role for the maturation of both male and female [19–26]. Other molecular studies have highlighted male- or female-biased pathways essential for the development and the reproduction of the parasite [27–35]. Global transcriptomic analyses were carried out on diverse developmental stages [4,36–38] but sex-biased expressions were only explored in adult [38,39] or cercariae [40]. Epigenetic control for gene expression regulation has also been investigated and highlighted sex-specific epigenetic processes with chromatin structural changes occurring on female-specific microsatellite repeats of the W-chromosome during the development of the parasite [41]. Moreover sex-biased and stage-specific microRNA (miRNA) precursor expression suggests that non-coding RNAs (ncRNAs) participate in the *S*. *mansoni* sexual differentiation process [42–44].

In the present report, we propose to correlate molecular mechanisms to phenotypic sexual dimorphism appearance. With this in mind, we present the global transcriptome of *S*. *mansoni* in a stage- and sex-comparative manner. Here, we employed an exhaustive RNA-sequencing analysis in five stages of *in vivo* development of *S*. *mansoni*, from the undifferentiated cercariae to the phenotypically very distinctive male and female worms. The *in vivo* schistosomulum differentiating stages, critical in the host/parasite interaction, were sub-categorized in three morphologically consistent groups of individuals [45–47] and are described here for the first time. In addition to the transcriptomic study, we present male and female genome-wide chromatin structure profiles before (in cercariae) and after (in adults) the somatic sexual differentiation, using ChIP-Seq against H3K27me3 as a proxy.

## Methods

### Sampling and raw data production

#### Animal breeding and ethics statement

Housing, feeding and animal care followed the national ethical standards established in the writ of February 1st, 2013 (NOR: AGRG1238753A). The French Ministère de l’Agriculture et de la Pêche and the French Ministère de l’Education Nationale de la Recherche et de la Technologie provided permit A66040 to the laboratory for animal experiments and certificate to the experimenters (authorization 007083, decree 87–848).

#### Parasite culture and sample preparation

**Fig 1:**
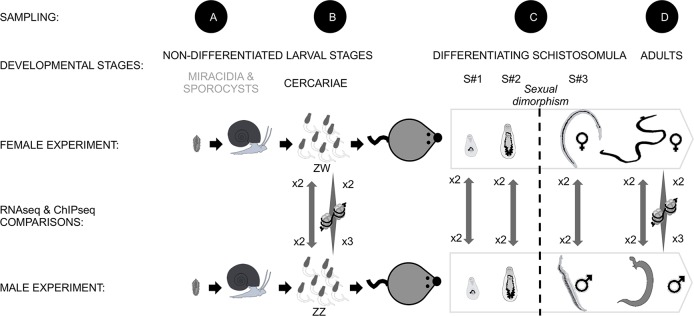
Experimental strategy to compare molecular events occurring through male and female *S*. *mansoni* development. (A) A monomiracidial infection of mollusks was first performed in order to obtain either ZZ male or ZW female clonal cercariae. (B) The sex of the cercarial clones was determined by PCR of female-specific sequences and unisexual infection of mice was then performed. For the molecular study, we recovered one free-living undifferentiated stage of the parasite: cercariae; and four intra-vertebrate stages: (C) the three differentiating schistosomulum stages and (D) the adult stage. The schistosomulum stages are characterized by the onset of sexual dimorphism between s#2 and s#3, and are described more in details in the S1 protocol. For these five stages, we compared the male and female transcriptomes using RNAseq (biological duplicate). For the non-differentiated cercariae and the sexually differentiated adults we also performed a ChIPseq in duplicate for males and triplicate for females.

The human guadeloupean strain of *Schistosoma mansoni* SmGH2 used in this study is maintained in *Biomphalaria glabrata* snail, strain BgGua, and Swiss OF1 mice. In order to obtain unisexual clones of cercariae, monomiracidial infections of mollusks were performed (Fig 1A). Cercariae of each sex were separately recovered 35 days after infection. The sex of the cercarial clones was determined by PCR of female-specific repetitive sequences (S1 Table) [48] and mice were infected with 500 cercariae exclusively male or female (*i*.*e*. unisexual infections) (Fig 1B). *In vivo* schistosomula were obtained by perfusion of the hepatic portal system between three and four weeks post-infection (PI) (Fig 1C) [49]. Schistosomula were sorted into three finely defined stages (s#1, s#2 and s#3, from the younger to the older) in order to have parasite samples as homogeneous as possible. Sorting criteria are based on caecum shape, acetabulum location and gynaecophoric canal appearance in males [45,46]. Briefly, S#1 stage corresponds to the first steps of symmetric development on both sides of acetabulum, the caecum being either non-fused or fused; S#2 stage is a phenotypically asexual stage showing a smaller top part (from the oral sucker to the acetabulum), comparing to the lower part of the body (*i*.*e*. allometric growth); S#3 stage is the first dimorphic developmental stage following gynaecophoric canal apparition in males, and lengthening with loss of pear-shaped aspect in females: the linked caecum is longer than the bifurcated one but it is still less than three-fold longer (S1 protocol). Finally, male and female adult worms were recovered from the unisexually infected mice after 49 days PI by perfusion (Fig 1D). For each stage, parasites were stored at -80°C until RNA extraction.

#### Total RNA isolation

For each sex and stage, experiments were performed in two biological replicates (Fig 1). RNA extractions were performed alternatively from 5,000 cercariae, 800 schistosomula s#1, 400 schistosomula s#2, 200 schistosomula s#3, 20 adult males or 100 adult females. Briefly, parasites were ground in liquid nitrogen and solubilized in TRIzol (Thermo Fisher Scientific). Total RNA was then extracted by adding chloroform. PureLink RNA Mini kit (Ambion) was used for further purification following the manufacturer’s protocol. Total RNA was eluted in 30 μl RNAsecure (Ambion) and incubated at 65°C for 10 min. Samples were then treated with TURBO DNase (TURBO DNA-free, Ambion) and the reaction was stopped by cooling down on ice during two minutes. RNA was finally purified on a column (RNeasy mini kit, QIAGEN) and eluted in 30 μl RNase-free water. Quality and concentration were assessed by spectrophotometry with the Agilent 2100 Bioanalyzer system.

#### Chromatin ImmunoPrecipiation assay

ChIPseq experiments were performed separately on ZZ male (in duplicate) and ZW female (in triplicate) individuals for both phenotypically non-differentiated cercariae and differentiated adult stages (Fig 1). Native immunoprecipitation was done according to Cosseau *et al*. [50] using 4 μl of H3K27me3 antibody (cat. number C15410069, lot number A1821D, 1.45 μg/μl). It required at least 10,000 female or male cercariae, or 20 adult males, or 100 adult females per sample. Further details are available at [51].

#### Illumina library construction and high-throughput sequencing

cDNA library and ChIP library construction and sequencing were performed at the sequencing facilities of Montpellier GenomiX (MGX, France) and GATC Biotech (Germany). Concerning cDNA libraries, the TruSeq stranded mRNA library construction kit (Illumina Inc., USA) was used according to the manufacturer's recommendations on 300 ng of total RNA per condition. Briefly, poly-A RNAs were purified using oligo-d(T) magnetic beads. The poly-A+ RNAs were fragmented and reverse transcribed using random hexamers, Super Script II (Life Technologies, ref. 18064–014) and Actinomycin D. During the second strand generation step, dUTP substituted dTTP to prevent the second strand to be used as a matrix during the final PCR amplification. Double stranded cDNAs were adenylated at their 3' ends before ligation was performed using Illumina's indexed adapters. Ligated cDNAs were amplified following 15 cycles PCR and PCR products were purified using AMPure XP Beads (Beckman Coulter Genomics, ref.A63881). The quantitative and qualitative analyses of the library were carried on Agilent_DNA 1000 chip and qPCR (Applied Biosystems 7500, SYBR Green). The sequencing was performed on a HiSeq2000 in single read 50nt mode. Concerning ChIP libraries, the TruSeq ChIP sample preparation kit (Illumina Inc., USA) was used according to the manufacturer's recommendations on 30 ng of DNA per condition. Briefly, DNAs were blunt ended and adenylated on their 3' ends. Illumina's indexed adapters were ligated to both ends. Ligated DNA were enriched by PCR and sizes separated by electrophoresis. Size selection was performed at 400 base pairs (bp). The quantitative analysis of the DNA library was carried on Agilent High Sensitivity chip and qPCR (Applied Biosystems 7500, SYBR Green). The sequencing was performed on a HiSeq2000 and HiSeq2500 in single read 50nt mode. RNA-Seq and ChIP-Seq reads are available at the NCBI-SRA under the accession numbers SRP071285 (RNAseq on both sexes and ChIPseq on males) and PRJNA236156 (ChIPseq on females).

### Processing of raw data—RNAseq

The bioinformatic pipeline and the quality of the metrics are described in S1 supporting information. All data treatment was carried out under a local galaxy instance [52]. After grooming (*i*.*e*. Fastq sanger format checking) (Fastq galaxy tool v1.0.4, [53]) and quality assessment of the reads (FASTX-Toolkit v0.0.13, [54]) we determined Phred quality scores over 25 for all the nucleotides. Consequently, neither quality filtering nor trimming was applied and all the reads were mapped to the *S*. *mansoni* reference genome (assembly version 5.2) [4] using TopHat (v2.0.9) [55], with the “very sensitive” option for Bowtie2 settings (v2.1.0.0, [56]). The resulting BAM files were converted to SAM format (SAM tools v0.1.18.0, [57]) and the unmapped reads were removed. In order to be consistent in the further differential analyses between the different samples, all datasets were adjusted to the smallest by randomly picking 65,844,021 reads from each file. Exon-intron structure was then reconstructed with Cufflinks (v2.1.1) [58] without any correction parameter: neither quartile normalization, nor bias corrections were applied. All the obtained data were joined with Cuffmerge (v1.0.0) [58] without any genome reference nor guide, in order to create a *S*. *mansoni de novo* reference transcriptome containing the exon-intron structures of the five developmental stages. The GTF file of this transcriptome is available at the IHPE laboratory webpage [59] and the sequences of all the assembled unique transcripts (TCONS) in the S5 Table (sheet1). We quantified each sample read abundance by mapping each condition to this *de novo* reference transcriptome with HTseq (v0.6.1p1) with the overlap resolution mode union [60]. Finally, the differential gene expression levels between sexes were analyzed with the DESeq package (v1.12.1) [61]. We carried out five comparisons: male *vs* female cercariae, male *vs* female schistosomula s#1, male *vs* female schistosomula s#2, male *vs* female schistosomula s#3, male *vs* female adult worms (Fig 1). Considering one stage and one transcript, significant difference in expression between sexes was evaluated according to the adjusted *P*-value (Padj) for multiple testing with the Benjamini-Hochberg procedure which controls false discovery rate (FDR). Genes that were significantly (Padj <0.05) overexpressed in one sex compared to the opposite sex, were defined as “sex-biased genes”.

### Functional annotation

The *de novo* assembled transcriptome was entirely and automatically annotated (S5, sheet2). Blastx searches against the non-redundant database of the NCBI (14-oct-2013) were performed on a local server using BLAST 2.2.26+ version [62]. XML files were loaded onto Blast2GO for gene ontologies (GO), mapping and annotation with version b2g_sep13 of the Blast2GO database [63]. InterProScan 5–44.0 version [64] was then used and Interproscan GO were merged to Blast2GO. Using BLAT (v34) [65] we aligned the *de novo* transcriptome to the *S*. *mansoni* coding sequences of the reference genome v5.2 (ASM23792v2.30) got at the Ensemble Genomes resource [66], setting the minimum score to 50. In the case of alignment of a Cufflinks gene (XLOC_ID) to multiple reference genes (Smp_IDs), the hit with the higher identity was considered as the correct alignment. Reciprocally, only the higher coverage for a reference transcript was conserved. We then use Cuffcompare, an associate utility program of Cufflinks (v2.2.1) in order to characterize the type of matches between the Cufflinks transcripts and the reference transcripts (v5.2). Functional analysis introduced in this paper mainly consider the three stages cercariae, schistosomula s#2 and adults because these three stages display the highest number of significant sex-biased genes, due to the quality of the biological replicates [61]. Concerning the two other schistosomulum stages s#1 and s#3, we detected less than 100 significant sex-biased genes (Padj<0.05). Thus, only the 100 best adjusted *P*-values were analyzed (S2 Table) and heatmaps revealed the consistence of replicates in these stages (S1 supporting information, slide5).

#### Gene Ontology sex-comparative enrichment

To identify GO terms that were significantly up- or down-regulated between males and females, a Blast2GO enrichment analysis was performed (version 2.6.4) (S3 Table) [63]. Six test sets were used, corresponding to the sex-biased transcripts of three stages: cercariae, schistosomula s#2 and adults. Increases in GO terms were considered statistically significant at *P* < 0.02 (Fisher exact test). We focused our analysis on biological processes only. The number of GO terms in each category was normalized towards the total number of enriched GO terms for each dataset.

#### Functional clusters of sex-biased genes

For each gene (XLOC), we considered only the annotation of the longest unique transcript (TCONS) for manually sorting into 16 functional categories based on their sequence homology (S4 Table). In these 16 functional categories, we manually picked sex-biased transcripts depending on the function of their orthologs and sorted them into six functional representative under-categories related to: “homeotic genes”, “growth-factor pathways”, “steroid pathway”, “mobile genetic elements”, “splicing” and “chromatin modifications”. The transcripts of each category were then clustered according to the developmental pattern of their sex-biased expression (represented by the log10 fold-change) using Gene Cluster 3.0 [67] software with the complete linkage method and hierarchical parameters. Graphic representations were obtained with Java treeView software 1.1.6r4 version [68].

#### Functional *de novo* annotation of the 100 best sex-biased genes for each stage

For the five developmental stages, the top 100 of the sex-biased genes (100 best Padj) were *de novo* manually and separately annotated using Blastx (v2.2.30) [62], CD-search (for blast for conserved domain) (http://blast.ncbi.nlm.nih.gov/Blast.cgi, [69]) and the information available from the web based interface geneDB (http://www.genedb.org, [70]). This functional *de novo* annotation is presented in S2 Table.

#### Identification of miRNA precursors

In order to identify transcribed miRNA precursors, the *de novo* transcriptome was compared with miRBase sequences [71] using Blast. Only the transcripts covering the stem loop sequence of known *S*. *mansoni* miRNA precursors with 100% of homology were conserved (S5 Table, sheet4).

### Quantitative real-time Polymerase Chain Reaction (RT-qPCR)

For each developmental stage and sex of the parasite, first strand cDNA synthesis and qPCR validation experiments were achieved on two different biological replicates. 500ng of the purified total RNA were reverse transcribed using identical concentration (250 nM) of random and oligo-dT primers of Maxima H Minus Reverse Transcriptase kit (ThermoSCIENTIFIC). qPCRs were performed using a LightCycler 480 System (Roche Diagnostics) with the LightCycler 480 SYBR Green I Master Mix (Roche Diagnostics). Single product amplification was checked by analysis of the amplicon melting curve and capillary migration on a Labchip GX DNA assay system (PerkinElmer). For each reaction, the crossing point (Cp) was determined using the second derivative maximum method using Light Cycler Software version 3.3 (Roche Diagnostics). For each studied stage, sex, and replicate, the level of transcription was normalized using the mean geometric transcription rate of three reference sequences *Smp_093230* (*Sm-arp 10*, *actin protein 10*), *Smp_197220* (*Sm-RPL35*, *subunit of the oligosaccharyltransferase*) and *Smp_089880* (*Sm-fad oxidoreductase*, *FAD dependent oxidoreductase domain containing protein*) previously described [72]. The stability indexes of those reference genes were calculated using NormFinder (v20) [73] to assess if they were stable (i) during all the developmental stages and (ii) between males and females. Forward and reverse primers (Eurogentec) were designed for 43 genes (in addition to the three housekeeping genes) with the Primer3plus web based interface [74], the lack of putative primer dimer was checked with Perlprimer (v1.1.21) and the uniqueness of the target was verified using blast on the *S*. *mansoni* genome v5.2. Primer efficiencies were >1.8 for each couple. Primer sequences and expected PCR product sizes are listed in S1 Table. Correlation between RNAseq and qPCR was tested both globally and individually for each gene with a Spearman Rank test (S2 supporting information).

### Processing of raw data—ChIPseq

#### ChIP-Seq data analysis

After grooming (*i*.*e*. fastq sanger format checking) (Fastq galaxy tool v1.0.4, [53]) and quality assessment of the reads (FASTX-Toolkit v0.0.13, [54]) we determined Phred quality scores over 25 for all the nucleotides. Consequently, neither quality filtering nor trimming was applied and all the reads were mapped to the *S*. *mansoni* reference genome (assembly version 5.2) [4] using TopHat (v2.0.9) [55], with the “very sensitive” option for Bowtie2 settings (v2.1.0.0, [56]). Mapping quality in Bowtie 2 is related to “uniqueness” of the mapping [56]. SAM alignment files were converted into the bed format with pyicos [75] and sorted with sortBed -i of the bedtools suite [76]. For peaks identification an equal number of 15,000,000 random lines in the bed-file was chosen for each biological replicate. Identification of peaks was done with ranger of Peakranger v1.16 [77] with P-value cut off 0.0001, FDR cut off 0.01, Read extension length 200, Smoothing bandwidth 99 and Delta 1. We used the input samples (*i*.*e*. from unbound samples) as negative controls for the peakcalling (-c). The quality of the metrics is presented in S1 supporting information.

#### Comparative EpiChIP analysis

Average histone modification profiles around transcriptional start site (TSS) were generated in a 6,000 bp window from -1,000 to +5,000 bp relative to the TSS of genes, using EpiChIP v0.9.7-e [78]. As input, we used the 15,000,000 randomly sampled aligned reads that also served as inputs for PeakRanger [77]. The average histone profiles were generated on the first chromosome and the linkage group ZW. For this purpose, we used the *de novo* transcriptome (GTF output file of Cuffmerge) and selected the 6,225 and the 5,797 expressed genes of these chromosomes respectively. The average H3K27me3 and input profiles were generated for the two male biological replicates and the three female biological replicates. Each average H3K27me3 profile was normalized with its respective input average profile. The distribution of chromatin structural changes from the transcription starting site were compared according to the stage and the sex of the parasite using Kolmogorov-Smirnov two sample tests (S1 supporting information, slide10).

### List of ID numbers for genes mentioned in the text

Protein coding genes mentioned in the text are accessible on GeneDB ([70], http://www.genedb.org/) under the following accession numbers: Smp_093230: actin protein 10 (Sm-arp10) / Smp_197220: subunit of the oligosaccharyltransferase (Sm-RLP35) / Smp_089880: FAD dependent oxidoreductase domain containing protein (Sm-fad oxidoreductase) / Smp_196410: dachshund / Smp_000530: zinc finger transcription factor gli2 / Smp_141030: EGF receptor kinase substrate 8-like / Smp_035260: EGF receptor kinase substrate 8-like / Smp_134550: Neuropeptide (Sma-npp-27) / Smp_212730: tyrosine kinase, TK group, Src family (SmTK3) / Smp_174880: FOG / Smp_103470: protein mago nashi / Smp_045950: transformer 2 protein / Smp_009600: serine: threonine protein kinase PLK1 (polo-like kinase 1) / Smp_159800: MEG-2 (ESP15) family / Smp_159810: MEG-2 (ESP15) family / Smp_010550: MEG 15 / Smp_163630: MEG-4 (10.3) family / Smp_033600: Dicer 2 / Smp_118190: staphylococcal nuclease domain-containing protein / Smp_165220: polycomb protein EED / Smp_006250: polycomb protein Scm1 (Sex comb on midleg homolog, Scm1).

miRNA precursors mentioned in the text are accessible on miRBase ([71,145], http://www.mirbase.org/index.shtml) under the following accession numbers: MI0027256: sma-mir-1a / MI0027255: sma-mir-8458 / MI0027196: sma-mir-125c / MI0027285: sma-mir-8483 / MI0027247: sma-mir-8451 / MI0027258: sma-mir-8459 / MI0027222: sma-mir-8429.

## Results

### Identification of “sex-biased genes”

We generated the transcriptomes of cercariae, three developmental stages of schistosomula (s#1, s#2 and s#3) and adult worms from unisexual infection. Details and illustrations for the sorting criteria of the three classes of schistosomula are shown in S1 protocol. For each developmental stage, males and females were analyzed separately with two biological replicates (Fig 1).

In total, the transcriptome sequencing of these 20 samples yielded 1,080,386,261 Illumina single reads of 50bp and 981,363,482 mapped to the *S*. *mansoni* reference genome (v5.2). These mapped reads could be assembled in 54,956 unique transcripts identified as “TCONS”, (S5 Table, sheet1) representing putative splice variants of 34,755 genes identified as “XLOC” (S5 Table, sheet1). Among these expressed genes, 9,581 annotated genes of the *S*. *mansoni* reference genome (v5.2) could be identified with the blat aligner (S5 Table, sheet3). Notably, we found that 6.95% of the transcripts could correspond to novel isoforms of known transcripts and that 45.39% were located in intergenic region of the reference genome (S1 supporting information, slide6).

**Fig 2:**
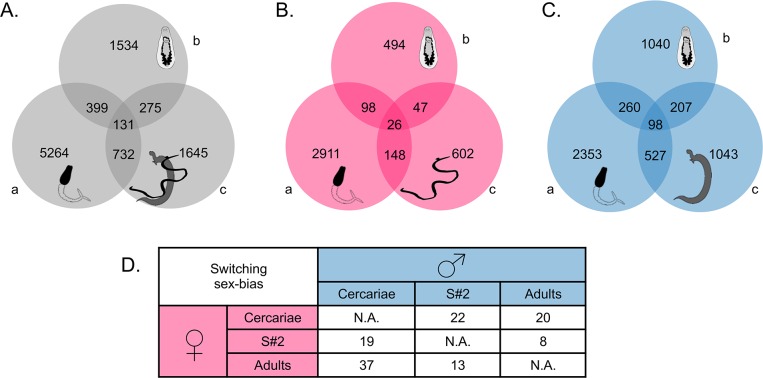
Venn diagrams of sex-biased genes. For each of the three developmental stages (a) cercariae, (b) schistosomula s#2 and (c) adults, significant differences (Padj<0.05) between sexes were detected by DEseq analyses. Here are represented the number of sex-biased genes by stage and those that are shared between different stages: (A) when pooling male- and female-biased genes, or considering only (B) female-biased genes or (C) male-biased genes. (D) The table represents the number of sex-switching biased genes that are more expressed alternatively in females or in males depending on the developmental stage.

Quantification of read abundance and DEseq analysis of differential gene expression between sexes were performed for each stage. We present here significant transcriptomic differences (adjusted *P*-value < 0.05) observed in cercariae, schistosomula s#2 and adults. These three stages taken together, 7,168 genes appeared as significantly differentially expressed between sexes (*i*.*e*. either more expressed in males or more expressed in females) in at least one of the classes (Fig 2A). They were defined as “sex-biased genes” and represent 20.62% of the total number of expressed genes. For cercariae, schistosomula s#2 and adults, we identified respectively 5,264 (2,353 male-biased and 2,911 female-biased); 1,534 (1,040 male-biased and 494 female-biased) and 1,645 (1,043 male-biased and 602 female-biased) sex-biased genes (Fig 2). Ninety-eight male-biased and 26 female-biased genes were consistent through all the stages (Fig 2). Interestingly, several genes are alternatively more expressed in females or in males, they were qualified as “sex-switching biased genes” (Fig 2D). To further support our transcriptomic analysis, qPCR experiments were performed to validate the expression patterns obtained from the DEseq analysis. Forty-three transcripts were randomly tested and a Spearman Rank test determined that RNAseq and qPCR experiments were significantly (p<0.0001) and positively correlated, with a coefficient R = 0.65 (S2 supporting information).

### Gene Ontology analysis: Disparity in perception of environmental factors, metabolism and chromatin structure between sexes

**Fig 3:**
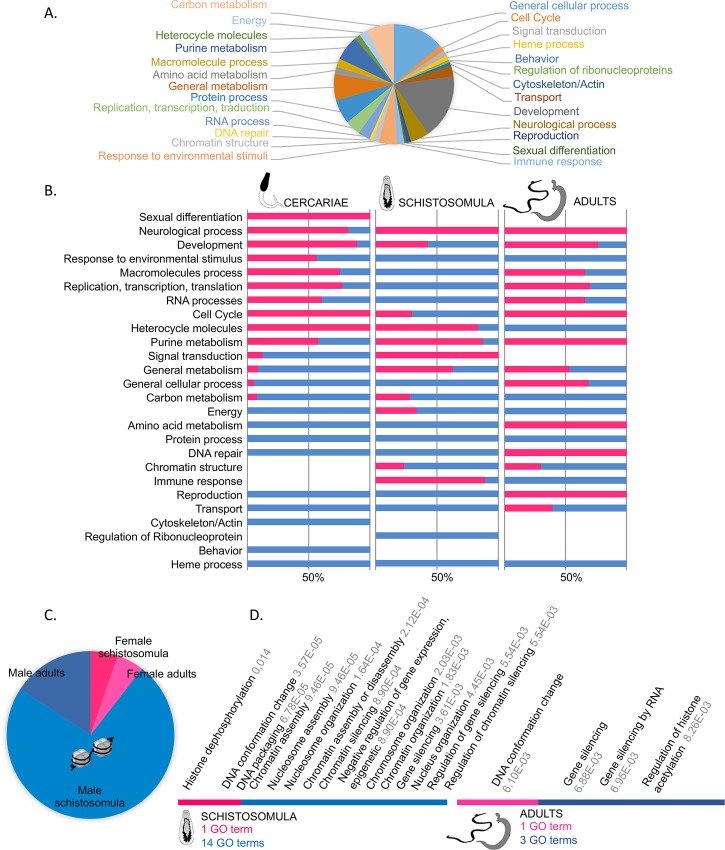
GO term analysis of sex-biased genes. (A) Distribution of total sex-biased GO categories (*i*.*e*. both sexes and considering the three stages). (B) Percentage of male (in blue) and female (in pink) sex-biased GO terms for each category within each stage: cercariae, schistosomula s#2 and adults. (C) Percentage of sex-biased GO terms related to chromatin structure in schistosomula and adults. (D) Sex-biased GO terms related to chromatin structure in schistosomula and adults.

Among the 7,168 “sex-biased” genes, 2,468 corresponded to known genes (S4 Table, sheet20) and 1,598 other could be *de novo* functionally annotated by the blast2GO analysis (S4 Table, Sheet19), totalizing 4,065 functionally annotated sex-biased genes. To gain insight into major biological processes that would be enriched during development in either male or female individuals, we performed an exact Fisher test using the blast2GO software, focusing on the “biological processes” ontology. Enriched GO terms are presented in the S3 Table. To obtain a more synthetic overview, we manually sorted the GO terms into more general categories and considered the GO term enrichment for each of them (Fig 3A). Of particular interest in the context of host-parasite interaction, we detected that the response to “environmental stimulus” occurred in cercariae at the same level for both males and females (Fig 3B). Nonetheless, GO terms were different and indicated a distinct perception of environmental factors (S3 Table). Female cercariae displayed a better “response to light” and “mechanical stimulus” than males. Male cercariae seemed better responsive to “chemical stimuli” and this capacity was maintained in schistosomula and adult worms. Among the GO terms involved in response to “chemical stimuli”, we noticed the particular biological processes “response to vitamin D” and “growth epidermal factor” (both enriched in male cercariae, S3 Table sheet1), and “response to cortico/gluco-steroid stimulus” (enriched in male schistosomula, see S3 Table sheet3). Along with these differences in environment perception we detected distinct representation of terms related to metabolic functions between sexes. Particularly, some categories were more enriched in males whatever the developmental stage such as “protein process”, “heme related process”, “energy” and “carbon metabolism” (Fig 3B). Besides, and of special interest for gene expression regulation, we detected a particular enrichment of the category “chromatin structure” in male schistosomula and adults. This category did not display any differences between sexes in the larval stage (Fig 3C). The corresponding GO terms are represented in the Fig 3D. They all converged toward a strong chromatin re-organization during the vertebrate stages of the parasite, emphasized in males. Finally, considering processes putatively involved in the sexual dimorphism appearance, our GO term analysis indicated that the very general “development” category was over-represented in female cercariae and adults, but more in male schistosomula (Fig 3B). More specifically, “sexual differentiation” was a category over-represented in female cercariae while the “reproduction” category was over-represented in male cercariae and schistosomula and then reversely over-represented in adult females (Fig 3B).

### Functional analysis of sex-biased genes: Focus on development and survival in a host/parasite context

The 4,065 functionally annotated sex-biased genes were manually classified into 16 classes related to (i) the general developmental pathways, (ii) the sex determination and/or sexual differentiation pathways and (iii) the detection of the environment and/or the interaction with the vertebrate host (S4 Table).

#### Distinct general developmental pathways are involved in males and females

**Fig 4:**
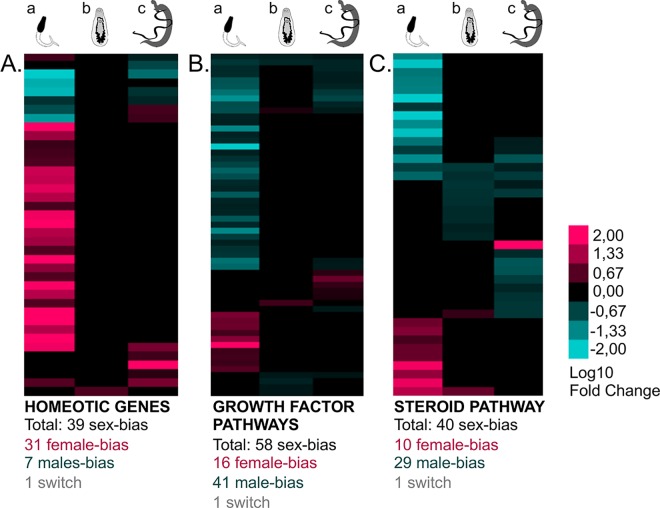
General clustering of sex-biased gene expression depending on their “general developmental pathway” functional category. Male-biases are represented in blue, female-biases in pink and non-significant differences (Padj>0.05) in black. Three stages are considered: (a) cercariae, (b) schistosomula s#2 and (c) adults. (A) 39 homeotic genes are mainly female-biased in cercariae. (B) 58 genes linked to growth factor pathways are mainly male-biased in cercariae and adults. (C) 40 genes are related with the steroid pathway and are mainly male-biased through the three stages. The functional annotation and details on gene expression are provided in S6 Table.

In female cercariae, the over-representation of the “development” GO category was related to a high extent in female-biased homeotic genes representing 32 of the 39 sex-biased homeotic genes (Fig 4A). Furthermore, we detected a female-bias for another interesting developmental gene: “dachshund homolog” (Smp_196410), which is also differentially regulated between sexes in *Drosophila melanogaster* [79]. We also observed the sex-switching bias of expression of a transcription factor, “*gli2*” (*Smp_000530*), which was male-biased in cercariae and adult stages but female-biased in schistosomula s#2. Besides, six growth factor pathways showed mainly male-biased gene expression: the epidermal growth factor (EGF) pathway, the fibroblast growth factor pathway, the tumor necrosis factor pathway, the shared pathway of the transforming growth factor beta and the bone morphogenetic protein, the wnt signaling pathway and the notch signaling pathway. We identified 41 male-biased genes for 58 sex-biased members for signal molecules, receptors, downstream effectors and/or regulators (Fig 4B). Interestingly, three gene products matched with “*EGF Receptor kinase substrate 8-like*” (*Smp_141030*, *Smp_035260* and the non-referenced XLOC_034752). These receptors were previously described as expressed in schistosome gonads [32]. Consistently with the GO term analysis which underlined a “response to cortico/gluco-steroid stimulus” particularly enriched in male, we also detected that the steroid pathway was strongly male-biased from cercariae to adult stages (29 of the 40 sex-biased genes). Among them we identified enzymes, receptors and products involved in cholesterol perception and transport, or lipid metabolism (Fig 4C). In relation to the neuro/hormonal system and of particular interest, we detected a male-biased neuropeptide among the male-biased 100 best *P*-values in adults (*Smp_134550*, or *Sma-npp-27* [80]). In conclusion, these results pointed out a major female-biased pattern of expression of homeogenes at the cercarial stage, together with a male-biased profile of growth factor pathways. It further highlighted that males and females differently expressed genes related to hormonal and nervous systems.

#### Sex determination and sexual differentiation pathway analysis revealed some key candidates

We identified several interesting male-biased genes in cercariae and schistosomula. In cercariae, we pointed out the male-biased “*cytoplasmic kinase SmTK3*” (*Smp_212730*) which was shown to be involved in schistosome reproduction [81], and the Z-specific gene *“fog*” (*Smp_174880*, [82–84]). In schistosomula, the unplaced “*mago nashi protein homolog*” (*Smp_103470*, [85–87]) and the pseudoautosomal gene “*transformer-2*” (*Smp_045950*, [88,89]) were of particular interest because of their involvement in sexual differentiation in model organisms. Finally, in adults, we detected the female-biased expression of the “*polo-like kinase 1*” (*Smp_009600*), known to be involved in schistosome reproduction [30]. To conclude, we highlighted here some key genes expressed in a sex-biased manner and whose function are related to the sexual identity (*i*.*e*. sex determination or sexual differentiation pathways) and the reproductive function (*i*.*e*. meiosis) either in schistosome or other model organisms.

#### Environment detection and interaction with the vertebrate host involved different gene repertories in males and females

Because of their parasitic mode of development, and taking into account the differences in “response to environmental stimulus” shown by the GO analysis, we assessed if males and females interacted in the same way with their vertebrate host, not only in term of nutritive micro-environment, but also in term of host invasion (*i*.*e*. penetration and/or immunogenic aspects) (S4 Table, sheet5). Thus, we identified sex-biased genes that encode functions related to host invasion: males and females displayed different repertories of proteases (96 sex-biased proteases) and especially metalloproteases. Each sex presented also distinct repertories of protease inhibitors, delivered proteins and tegumental antigens or receptors. We detected for instance 63 sex-biased tegumental proteins, and nine were identified as “*tetraspanin*”, which are known to be involved in the induction of protective immunity [90,91]. In addition, we particularly detected sex-biased expression of “*venom-allergen-like*” (*VAL*) molecules and “*microexons genes*” (*MEGs*) which are supposed to be involved in the molecular interaction with the host [92–96]. Four biased *MEGs* and one *VAL* were among the 100 best *P*-values. Four *MEGs* were previously identified and annotated in the reference genome (*Smp_159800*, *Smp_159810*, *Smp_010550* and *Smp_163630*) [4]. Interestingly, we identified two novel *MEGs* (XLOC_030935 and XLOC_006117), showing both a majority of very small exons (<37b), mainly symmetrical (with a number of bases divisible by 3) as defined classically [93]. Their structures are presented in the S7 Table. In conclusion, we identified sex-biased expression of known or supposed players of the crosstalk between the parasite and its vertebrate host as well as previously non-annotated *MEGs* with a sex-biased expression profile.

### Sex-biased candidate regulators of gene expression

Players of transcription mechanisms, genetic mobile elements, chromatin remodeling and other DNA modifications together with RNA processing were examined by a combination of the transcriptomic approach and epigenetic analysis.

#### Post-transcriptional regulatory mechanisms are themselves sex-biased

**Fig 5:**
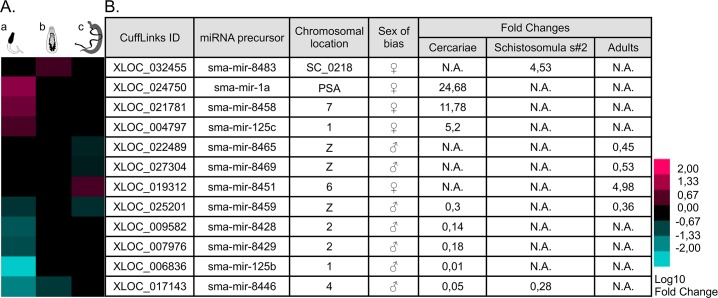
**Overview of the 12 sex-biased miRNAs through three stages of the schistosome lifecycle (a: cercariae, b: schistosomula s#2, c: adults).** (A) General clustering of sex-biased miRNA expression: seven male-biased miRNAs were detected (in blue), five were female-biased (in pink). (B) Table of sex-biased miRNA precursors IDs, chromosomal location and expression bias. Four miRNAs were located on sex chromosomes: three on the Z-specific regions (as defined by Protasio *et al*. [4]) were overexpressed in males. *Key*: *PSA = PseudoAutosomalRegion*, *Z = Z-specific gene*, *N*.*A*. *= no sex-bias of expression*.

**Fig 6:**
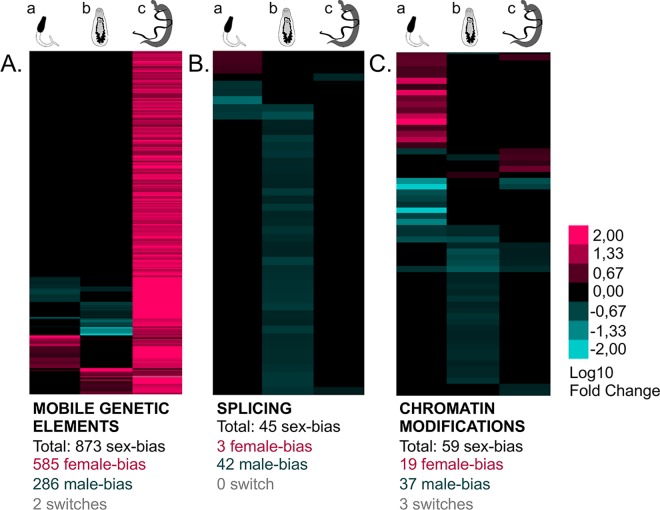
General clustering of sex-biased gene expression depending on their “regulation of gene expression” functional category. male-biaises are represented in blue, female-biaises in pink and non-significant differences (Padj>0.05) in black. Three stages are considered: (a) cercariae, (b) schistosomula s#2 and (c) adults. (A) 873 mobile genetic elements were detected as sex-biased, mainly in female adult worms. (B) 45 splicing-linked genes are mainly male-biased in schistosomula s#2. (C) 59 genes involved in chromatin modification are mainly male-biased in schistosomula s#2. The functional annotation and details on gene expression are provided in S6 Table.

Two male-biased candidates of the “*RNA-induced silencing (RISC) complex”* were detected: the endoribonuclease “*dicer2”* (*Smp_033600*) in cercariae, and the “*staphylococcal nuclease domain-containing protein 1*” (*Smp_118190*) in adults. This *RISC* complex is a key player of the processing of single stranded RNAs, such as miRNAs precursors, involved in gene silencing at the post-transcriptomic level [97 for review]. The presence of miRNAs precursors was also investigated by intersecting the sex-biased transcripts with the known miRNA precursors in *miRBase*. Twelve perfect matches with known *S*. *mansoni* miRNA hairpin precursors were identified [43,98]: seven were male-biased and five female-biased (Fig 5). Interestingly, four of them were located on the sex chromosomes. Concurrently to this approach, we detected 873 genetic mobile elements that were mainly associated to female adults (Fig 6A).

Besides these non-coding elements, we detected that 42 genes among the 45 “splicing machinery and factors” were male biased (Fig 6B), together with other genes involved in post-splicing control and mRNA editing (S4 Table, sheet6). In conclusion, these results showed that sex-biased post-transcriptional regulation could occur to differentially regulate gene expression between males and females.

#### Chromatin modifications: The histone mark H3K27me3 dynamic is different between males and females

**Fig 7:**
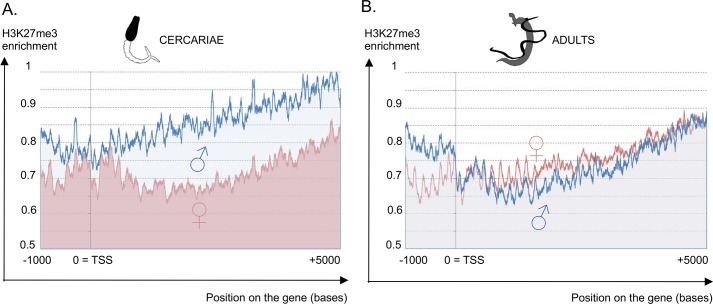
Average H3K27me3 enrichment profile of the chromosome 1 genes. X axis represents the position in bases relative to the transcriptional start site (TSS, position 0), Y axis represents the normalized mean enrichment of reads obtained after a ChIP targeting the H3K27me3 mark on (A) cercariae and (B) adults. The EpiChIP enrichment has been calculated around the TSS of the chromosome 1 transcripts obtained in our RNA-Seq experiment. It has been normalized with the same mean enrichment of reads obtained after a ChIP without antibody. The mean profile for two male biological replicates (blue) and three female biological replicates (red) are represented. The profile for each replicate is provided in S1 supporting information (Slide 8). The same profile was obtained for the ZW linkage group.

Thirty-seven genes among the 59 candidates to “chromatin structure regulation” were male-biased (Fig 6C). Particularly, we detected among the 100 best adjusted *P-*values in schistosomula s#2, the Z-specific “polycomb protein EED” (Smp_165220) and the Z-specific gene “*sex comb on midleg homolog*” (*Scm1*, *Smp_006250*) [99,100]. To verify the hypothesis of a strong genome-wide chromatin reorganization during the vertebrate stage, we performed a ChIP-Seq analysis against H3K27me3. This histone mark was chosen because it is known to be a major player for the regulation of developmental genes in embryonic stem cells [101]. Furthermore, the tri-methylation of H3K27 plays also a key regulatory role in *Schistosoma* development as it was earlier identified during cercaria to schistosomulum transition [102]. We generated an average enrichment profile for H3K27me3 on cercariae and adults, in distinct samples of both sexes (Fig 1). This analysis was performed on the first chromosome, as representing the autosomes (*i*.*e*. largest placed chromosome of 79.6 Mb, [4]) and on the sex-chromosomes, independently on Z-specific and pseudo-autosomal regions (as defined by [4]). The same average enrichment profile for H3K27me3 was obtained on the first chromosome and on the sex-chromosomes both in the Z-specific and pseudo-autosomal regions (S1 supporting information, slides 8 to 10). In males, the tri-methylation of H3K27 was clearly removed from cercariae to adults (56.5% of maximum difference with the Kolmogorov-Smirnov two sample tests) whereas in females the dynamics of this histone modification is less than twice smaller (25.6% of maximum difference). The H3K27me3 enrichment profile in cercariae differs between the two sexes both upstream and along the transcription unit (Fig 7A); whereas in the adult stage, males and females display the same profile after the transcriptional start site (TSS), while their profile upstream the TSS remains different (Fig 7B). Therefore, our results showed a sex-biased dynamics of the H3K27 tri-methylation, with an emphasized depletion of this histone mark along the transcription unit in males, during the vertebrate stage in both autosomes and sex-chromosomes.

## Discussion

We provide here the first comparative analysis of male and female transcriptomes of *S*. *mansoni* through three developmental stages: the undifferentiated larval cercariae, an *in vivo* schistosomulum stage s#2, and the dimorphic adult worms. Two other *in vivo* schistosomulum stages s#1 and s#3 were sequenced but non-detailed here (see DEseq results in S5 Table sheet5, and 100 best *P*-values in S2 Table). Along with the described schistosomulum stage s#2, they were never reported before. Our functional analysis highlighted three important aspects of the parasite biology that differ between sexes: (i) Distinct general developmental pathways are involved between male and female schistosome development together with more specific sex determination/differentiating candidates. (ii) Male and female parasites interact in distinct ways with their vertebrate host. (iii) Male and female display different landscapes of pre- and post-transcriptional mechanisms of gene expression regulation, associated with different dynamic of the H3K27me3 histone mark.

### Sex determination/differentiation candidates and male/female differences in general developmental pathways

**Fig 8:**
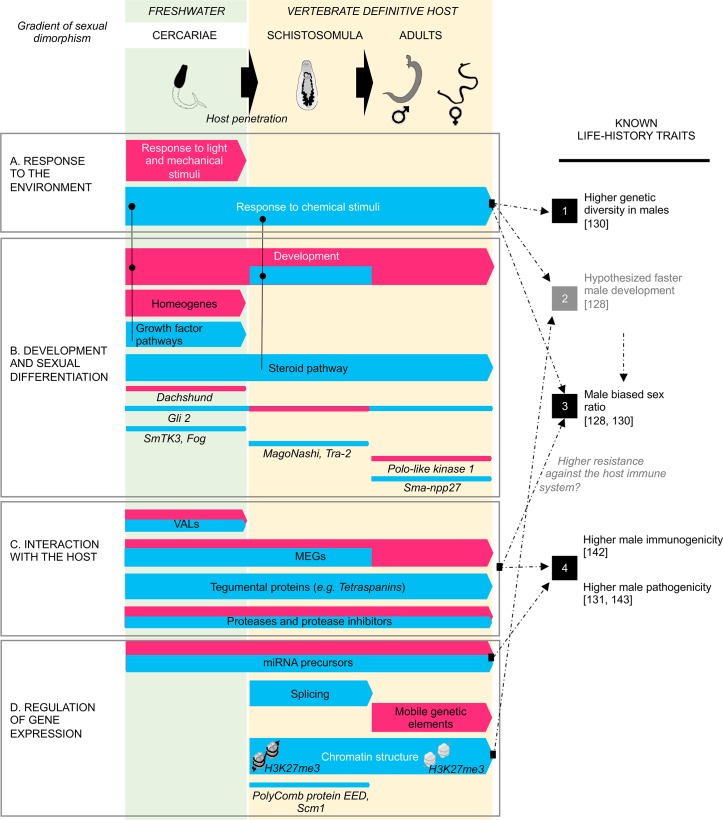
Male-biased (in blue) and female-biased (in pink) players accompanying *Schistosoma mansoni* sexual differentiation, and hypothetical links with known life-history traits (dotted line). (A) The cercarial stage is marked by gene expression differences in environment detection: the male-response to more specific chemical stimuli can facilitate the host detection and thus could allow a larger dispersion of the male cercariae explaining explain the higher genetic diversity observed later in male adults (1) [130]; and (ii) be responsible of the male-biased sex ratio (3) [130]. (B) Molecular events preparing parasite development start before the host penetration: while intrinsic pathways (homeogenes) are female-biased, growth factor and hormonal pathways are male-biased. These pathways could not only be intrinsic to the parasite, but also linked to the host microenvironment. These differences in developmental strategy could lead to the previously hypothesized faster male development (2) [128] and thus indirectly to the male-biased sex ratio (3) [130]. Several important candidates to sex determination and/or differentiation were identified both in males and females through the three stages of development. (C) Different putative players of the host/parasite interaction were detected as sex-biased trough the different stages of development. These differences could lead to both higher male immunogenicity and pathogenicity (4) [131, 142, 143]. Furthermore, if they facilitate male-resistance against the host immune system, they could indirectly be responsible of the male-biased sex-ratio (3) [130]. (D) Different putative regulators of gene expression were detected as sex-biased. Particularly, the depletion in H3K27me3 histone mark could lead to an optimized male development (2). *Lines and dots link subcategories to more general biological process*. *“Black” life-history traits were previously published and “grey” ones are hypothesized*. *Abbreviations*: *VALs = Venom Allergen Like proteins*, *MEGs = Micro Exon Genes*

Despite the large attention given to *S*. *mansoni* reproduction biology, only few molecular candidates have been shown to be key players of sex determination and differentiation and most are related to sexual maturation after pairing. They mainly belong to the “kinome” [27,29–32,103,104], particularly the TGFβ pathway [17,25,105,106], and were identified as new potential target for therapies [107]. Our transcriptional study allows the identification of several promising candidates with a sex-biased expression (Fig 8B), and we discuss here four of them that, in our opinion, need further attention. The “*mago nashi protein homolog*” (*Smp_103470*) is male-biased in schistosomula. Its gene product is a nuclear factor highly conserved from plants to animals [85,108] and is a sex-determined protein in some model organisms. For instance, it is necessary for oocyte organization in *Drosophila melanogaster* [108,109] and for maintaining oogenesis in *Caenorhabditis elegans* hermaphrodite individuals [110]. Moreover, previous experiment performed in *S*. *japonicum* showed its importance for male teste organization [87]. However, no data was provided for females. Another important candidate is the female-biased “*dachshund”* gene (*Smp_196410*) which by contrast is involved in male development of *D*. *melanogaster* [79]. It could be an example of downstream actor of the sex-differentiation cascade. The zinc finger transcription factor “*gli2*” could also be a candidate of particular interest because it is related to the TGFβ pathway intensely studied for its involvement in sexual differentiation of *S*. *mansoni* [17,25,105,106]. Moreover, “*gli2*” is alternatively male or female biased depending on the parasite developmental stage and thus confirms its “bipotentiality” [111] which could play a pivotal role in “sex-orientation” of the development. For instance, in the sexual differentiation context it could switch to shape the sperm/oocyte decision. This kind of mechanism is known to be involved in germ-line cell decision in hermaphroditic nematodes [112]. In our study, several sex-switching genes appear alternatively overexpressed in males or females depending on the developmental stage. We for instance hypothesize that these genes could be particularly sensitive to host environment, and molecular dysfunction on these candidates or environment variation could consequently lead to abnormal hermaphroditism occasionally observed in *S*. *mansoni* [113,114]. Besides, we describe here for the first time the male-biased expression of the neuropeptide *Sma-npp-27* (*Smp_134550*) in adults. In acoelomate, like schistosomes, the nervous system occupies a particular role: as they lack both body cavity and circulatory system, the nervous system is not only involved in sensory and neuromuscular signalization, but can also transmit developmental and hormonal signals [115]. Accordingly, the study of Collins *et al*. [80] defined a role for peptide hormones in controlling reproductive physiology, particularly in males, in the planaria *Schmidtea mediterranea*. Our results could fit into their hypothesis of shared molecular mechanisms of reproduction among flatworms, independently of their hermaphroditic or gonochoric status.

During the cercarial stage, homeogenes are more expressed in females while growth factor and hormonal pathways are over expressed in males successively in cercariae and vertebrate stages (Fig 8B). The obligate implication of the host system regarding the parasite development has received much attention [15,116–124] because interfering with the perception of the host environment might be a possible therapeutic target [107,122]. Previous studies showed that the physiological and reproductive status of the worm is strongly influenced by the host [125] and hermaphroditic-like structures can occasionally appear under the environmental pressure of this host [113]. In this context, growth factor and hormonal pathway over-expression in males could not only result from intrinsic developmental pathways but also could reflect a better receptivity to the host micro-environment (Fig 8A).

### Sex-biased host-parasite crosstalk, and putative links to life history traits

Cercariae actively seek vertebrate skin contact and prepare host invasion [126,127]. We have identified here female biased genes involved in response to light and mechanical stimuli in cercariae while males biased genes encodes function involved in response to chemical stimuli (Fig 8A). We hypothesize that males may be consequently more efficient to encounter their host, which is consistent with the male-biased sex ratio in adults [128,129] (Fig 8.3). Furthermore, it could account for the higher genetic diversity in male adult worms [129,130] (Fig 8.1), *via* a better capacity of dispersion. After penetrating the vertebrate host, parasites have to overcome its immune system. In this context, we detected sex-biased expression of secreted and/or tegumental molecules known or supposed to be involved in host invasion (Fig 8C) among which, known and new micro-exon genes encoding for variant secreted proteins [92], proteases and protease inhibitors [131,132], *“tetraspanins”* [90]. In addition, we show for the first time sex-biased expression of *“venom-allergen like proteins”* [92,133]. We thus further hypothesize that the sex-biased expression of these candidates could explain the known differences between male and female immunogenicity and pathogenicity (Fig 8.4) [134].

### Sex-biased gene expression regulation: Which could be the candidates?

Our analysis highlights different putative mechanisms of gene expression regulation between males and females: miRNA (microRNA) precursors, mobile genetic elements, genes encoding proteins involved in chromatin reorganization, and post-transcriptional events (splicing, mRNA editing) (Fig 8D). Previous studies have highlighted miRNAs stage- and sex-biased expression [42–44,98] as important factors for female maturation [44,135]. In addition, other non-coding RNAs (*e*.*i*. W-specific long non-coding RNAs) are involved in sex chromosome evolution [41]. Besides, schistosome miRNAs have been detected in the blood of the definitive host that underlines their possible implication in the pathological processes of schistosomiasis [136,137]. Although our experiment was not fully designed to identify mature miRNAs, we found 12 transcripts encoding miRNA hairpin precursors with a sex-bias expression in favor of either male or female individuals. The sex-biased pattern of their expression may be another argument to explain the differences of pathogenicity between males and females (Fig 8.4). Our result suggest that, non-coding RNAs certainly deserve fully dedicated experiments for both their implication in the sexual biology of schistosomes and their role in the interaction with the human host. Another interesting molecular mechanism highlighted in our study is the sex-biased expression of mobile genetic elements. This aspect undoubtedly needs further investigation because: (i) of their high bias of expression (great proportion among the 100 best *P*-values (S2 Table)), (ii) of their particular structural features (intronic localization of genes), and (iii) previous works in model organisms showing that they could be essential in the reproduction by playing a role in sex chromosome inactivation [138] and in the dosage compensation mechanism [139]. Another interesting aspect of schistosome developmental biology is the intense chromatin remodeling that occurs after penetration into the definitive host [102]. We show here that the male schistosomulum development is characterized by an over-expression of genes involved in nucleosome assembly, structural maintenance of chromatin and structural genes encoding for histones compared to female's development. These transcriptomic observations are reinforced by a depletion of the H3K27me3 histone mark, emphasized in males, from cercariae to adult. H3K27me3 is known to be widely involved in developmental gene silencing from invertebrates to vertebrates [101,102,140]. We hypothesize that the observed accentuated depletion of this histone mark in males could be a strategy for enhancing male development (Fig 8D and 8D.2).

Finally all our observations seem to converge to an optimized male survival and development in a host-parasite context. Considering the particular sexual biology of *S*. *mansoni* [141] this strategy could serve to an enhanced pre-zygotic paternal investment and thus benefit not only the males but also to the females which need males to achieve their maturation. This ability is essential for the survival and thus the reproductive success of the couple.

### Concluding remarks and perspectives

In our study we present sex-biased pathways, related to development and host-parasite interaction, which accompany sexual differentiation in *S*. *mansoni*. We further propose putative gene determinants of sex determination/differentiation in this organism and candidate epigenetic mechanisms involved in its regulation. Our analysis represents a first step towards the identification of sex-pivotal genes and now further studies are required to validate the candidate functions and to clarify sexual differentiation pathways. These coming works could involve tissue-specific expression, knock-down and/or over-expression of the candidate genes. In a larger context, we observed an important proportion of newly transcribed regions: further works leading to their deep characterization could participate to the improvement of the actual reference genome v5.2.

Since schistosomiasis represents the second most important parasitic disease worldwide and affects millions of people, providing new therapeutic targets is a substantial issue for the scientific community [144]. We describe here a new *in vivo* transcriptome of free-living as well as parasitic developmental stages of both sexes of *S*. *mansoni*. Our work paves a new way toward understanding the complex molecular interplay that occurs between the host and *S*. *mansoni* through the sexual differentiation of the parasite, thereby suggesting new potential drug targets and vaccine candidates.

## Supporting Information

S1 Protocol Sorting criteria of *in vivo* schistosomulum stages. (PPTX)

S1 Supporting information Bioinformatic workflow and quality of the metrics. (PPTX)

S2 Supporting information RT-qPCR experiments. (PPTX)

S1 Table PCR/qPCR primers. (XLSX)

S2 Table *De novo* functional annotation and structural analysis of the 100 best adjusted *P*-values. (XLSX)

S3 Table Gene Ontology. (XLSX)

S4 Table Functional analysis. (XLSX)

S5 Table Global data: Sequences of unique transcripts (TCONS), functional annotation of the *de novo* transcriptome, cross-references between XLOC and Smp_ID, Blast results for miRNA precursors and DEseq data by stage. (XLSX)

S6 Table Gene annotation and expression levels of clustered genes (Corresponding to Fig 3). (XLSX)

S7 Table Micro-exon gene structures. (XLSX)
