# Supplementary material for: Sex-Biased Transcriptome of Schistosoma mansoni: Host-Parasite Interaction, Genetic Determinants and Epigenetic Regulators Are Associated with Sexual Differentiation
Source: PLoS Negl Trop Dis. 2016 Sep 27;10(9):e0004930. doi: 10.1371/journal.pntd.0004930 (PMC5038963; doi:10.1371/journal.pntd.0004930)
Supplement: S1 Protocol — (PPTX) [file pntd.0004930.s001.pptx]

## Slide 1
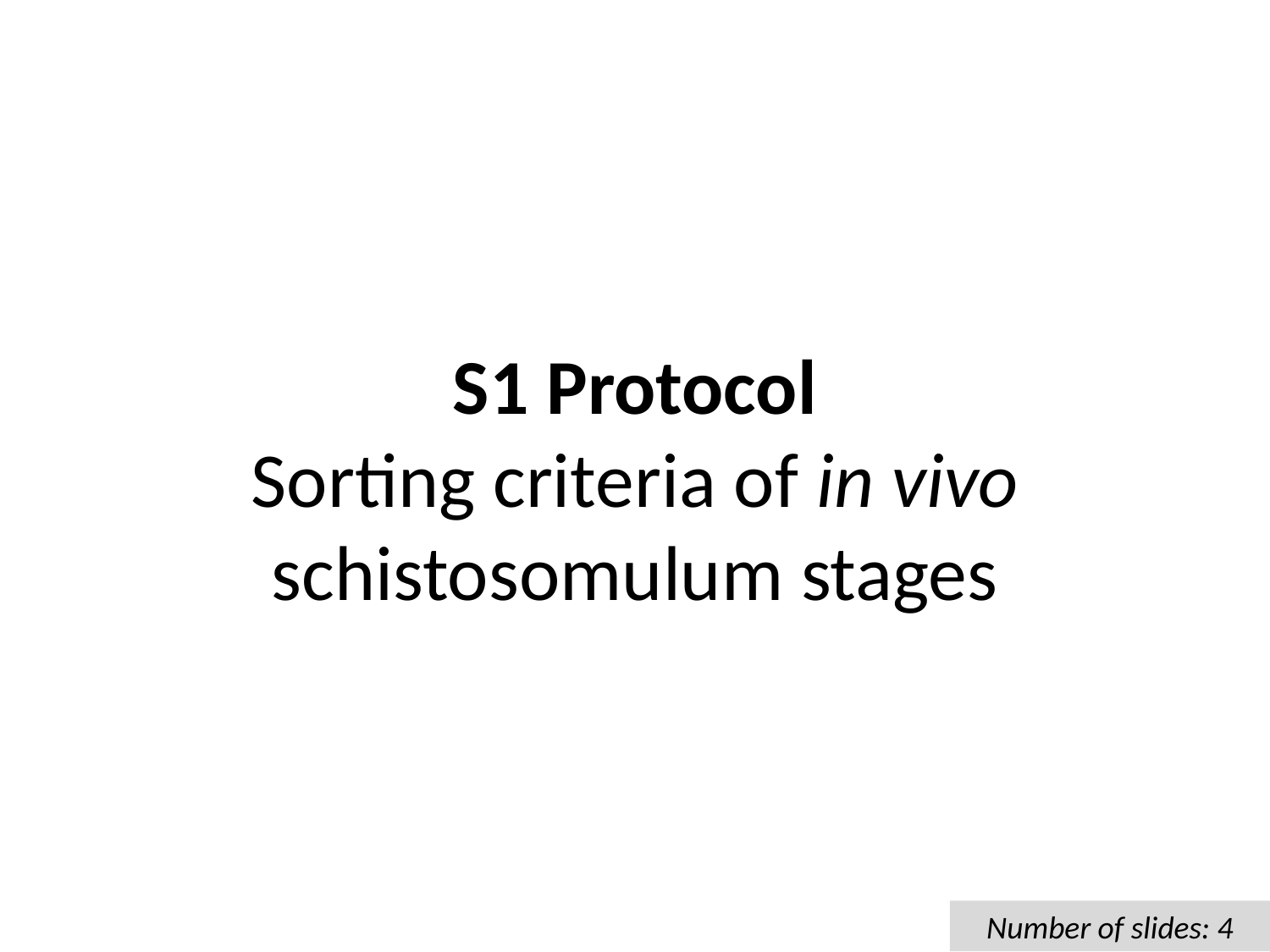

# S1 Protocol Sorting criteria of in vivo schistosomulum stages
Number of slides: 4

## Slide 2
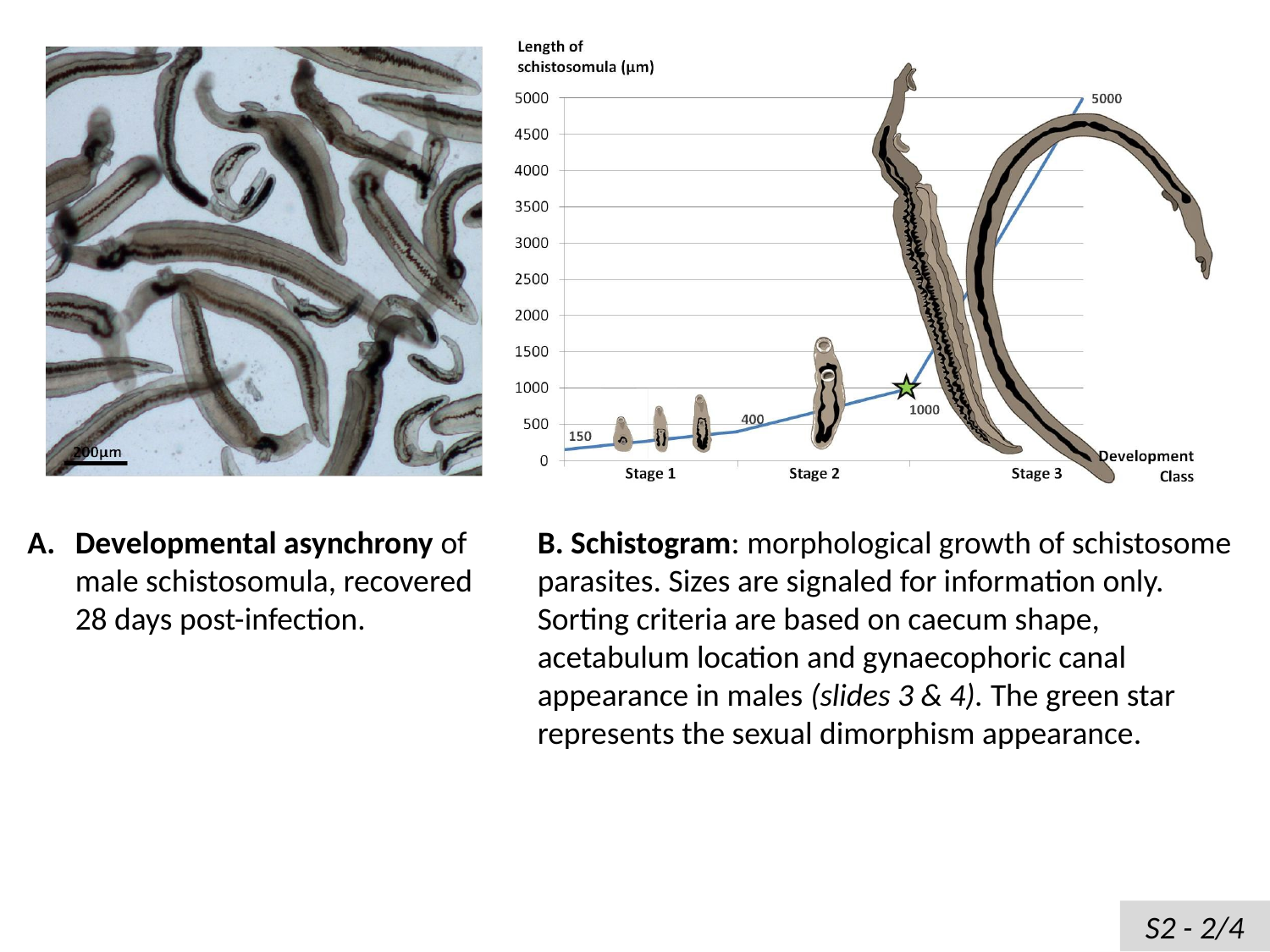

B. Schistogram: morphological growth of schistosome parasites. Sizes are signaled for information only. Sorting criteria are based on caecum shape, acetabulum location and gynaecophoric canal appearance in males (slides 3 & 4). The green star represents the sexual dimorphism appearance.
Developmental asynchrony of male schistosomula, recovered 28 days post-infection.
S2 - 2/4

## Slide 3
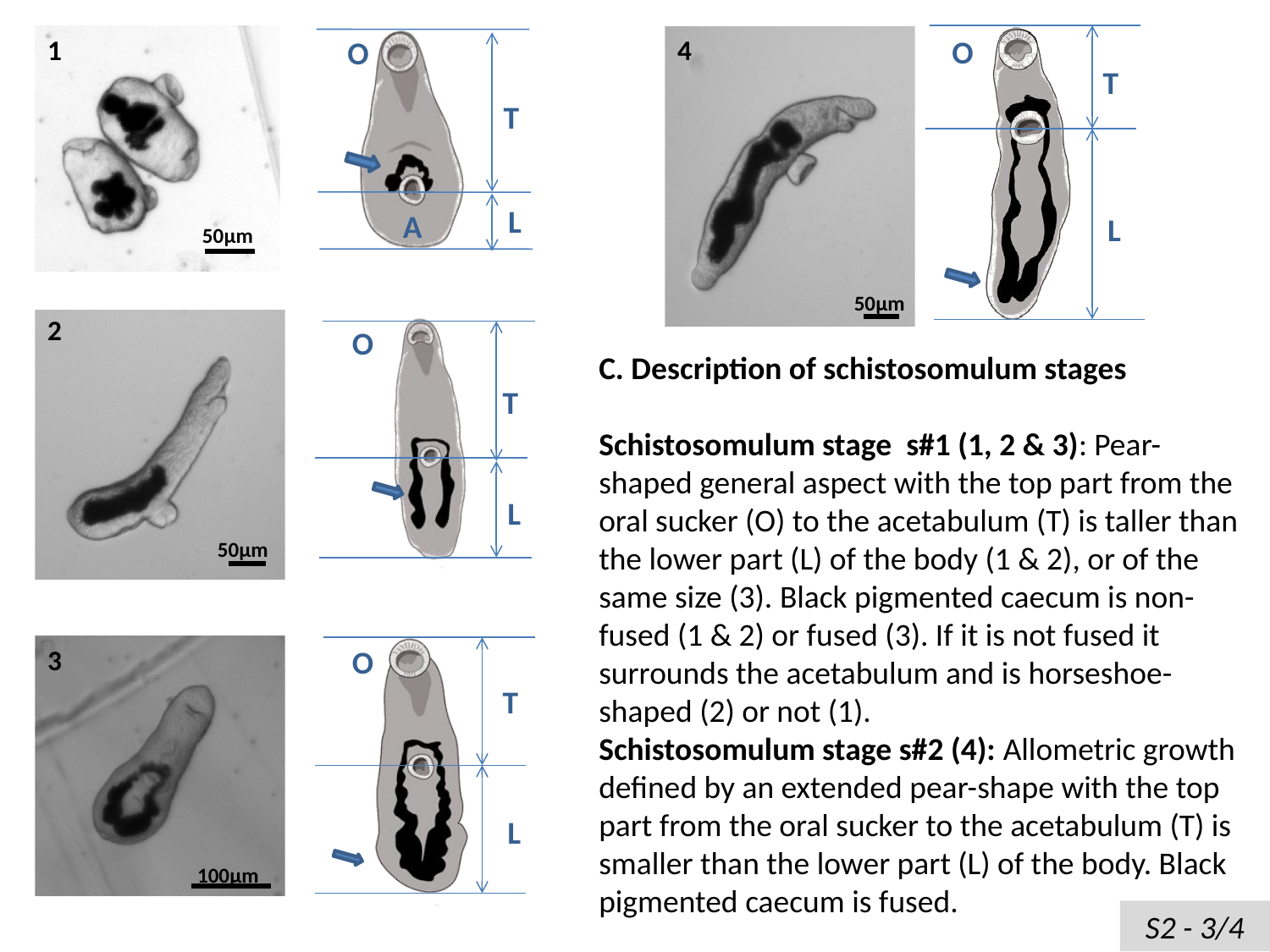

O
T
L
A
50μm
50μm
1
4
O
T
L
50μm
2
O
C. Description of schistosomulum stages
Schistosomulum stage s#1 (1, 2 & 3): Pear-shaped general aspect with the top part from the oral sucker (O) to the acetabulum (T) is taller than the lower part (L) of the body (1 & 2), or of the same size (3). Black pigmented caecum is non-fused (1 & 2) or fused (3). If it is not fused it surrounds the acetabulum and is horseshoe-shaped (2) or not (1).
Schistosomulum stage s#2 (4): Allometric growth defined by an extended pear-shape with the top part from the oral sucker to the acetabulum (T) is smaller than the lower part (L) of the body. Black pigmented caecum is fused.
T
L
100μm
3
O
T
L
S2 - 3/4

## Slide 4
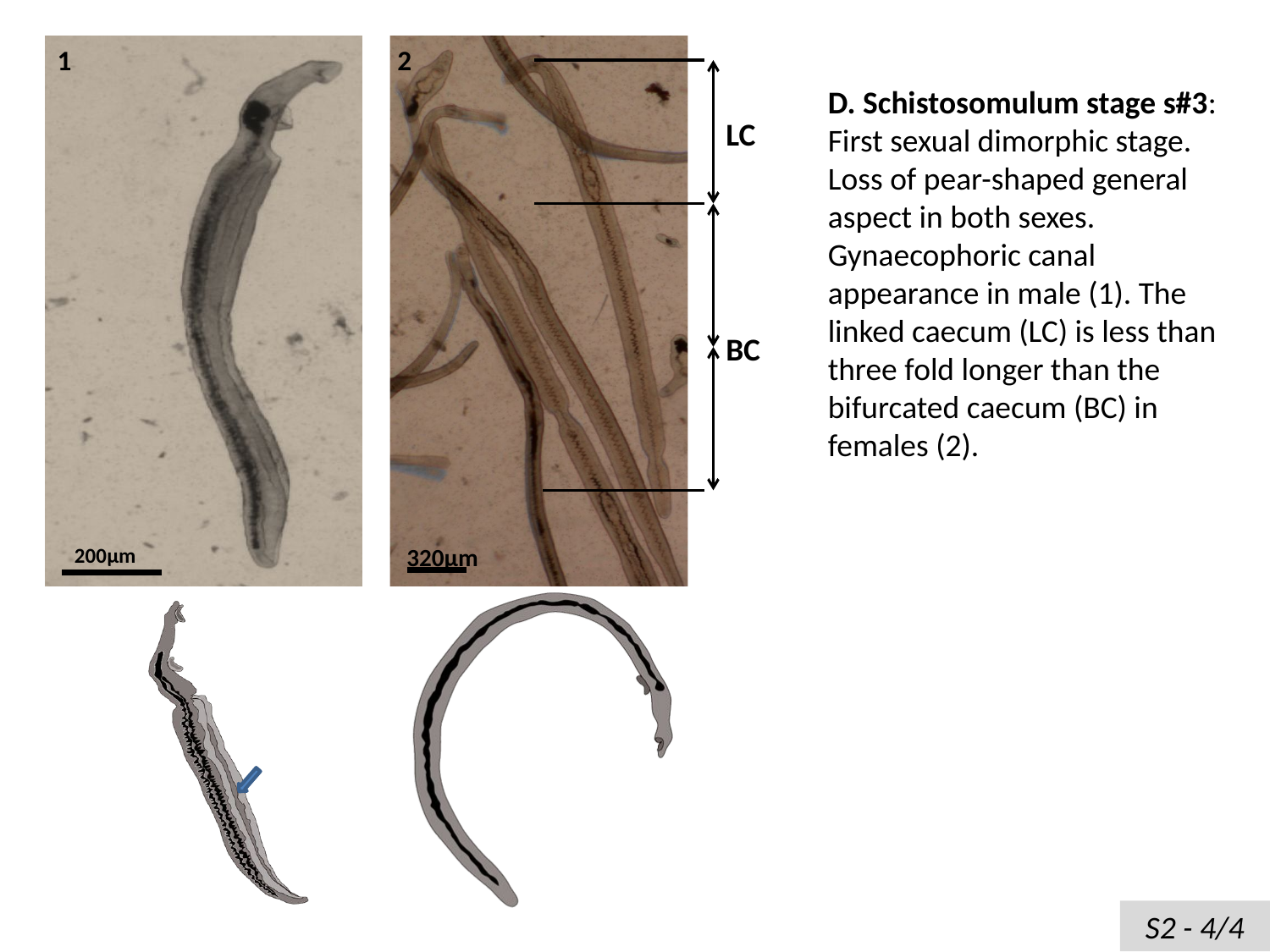

200μm
1
320µm
LC
BC
2
D. Schistosomulum stage s#3: First sexual dimorphic stage. Loss of pear-shaped general aspect in both sexes. Gynaecophoric canal appearance in male (1). The linked caecum (LC) is less than three fold longer than the bifurcated caecum (BC) in females (2).
S2 - 4/4
