# Supplementary material for: Sex-Biased Transcriptome of Schistosoma mansoni: Host-Parasite Interaction, Genetic Determinants and Epigenetic Regulators Are Associated with Sexual Differentiation
Source: PLoS Negl Trop Dis. 2016 Sep 27;10(9):e0004930. doi: 10.1371/journal.pntd.0004930 (PMC5038963; doi:10.1371/journal.pntd.0004930)
Supplement: S2 Supporting information — (PPTX) [file pntd.0004930.s003.pptx]

## Slide 1
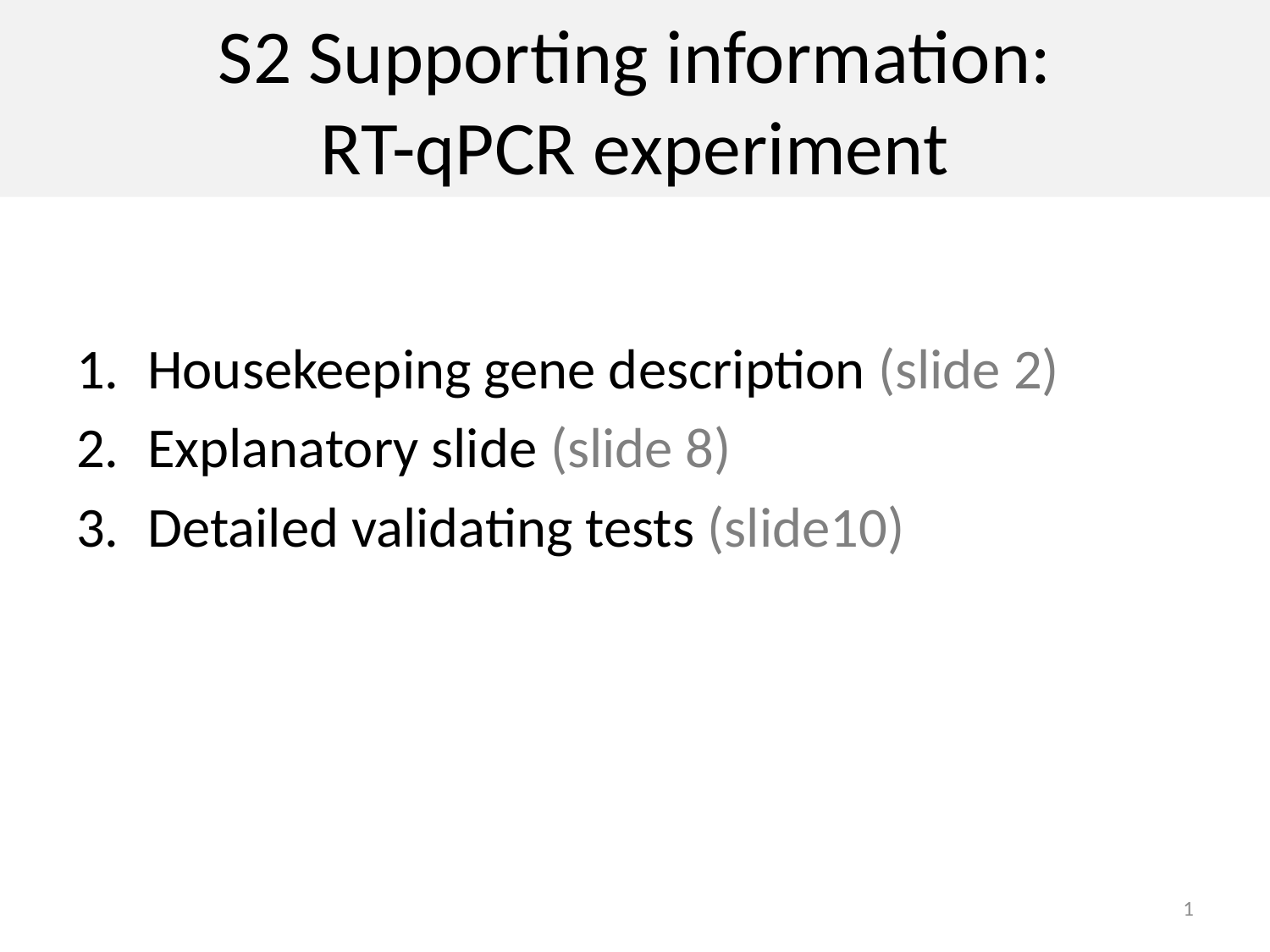

# S2 Supporting information: RT-qPCR experiment
Housekeeping gene description (slide 2)
Explanatory slide (slide 8)
Detailed validating tests (slide10)
1

## Slide 2
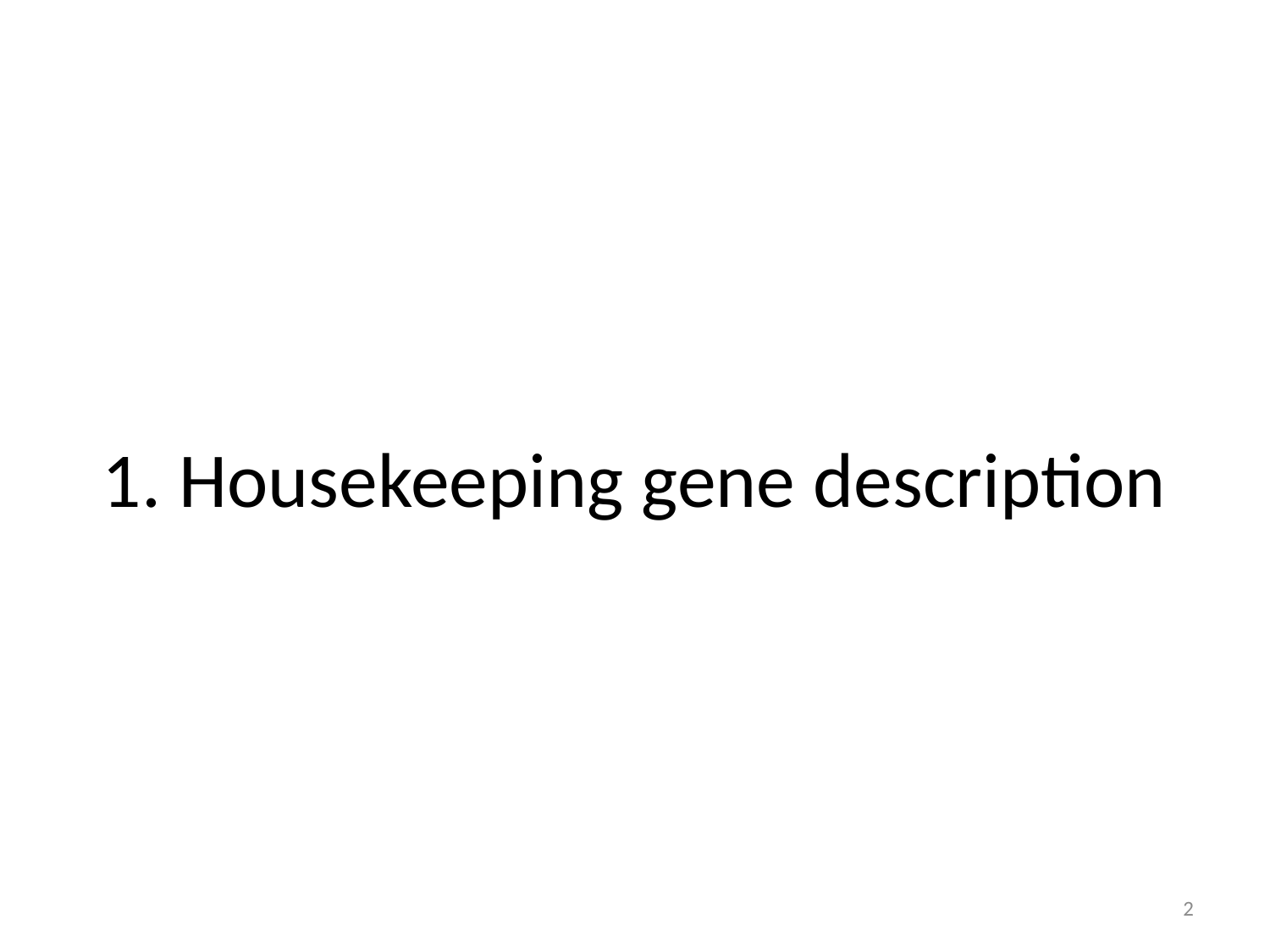

# 1. Housekeeping gene description
2

## Slide 3
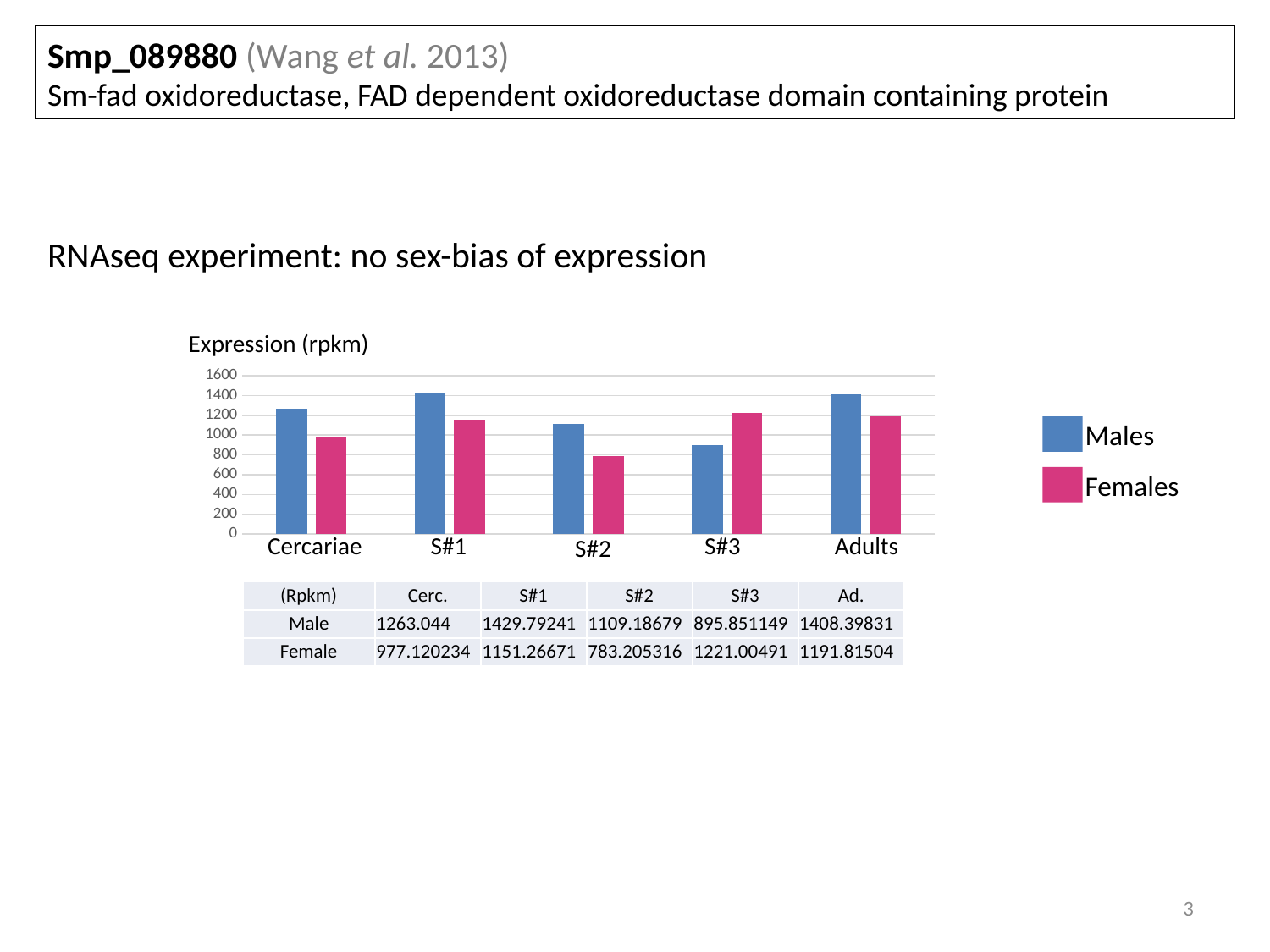

Smp_089880 (Wang et al. 2013)
Sm-fad oxidoreductase, FAD dependent oxidoreductase domain containing protein
RNAseq experiment: no sex-bias of expression
Expression (rpkm)
### Chart
| Category | | |
|---|---|---|Males
Females
Cercariae
S#1
S#3
Adults
S#2
| (Rpkm) | Cerc. | S#1 | S#2 | S#3 | Ad. |
| --- | --- | --- | --- | --- | --- |
| Male | 1263.044 | 1429.79241 | 1109.18679 | 895.851149 | 1408.39831 |
| Female | 977.120234 | 1151.26671 | 783.205316 | 1221.00491 | 1191.81504 |
3

## Slide 4
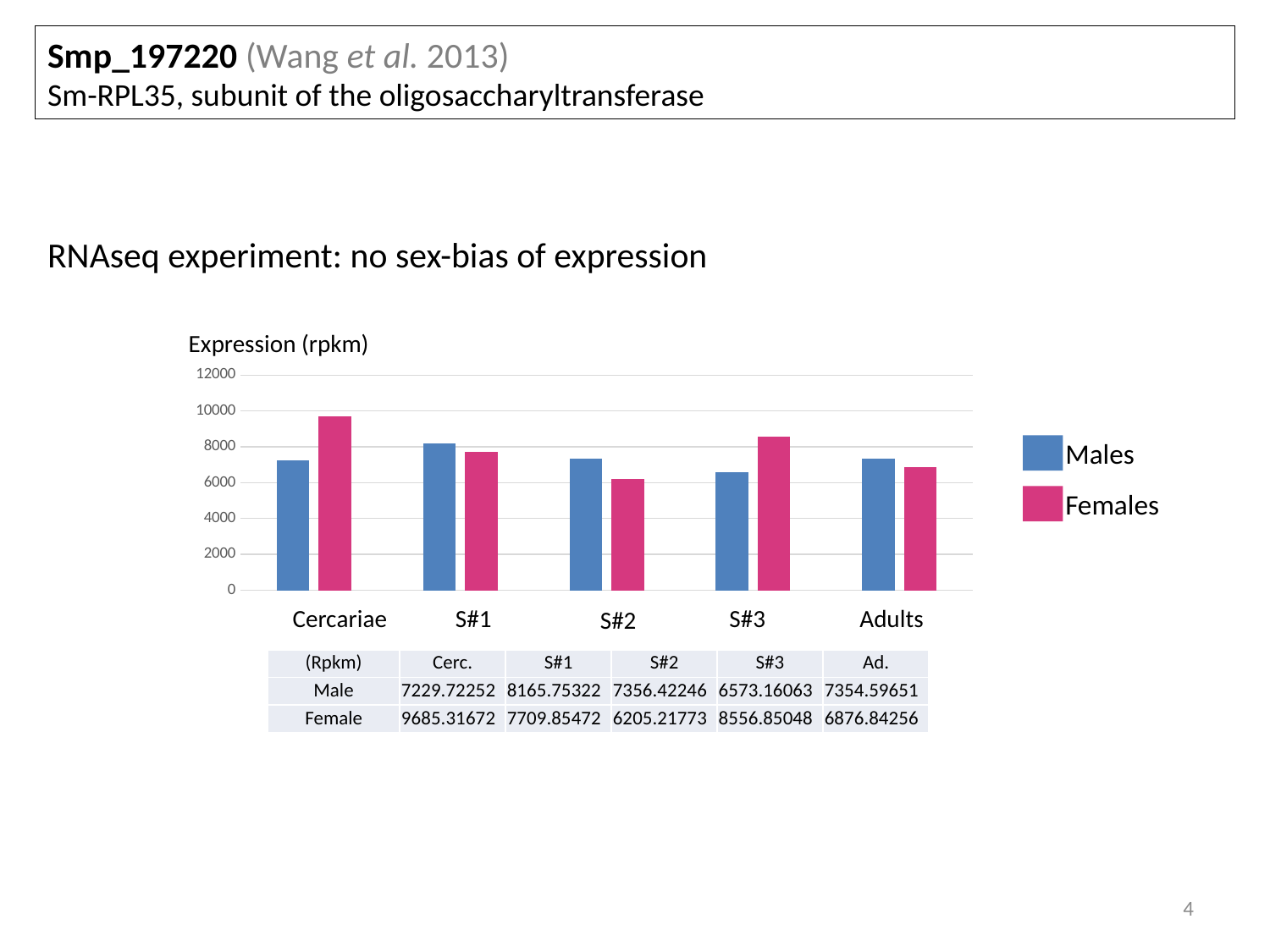

Smp_197220 (Wang et al. 2013)
Sm-RPL35, subunit of the oligosaccharyltransferase
RNAseq experiment: no sex-bias of expression
Expression (rpkm)
### Chart
| Category | | |
|---|---|---|Males
Females
Cercariae
S#1
S#3
Adults
S#2
| (Rpkm) | Cerc. | S#1 | S#2 | S#3 | Ad. |
| --- | --- | --- | --- | --- | --- |
| Male | 7229.72252 | 8165.75322 | 7356.42246 | 6573.16063 | 7354.59651 |
| Female | 9685.31672 | 7709.85472 | 6205.21773 | 8556.85048 | 6876.84256 |
4

## Slide 5
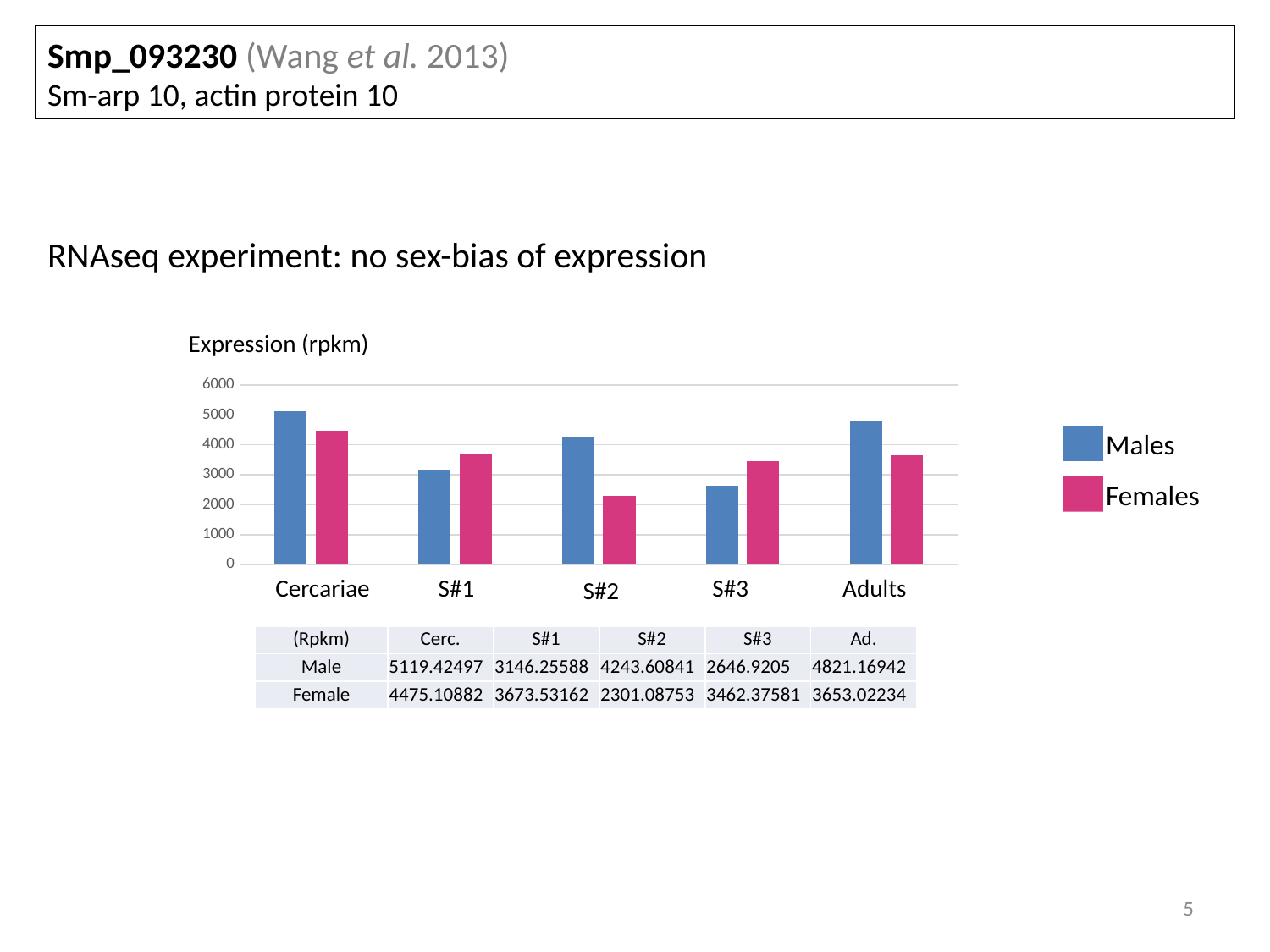

Smp_093230 (Wang et al. 2013)
Sm-arp 10, actin protein 10
RNAseq experiment: no sex-bias of expression
Expression (rpkm)
### Chart
| Category | | |
|---|---|---|Males
Females
Cercariae
S#1
S#3
Adults
S#2
| (Rpkm) | Cerc. | S#1 | S#2 | S#3 | Ad. |
| --- | --- | --- | --- | --- | --- |
| Male | 5119.42497 | 3146.25588 | 4243.60841 | 2646.9205 | 4821.16942 |
| Female | 4475.10882 | 3673.53162 | 2301.08753 | 3462.37581 | 3653.02234 |
5

## Slide 6
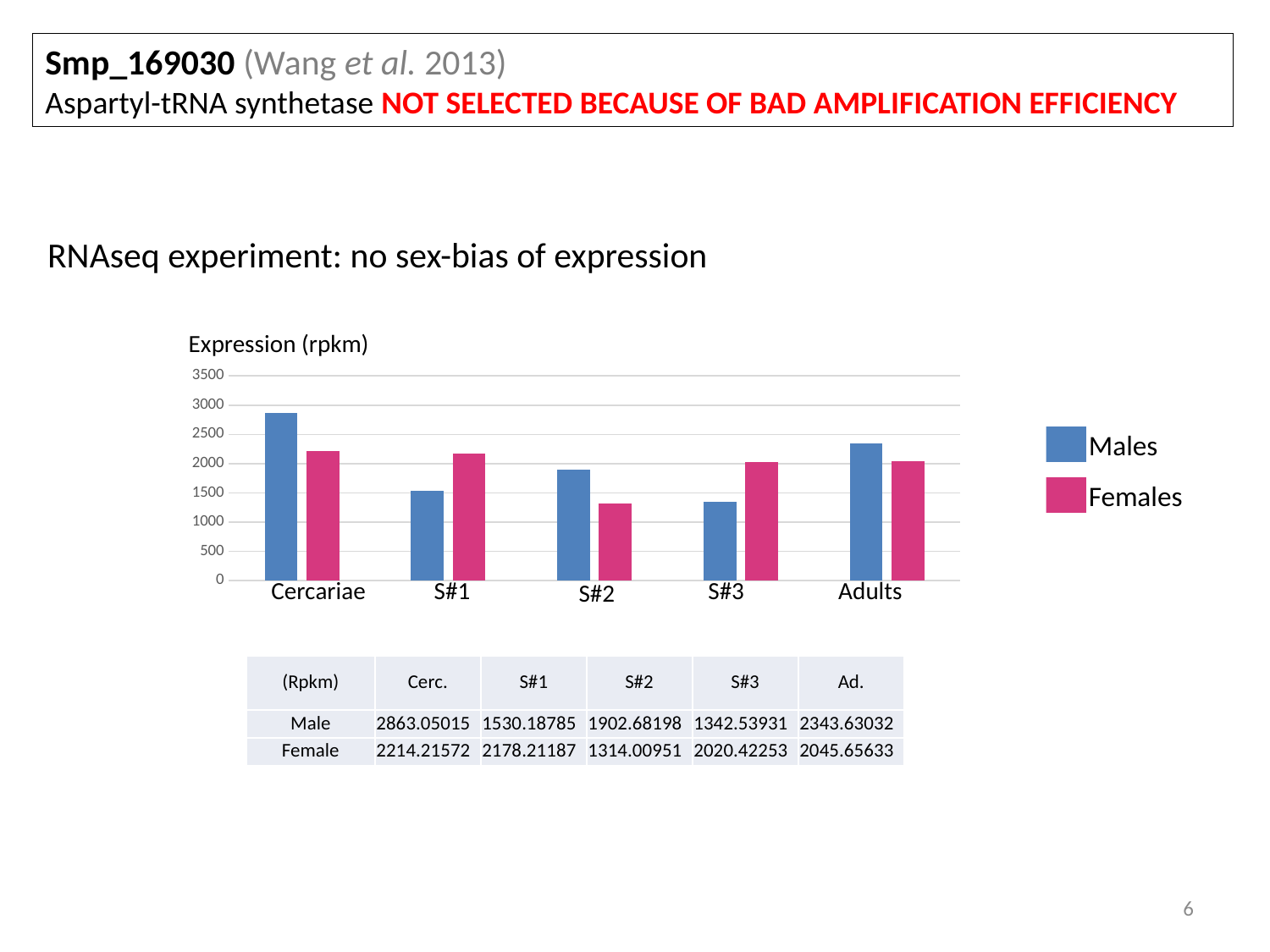

Smp_169030 (Wang et al. 2013)
Aspartyl-tRNA synthetase NOT SELECTED BECAUSE OF BAD AMPLIFICATION EFFICIENCY
RNAseq experiment: no sex-bias of expression
Expression (rpkm)
### Chart
| Category | | |
|---|---|---|Males
Females
Cercariae
S#1
S#3
Adults
S#2
| (Rpkm) | Cerc. | S#1 | S#2 | S#3 | Ad. |
| --- | --- | --- | --- | --- | --- |
| Male | 2863.05015 | 1530.18785 | 1902.68198 | 1342.53931 | 2343.63032 |
| Female | 2214.21572 | 2178.21187 | 1314.00951 | 2020.42253 | 2045.65633 |
6

## Slide 7
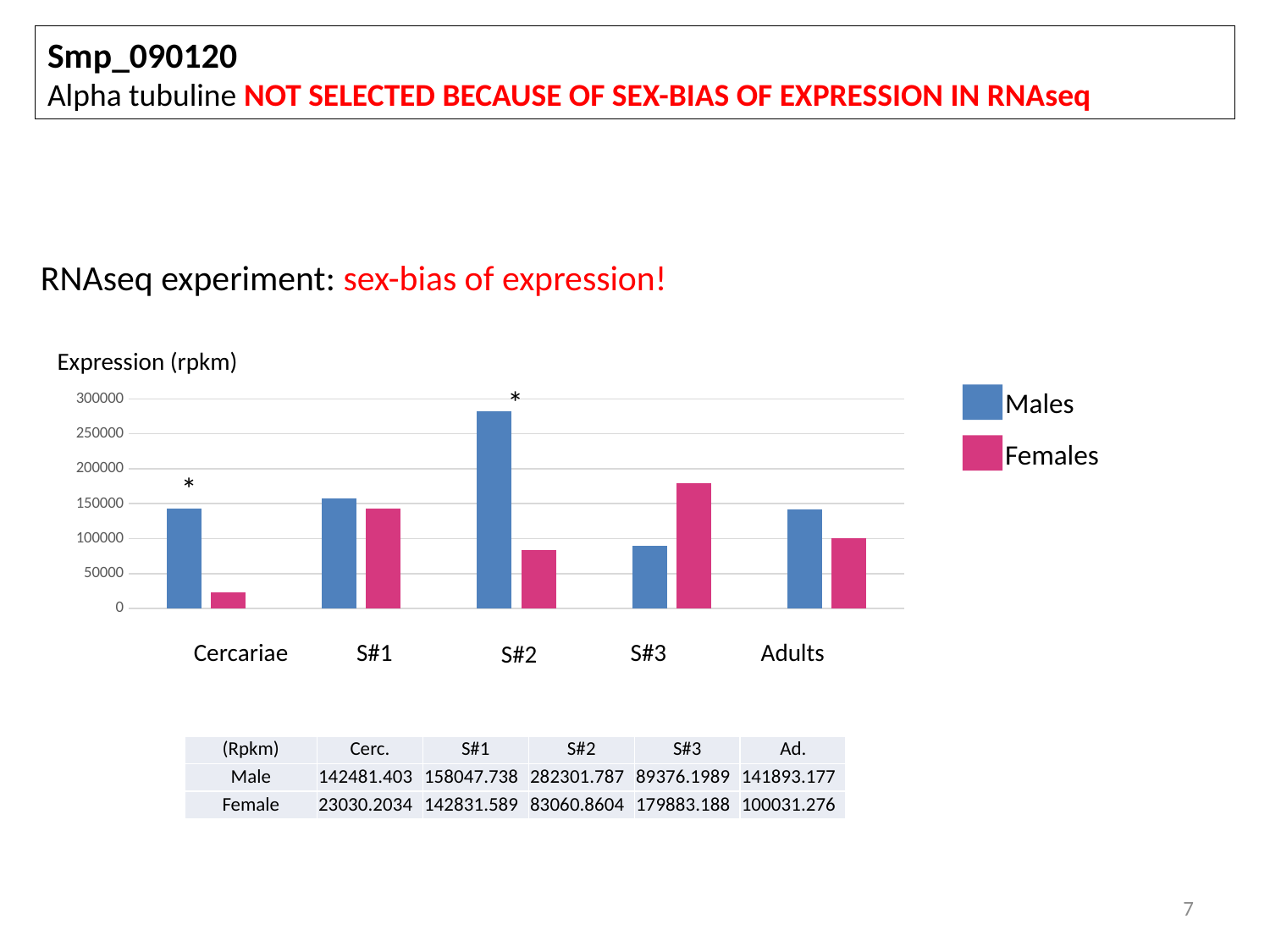

Smp_090120
Alpha tubuline NOT SELECTED BECAUSE OF SEX-BIAS OF EXPRESSION IN RNAseq
RNAseq experiment: sex-bias of expression!
Expression (rpkm)
### Chart
| Category | | |
|---|---|---|*
Males
Females
*
Cercariae
S#1
S#3
Adults
S#2
| (Rpkm) | Cerc. | S#1 | S#2 | S#3 | Ad. |
| --- | --- | --- | --- | --- | --- |
| Male | 142481.403 | 158047.738 | 282301.787 | 89376.1989 | 141893.177 |
| Female | 23030.2034 | 142831.589 | 83060.8604 | 179883.188 | 100031.276 |
7

## Slide 8
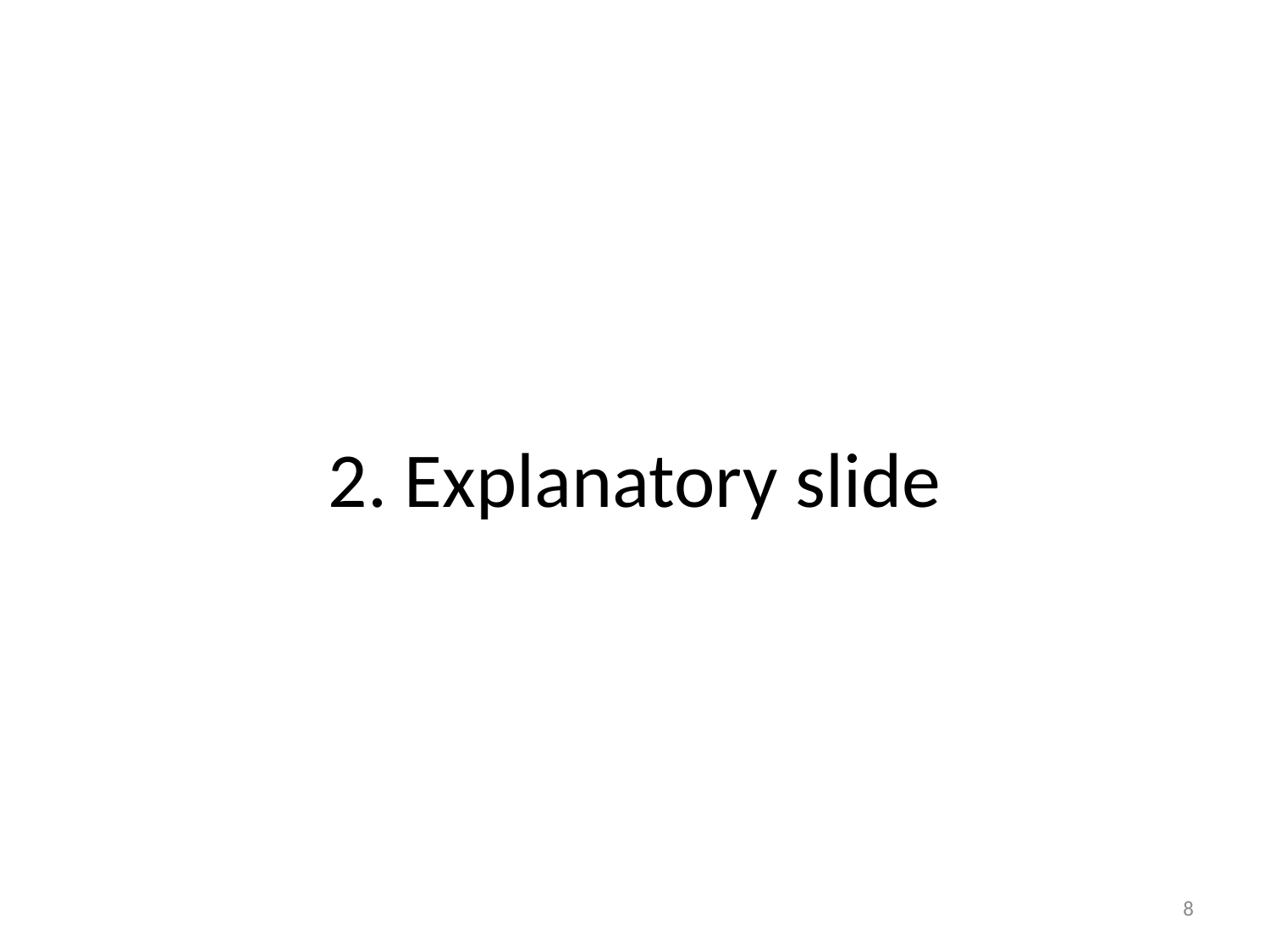

# 2. Explanatory slide
8

## Slide 9
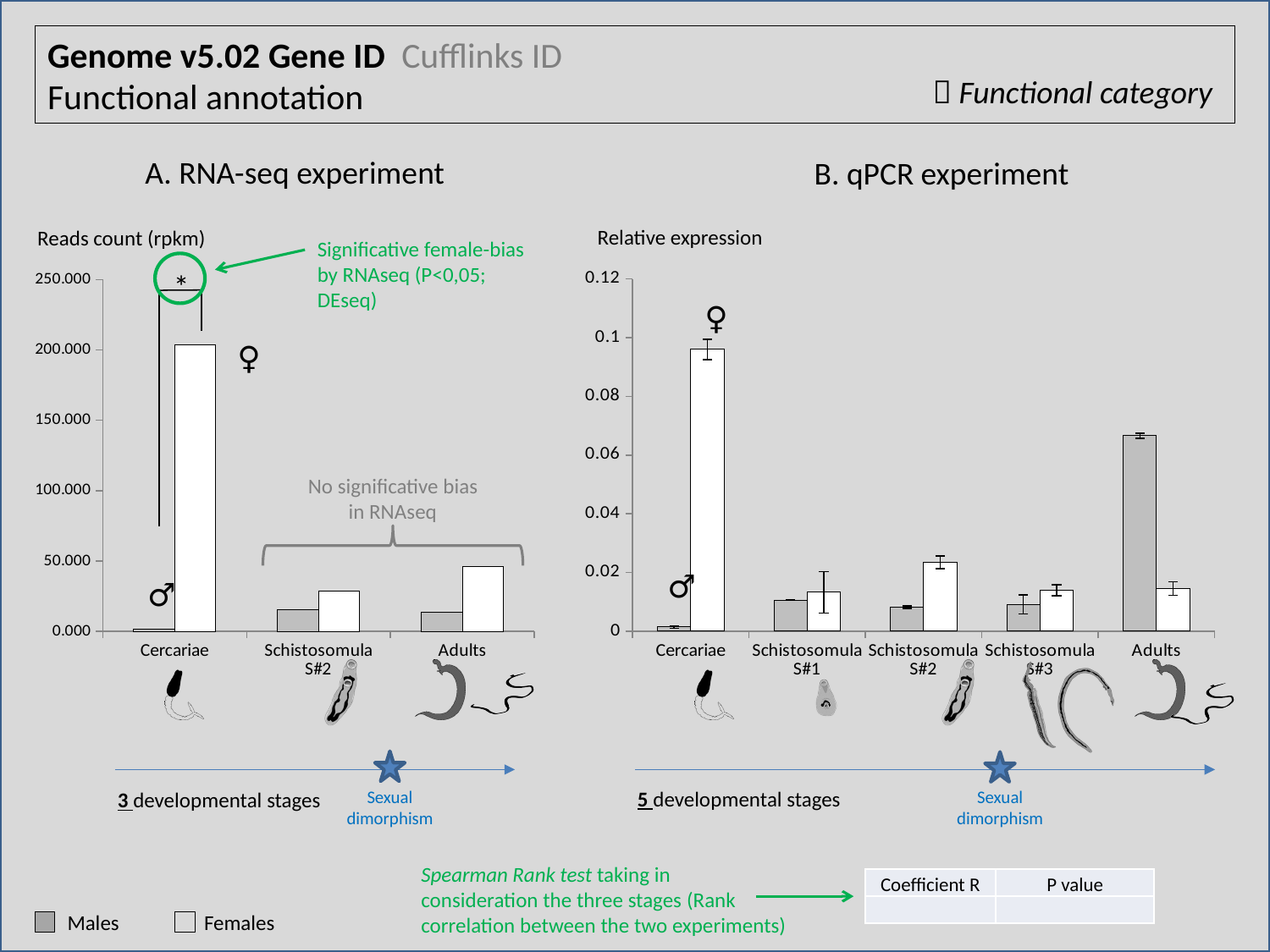

Genome v5.02 Gene ID Cufflinks ID
Functional annotation
 Functional category
A. RNA-seq experiment
B. qPCR experiment
Relative expression
Reads count (rpkm)
Significative female-bias by RNAseq (P<0,05; DEseq)
*
### Chart
| Category | Males | Females |
|---|---|---|
| Cercariae | 0.0014759630394028979 | 0.09599471887919866 |
| Schistosomula S#1 | 0.01059067352116443 | 0.013245970456836769 |
| Schistosomula S#2 | 0.008157840709857055 | 0.02346958066346376 |
| Schistosomula S#3 | 0.009084967849444271 | 0.013924328515274703 |
| Adults | 0.06662268770425851 | 0.01449354042972942 |
### Chart
| Category | Males | Females |
|---|---|---|
| Cercariae | 1.4389301532040482 | 203.39942372606873 |
| Schistosomula S#2 | 15.523024670776 | 28.727479606778587 |
| Adults | 13.445465669528316 | 46.048705997427774 |♀
♀
No significative bias in RNAseq
♂
♂
Sexual dimorphism
5 developmental stages
Sexual dimorphism
3 developmental stages
Spearman Rank test taking in consideration the three stages (Rank correlation between the two experiments)
| Coefficient R | P value |
| --- | --- |
| | |
9
Males 	 Females

## Slide 10
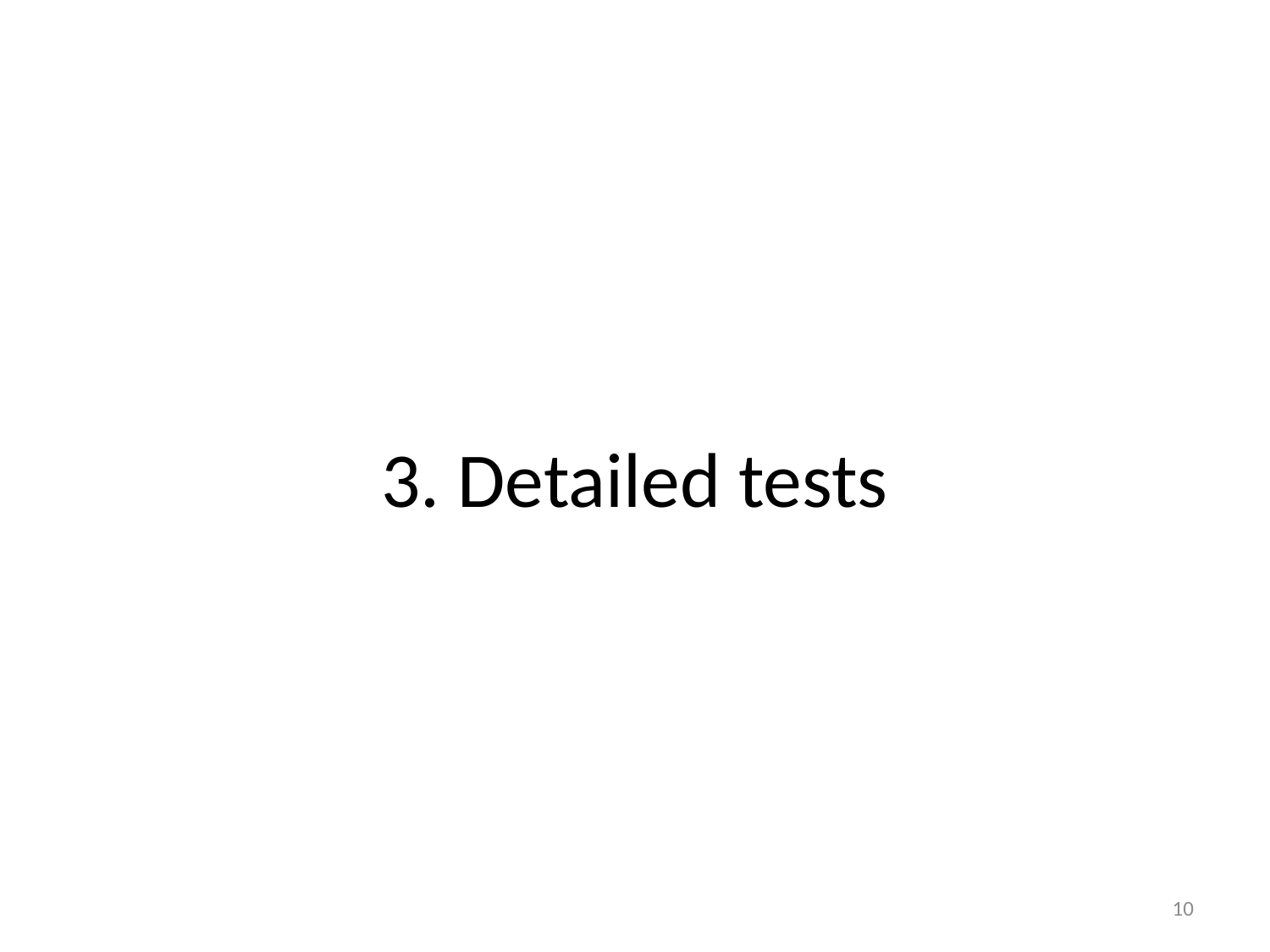

# 3. Detailed tests
10

## Slide 11
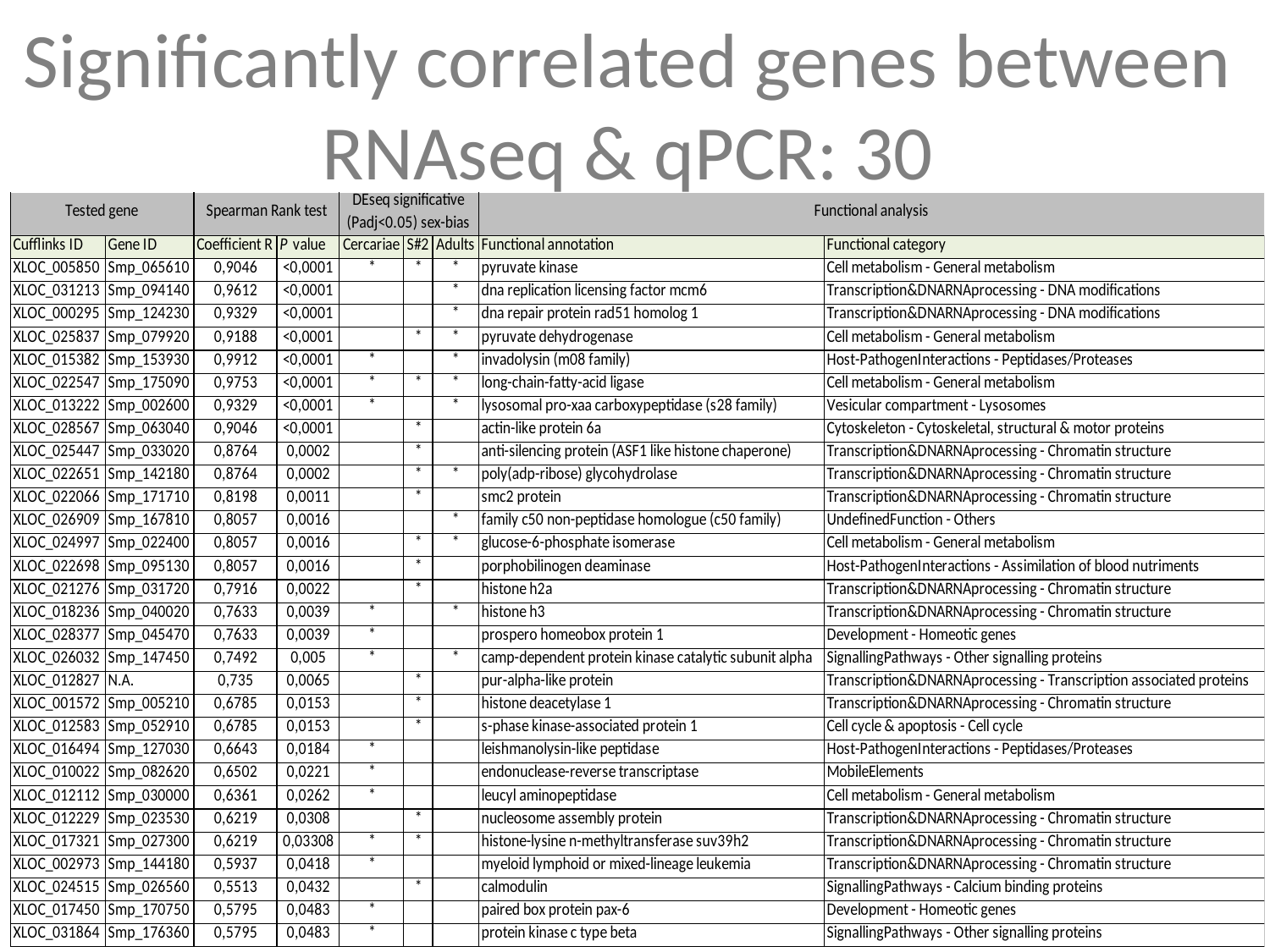

Significantly correlated genes between RNAseq & qPCR: 30

## Slide 12
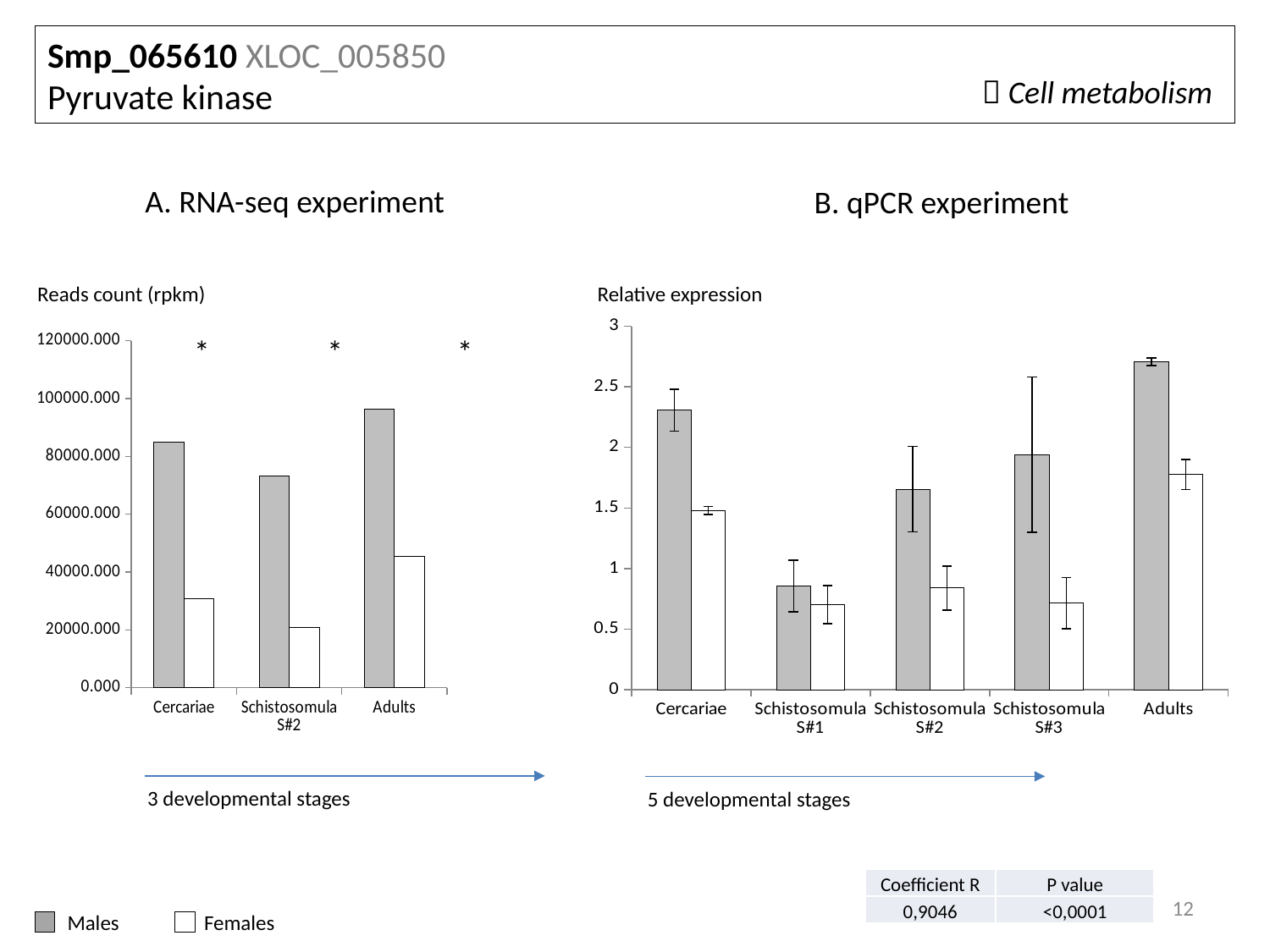

Smp_065610 XLOC_005850
Pyruvate kinase
 Cell metabolism
A. RNA-seq experiment
B. qPCR experiment
Relative expression
Reads count (rpkm)
### Chart
| Category | Males | Females |
|---|---|---|
| Cercariae | 2.3079428839054614 | 1.479947687415875 |
| Schistosomula S#1 | 0.8555026567866412 | 0.7037726048507384 |
| Schistosomula S#2 | 1.655472399175474 | 0.8387855578062025 |
| Schistosomula S#3 | 1.9405722744118465 | 0.7153695707198321 |
| Adults | 2.707702136987234 | 1.7765654138157299 |*
*
*
### Chart
| Category | Males | Females |
|---|---|---|
| Cercariae | 85022.3192280734 | 30616.1955832151 |
| Schistosomula S#2 | 73256.58411409108 | 20926.6234981403 |
| Adults | 96437.3352345741 | 45359.0426668067 |3 developmental stages
5 developmental stages
| Coefficient R | P value |
| --- | --- |
| 0,9046 | <0,0001 |
12
Males 	 Females

## Slide 13
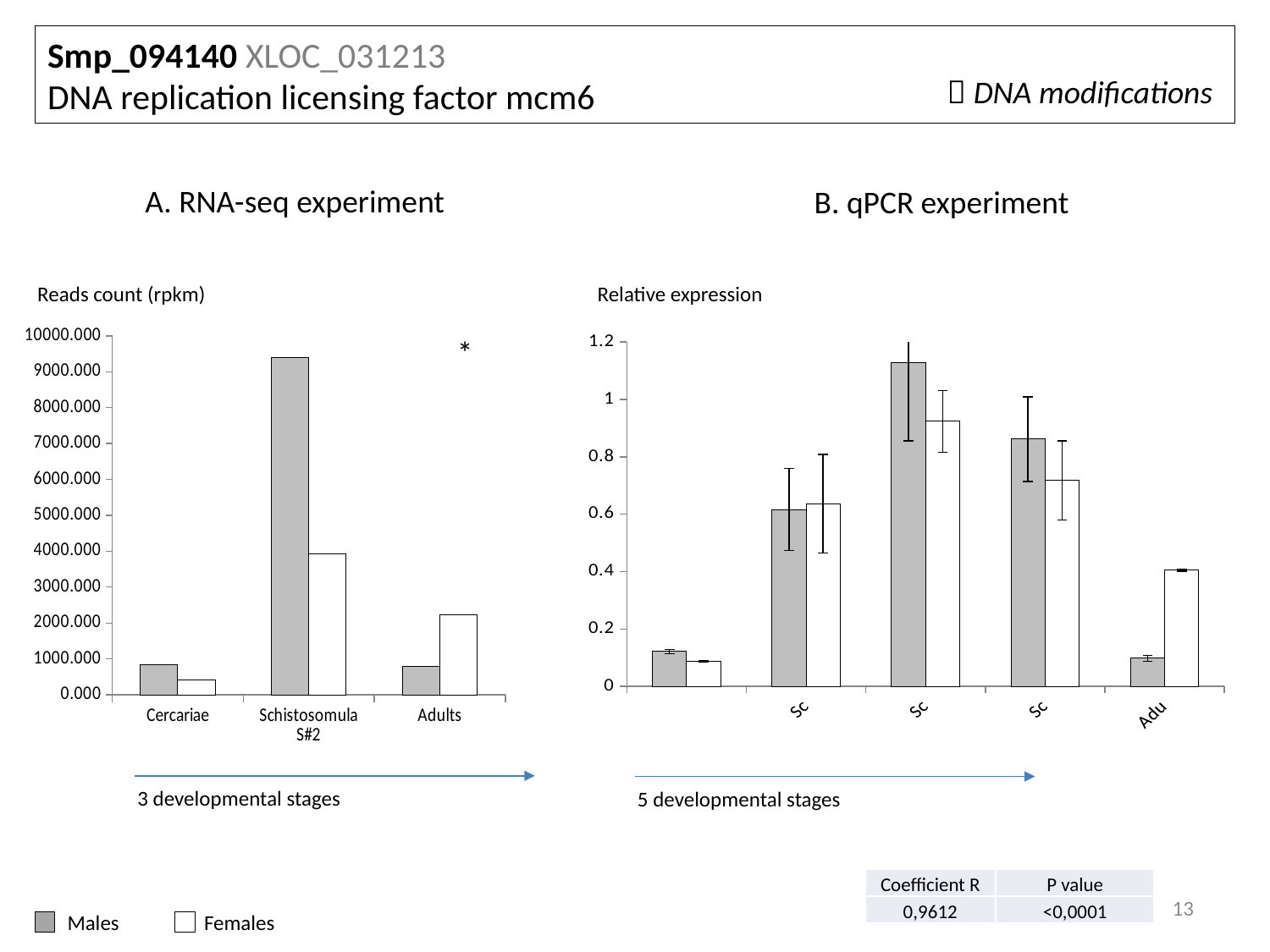

Smp_094140 XLOC_031213
DNA replication licensing factor mcm6
 DNA modifications
A. RNA-seq experiment
B. qPCR experiment
Relative expression
Reads count (rpkm)
### Chart
| Category | Males | Females |
|---|---|---|
| Cercariae | 847.7846653096985 | 415.220033204367 |
| Schistosomula S#2 | 9388.087447234415 | 3916.51965509533 |
| Adults | 783.748676624073 | 2232.2889046625387 |*
### Chart
| Category | Males | Females |
|---|---|---|
| Cercariae | 0.12120158133479594 | 0.08827820253082941 |
| Schistosomula S#1 | 0.61640033768259 | 0.6366523126065183 |
| Schistosomula S#2 | 1.1282544429316872 | 0.9235141263149854 |
| Schistosomula S#3 | 0.8615014502943208 | 0.7174202498255096 |
| Adults | 0.09773970006126773 | 0.40421579174925015 |3 developmental stages
5 developmental stages
| Coefficient R | P value |
| --- | --- |
| 0,9612 | <0,0001 |
13
Males 	 Females

## Slide 14
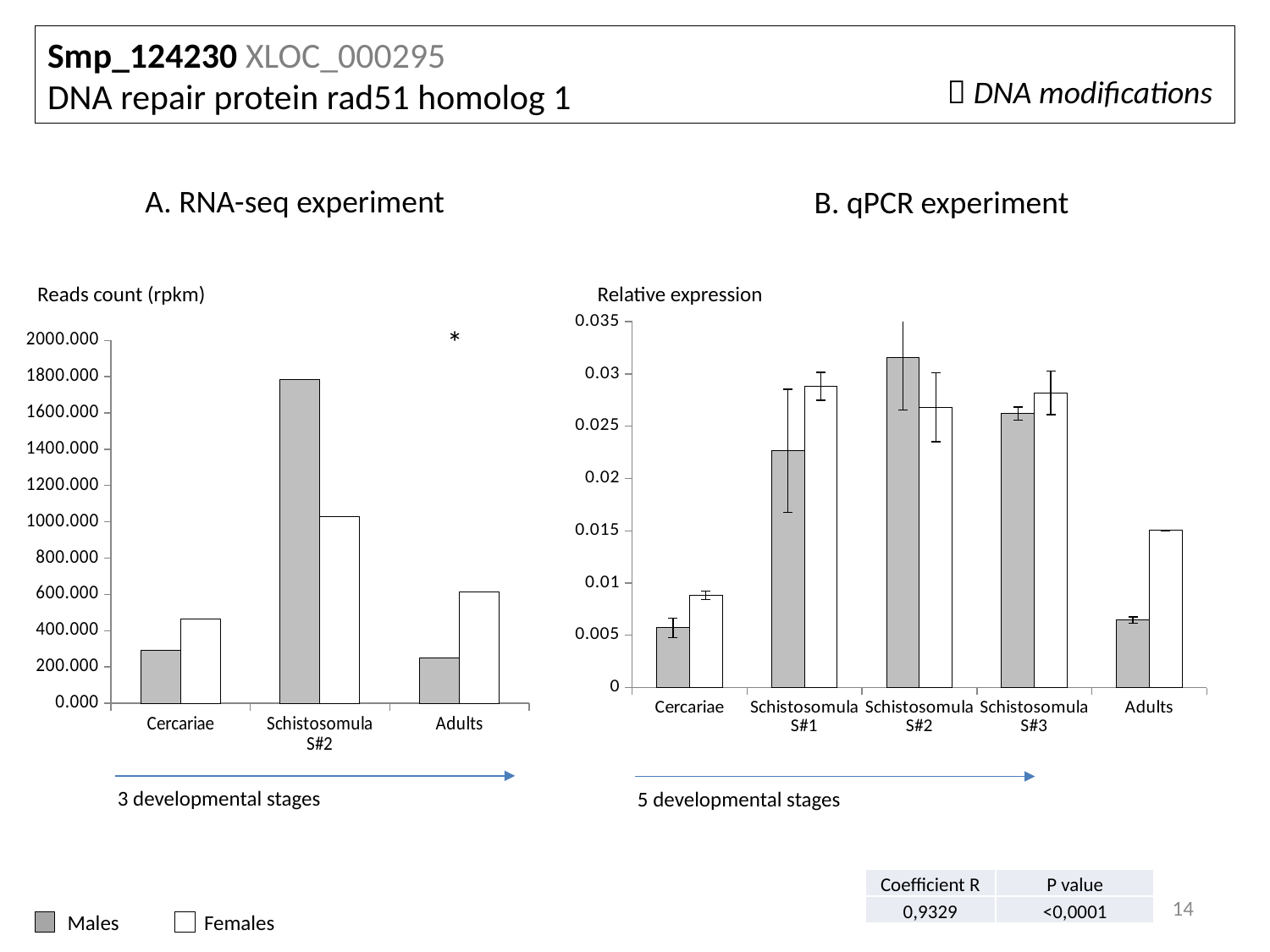

Smp_124230 XLOC_000295
DNA repair protein rad51 homolog 1
 DNA modifications
A. RNA-seq experiment
B. qPCR experiment
Relative expression
Reads count (rpkm)
### Chart
| Category | Males | Females |
|---|---|---|
| Cercariae | 0.00572667943388843 | 0.008830509628395775 |
| Schistosomula S#1 | 0.022638544386039787 | 0.028813921681730657 |
| Schistosomula S#2 | 0.031573378579608 | 0.02682203454190951 |
| Schistosomula S#3 | 0.026213869861820394 | 0.028193640156732314 |
| Adults | 0.006444978965186648 | 0.015021245841193508 |*
### Chart
| Category | Males | Females |
|---|---|---|
| Cercariae | 290.93785841175 | 465.662539146131 |
| Schistosomula S#2 | 1781.9672263769799 | 1027.7732668546798 |
| Adults | 247.18675322745398 | 614.759066093152 |3 developmental stages
5 developmental stages
| Coefficient R | P value |
| --- | --- |
| 0,9329 | <0,0001 |
14
Males 	 Females

## Slide 15
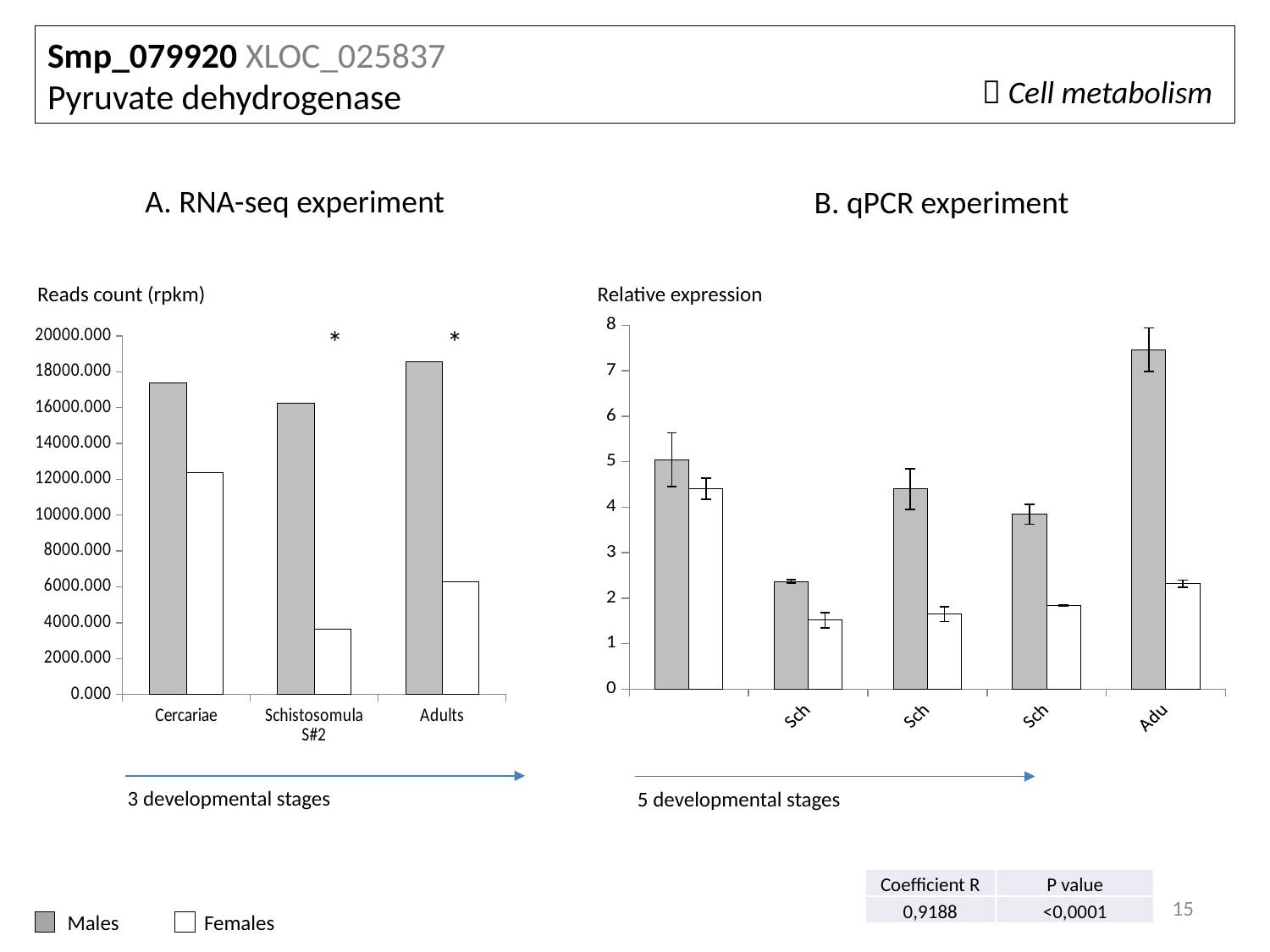

Smp_079920 XLOC_025837
Pyruvate dehydrogenase
 Cell metabolism
A. RNA-seq experiment
B. qPCR experiment
Relative expression
Reads count (rpkm)
*
*
### Chart
| Category | Males | Females |
|---|---|---|
| Cercariae | 5.045424651621835 | 4.406546321096579 |
| Schistosomula S#1 | 2.3692652104061 | 1.5162010271270092 |
| Schistosomula S#2 | 4.3988941580269625 | 1.6517956825770008 |
| Schistosomula S#3 | 3.8460266170802884 | 1.8389300631946544 |
| Adults | 7.464152584620965 | 2.3196963427689763 |
### Chart
| Category | Males | Females |
|---|---|---|
| Cercariae | 17355.9376525742 | 12349.179658895815 |
| Schistosomula S#2 | 16236.6465384269 | 3633.655377714061 |
| Adults | 18556.050664517796 | 6293.358310751351 |3 developmental stages
5 developmental stages
| Coefficient R | P value |
| --- | --- |
| 0,9188 | <0,0001 |
15
Males 	 Females

## Slide 16
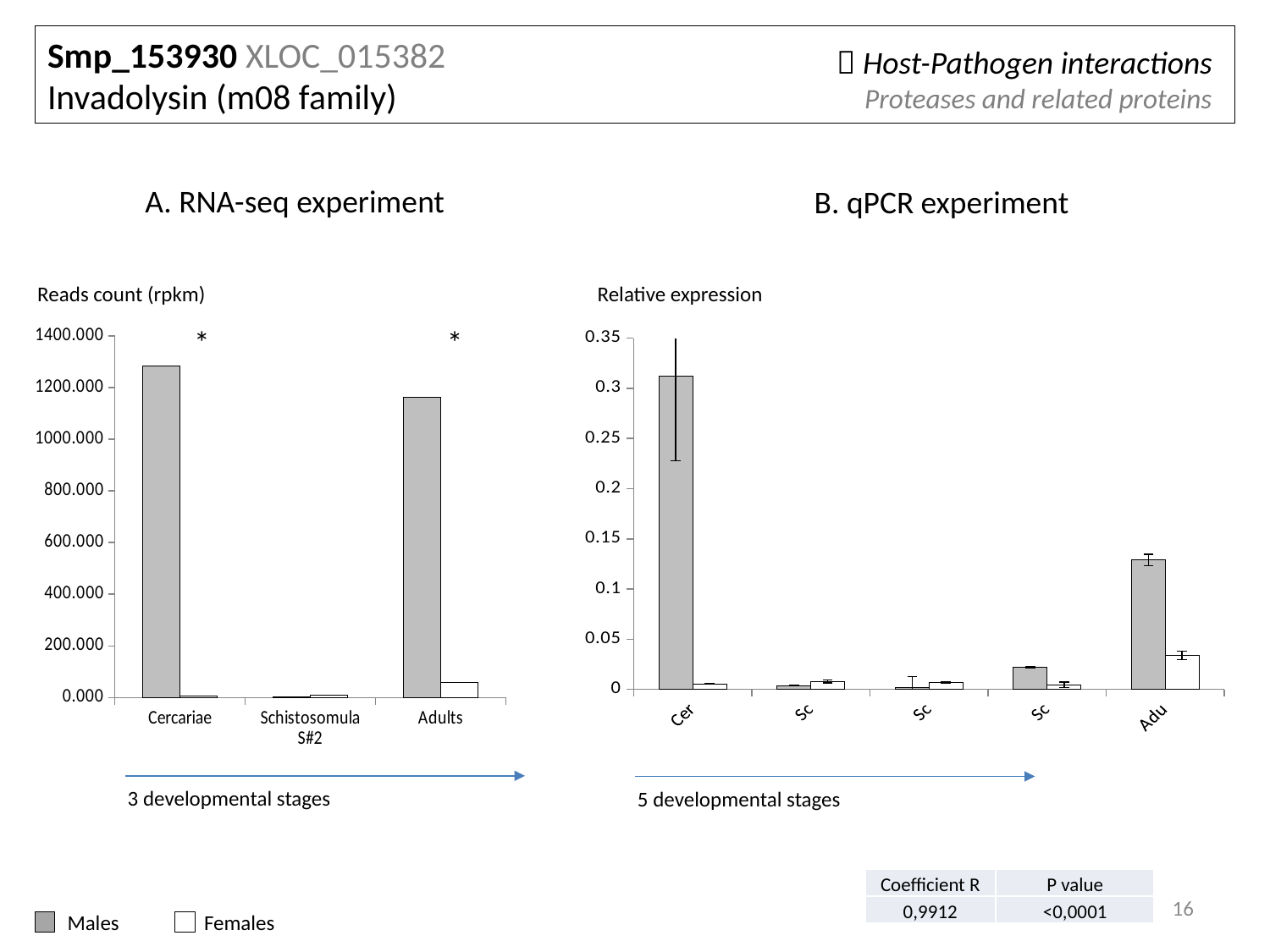

Smp_153930 XLOC_015382
Invadolysin (m08 family)
 Host-Pathogen interactions
Proteases and related proteins
A. RNA-seq experiment
B. qPCR experiment
Relative expression
Reads count (rpkm)
*
*
### Chart
| Category | Males | Females |
|---|---|---|
| Cercariae | 1283.88846044148 | 5.814937208978384 |
| Schistosomula S#2 | 3.347771682780654 | 9.093828987989408 |
| Adults | 1163.25047079391 | 58.860709803887296 |
### Chart
| Category | Males | Females |
|---|---|---|
| Cercariae | 0.3120047459455942 | 0.0054655373379084975 |
| Schistosomula S#1 | 0.00389553821263239 | 0.00789868282853499 |
| Schistosomula S#2 | 0.0022049312369908257 | 0.006739051336804367 |
| Schistosomula S#3 | 0.022203063899054493 | 0.0045132425971584796 |
| Adults | 0.1288133744042873 | 0.03370412537387957 |3 developmental stages
5 developmental stages
| Coefficient R | P value |
| --- | --- |
| 0,9912 | <0,0001 |
16
Males 	 Females

## Slide 17
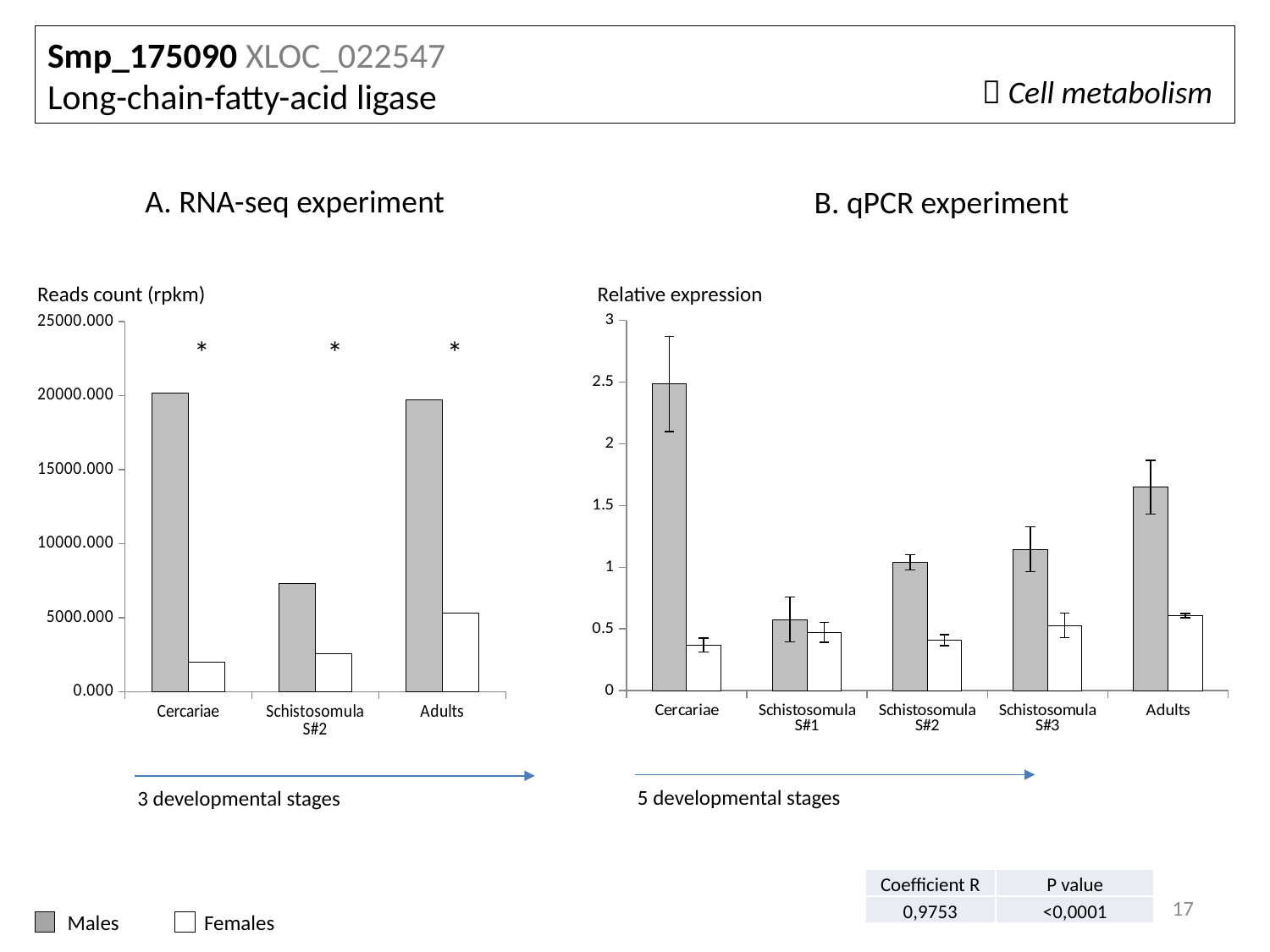

Smp_175090 XLOC_022547
Long-chain-fatty-acid ligase
 Cell metabolism
A. RNA-seq experiment
B. qPCR experiment
Relative expression
Reads count (rpkm)
### Chart
| Category | Males | Females |
|---|---|---|
| Cercariae | 20151.9095909435 | 1997.690427616798 |
| Schistosomula S#2 | 7315.265180478948 | 2562.53205378968 |
| Adults | 19717.0978799924 | 5316.73782040819 |
### Chart
| Category | Males | Females |
|---|---|---|
| Cercariae | 2.4839436243062742 | 0.37039922335954656 |
| Schistosomula S#1 | 0.5754115974432829 | 0.4724751723354308 |
| Schistosomula S#2 | 1.0394385608952723 | 0.40816010768177213 |
| Schistosomula S#3 | 1.146030031454446 | 0.5291926791128836 |
| Adults | 1.6495086380081547 | 0.6086898289888928 |*
*
*
5 developmental stages
3 developmental stages
| Coefficient R | P value |
| --- | --- |
| 0,9753 | <0,0001 |
17
Males 	 Females

## Slide 18
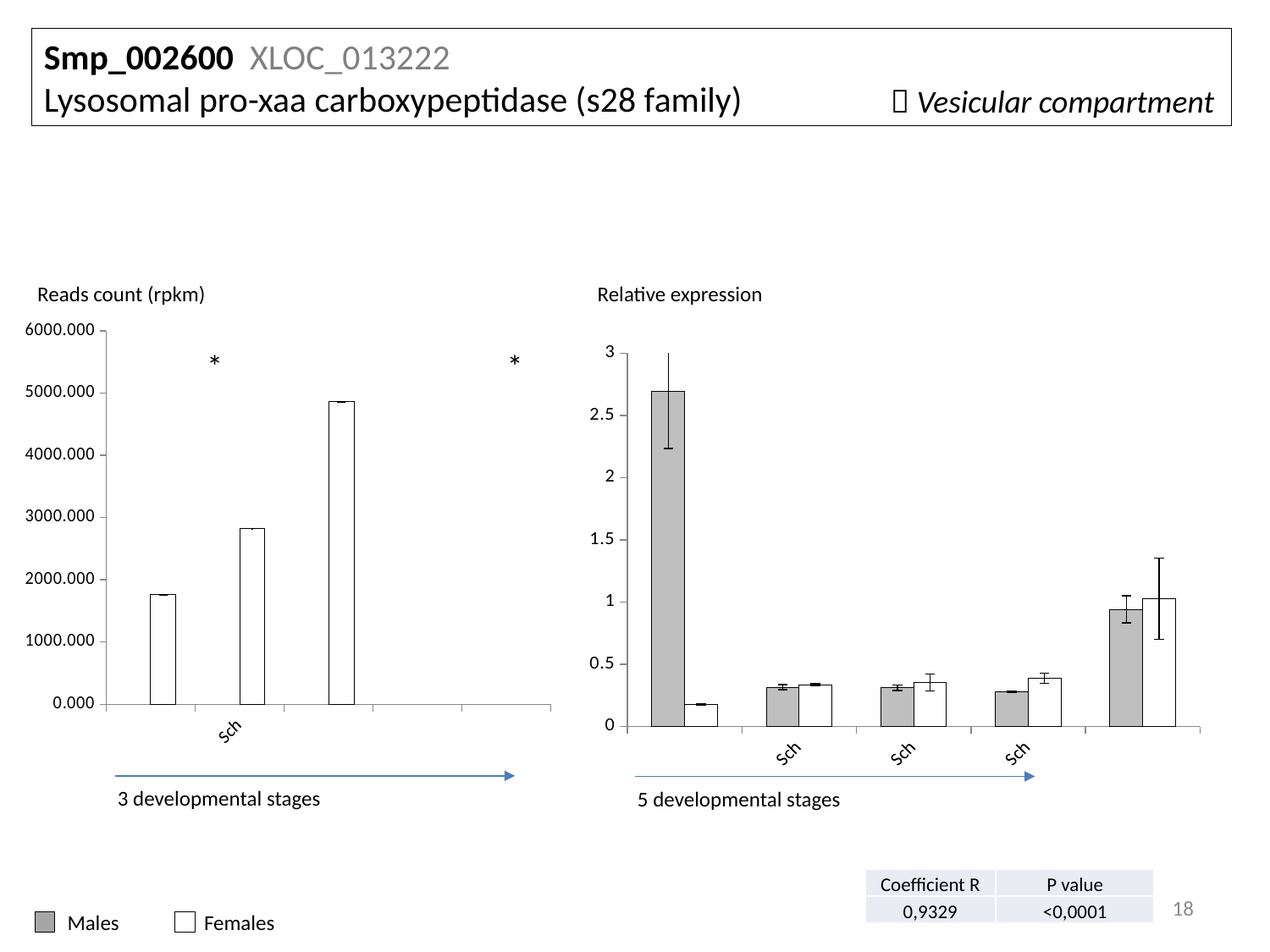

Smp_002600 XLOC_013222
Lysosomal pro-xaa carboxypeptidase (s28 family)
 Vesicular compartment
Relative expression
Reads count (rpkm)
### Chart
| Category | Males | Females |
|---|---|---|
| Cercariae | 25474.7575479083 | 1759.07856028337 |
| Schistosomula S#2 | 3245.45545037051 | 2820.54821279265 |
| Adults | 18368.4012603344 | 4860.39966953397 |*
*
### Chart
| Category | Males | Females |
|---|---|---|
| Cercariae | 2.691807885171351 | 0.17442391554234038 |
| Schistosomula S#1 | 0.31586341687803643 | 0.33544410875909336 |
| Schistosomula S#2 | 0.31188252152437196 | 0.35369180532903693 |
| Schistosomula S#3 | 0.2766698164972071 | 0.38807532308107573 |
| Adults | 0.9421079535394519 | 1.026252862418443 |3 developmental stages
5 developmental stages
| Coefficient R | P value |
| --- | --- |
| 0,9329 | <0,0001 |
18
Males 	 Females

## Slide 19
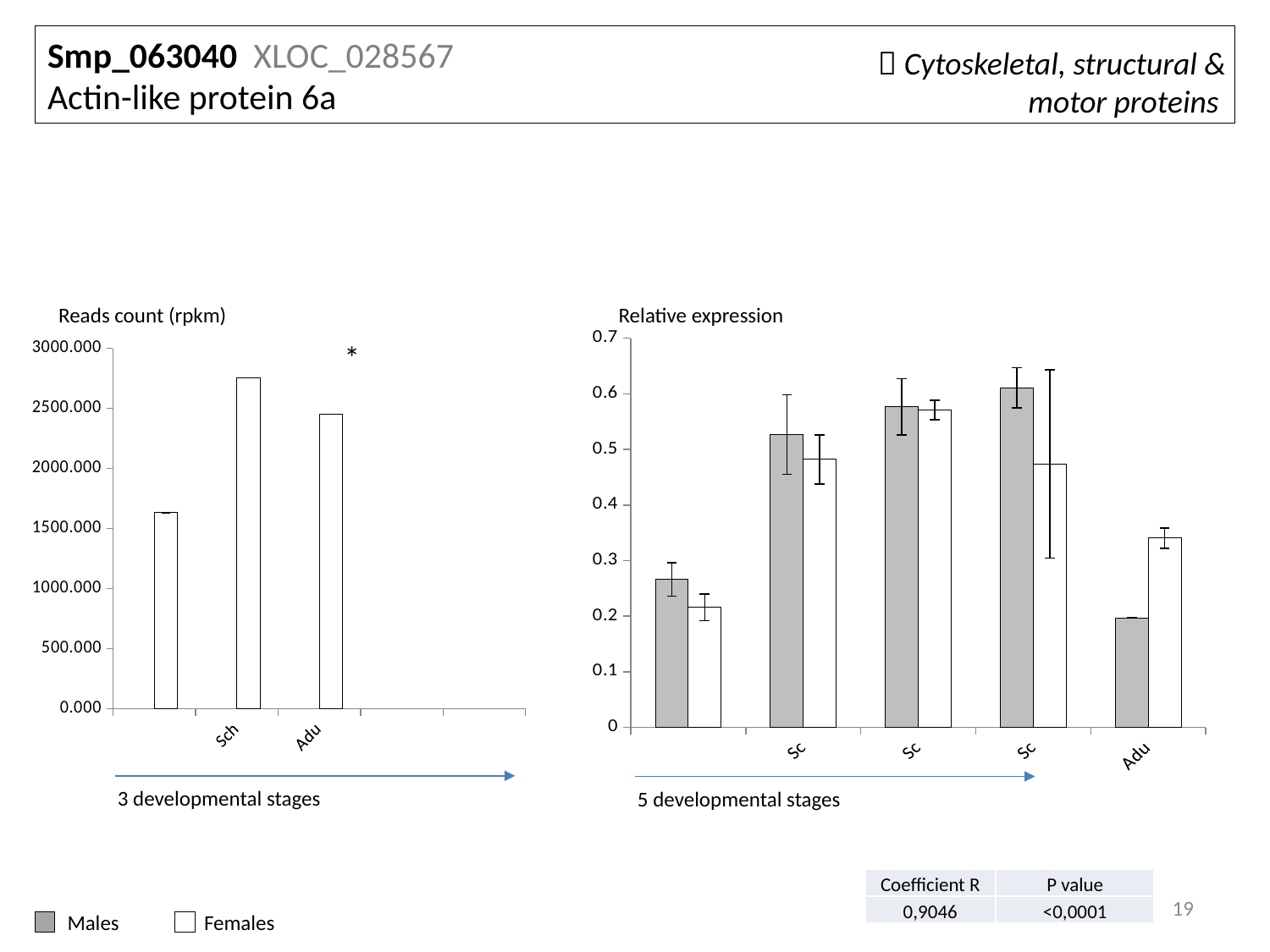

Smp_063040 XLOC_028567
Actin-like protein 6a
 Cytoskeletal, structural & motor proteins
Relative expression
Reads count (rpkm)
### Chart
| Category | Males | Females |
|---|---|---|
| Cercariae | 0.26598190091493773 | 0.21562449296363578 |
| Schistosomula S#1 | 0.5267696359773845 | 0.4821029227679038 |
| Schistosomula S#2 | 0.5766249433967922 | 0.570930290306358 |
| Schistosomula S#3 | 0.6107520853046684 | 0.47392159756632474 |
| Adults | 0.19675572416321616 | 0.3403851730895856 |*
### Chart
| Category | Males | Females |
|---|---|---|
| Cercariae | 2212.39832855207 | 1631.83846723592 |
| Schistosomula S#2 | 6306.92128474258 | 2754.77206133558 |
| Adults | 2148.67551266718 | 2453.58158067764 |3 developmental stages
5 developmental stages
| Coefficient R | P value |
| --- | --- |
| 0,9046 | <0,0001 |
19
Males 	 Females

## Slide 20
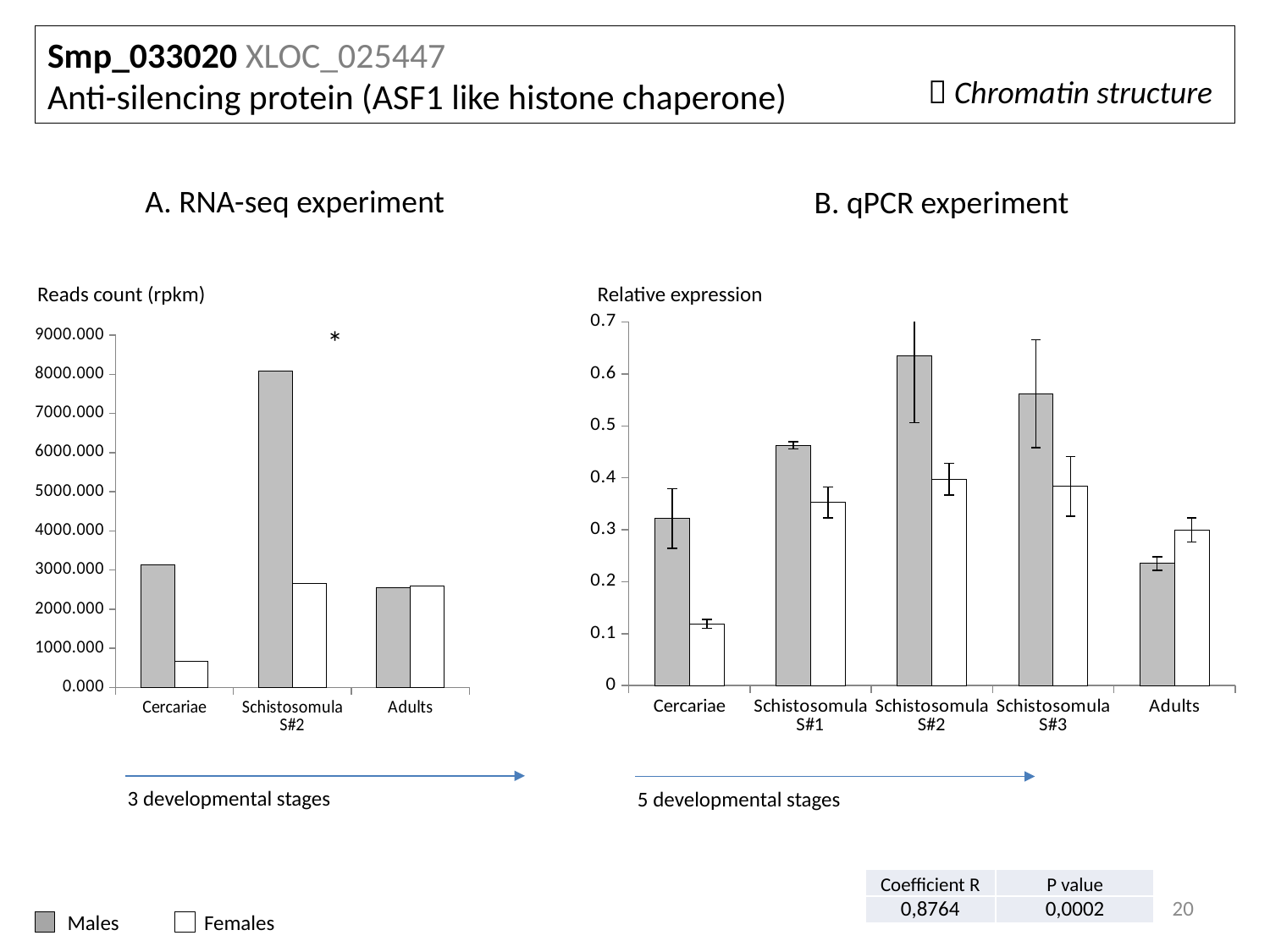

Smp_033020 XLOC_025447
Anti-silencing protein (ASF1 like histone chaperone)
 Chromatin structure
A. RNA-seq experiment
B. qPCR experiment
Relative expression
Reads count (rpkm)
### Chart
| Category | Males | Females |
|---|---|---|
| Cercariae | 0.321579247003677 | 0.1185732574430868 |
| Schistosomula S#1 | 0.46229928942019605 | 0.3527026442443951 |
| Schistosomula S#2 | 0.6354887780265164 | 0.39767765770598185 |
| Schistosomula S#3 | 0.5619347706992474 | 0.38360924923401707 |
| Adults | 0.23516188493704215 | 0.2998987883758215 |*
### Chart
| Category | Males | Females |
|---|---|---|
| Cercariae | 3134.5122369812657 | 667.696410878496 |
| Schistosomula S#2 | 8091.554874889735 | 2660.15109504619 |
| Adults | 2551.955945576535 | 2581.2786686188133 |3 developmental stages
5 developmental stages
| Coefficient R | P value |
| --- | --- |
| 0,8764 | 0,0002 |
20
Males 	 Females

## Slide 21
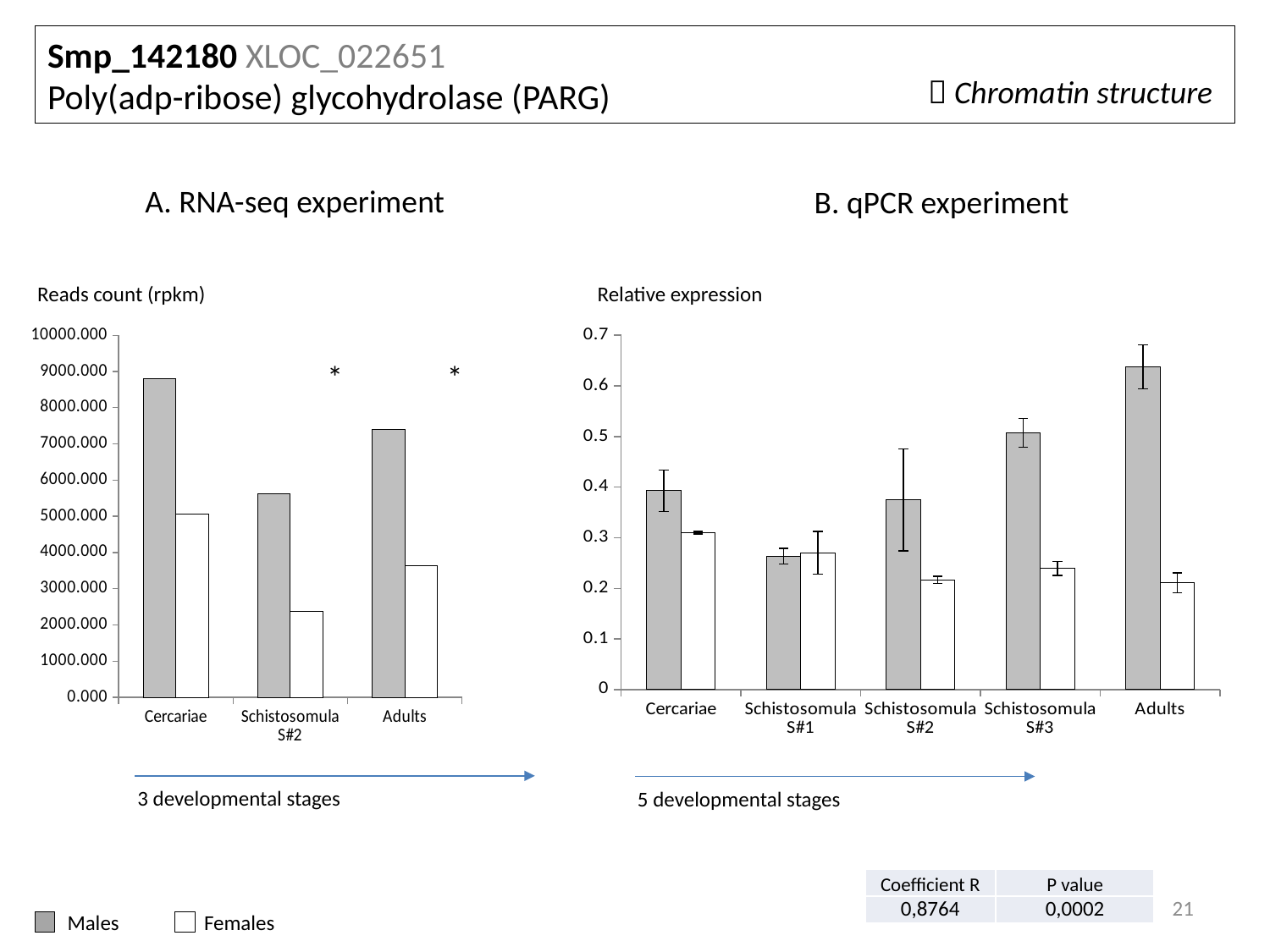

Smp_142180 XLOC_022651
Poly(adp-ribose) glycohydrolase (PARG)
 Chromatin structure
A. RNA-seq experiment
B. qPCR experiment
Relative expression
Reads count (rpkm)
### Chart
| Category | Males | Females |
|---|---|---|
| Cercariae | 0.3930838041359501 | 0.30997279015246165 |
| Schistosomula S#1 | 0.263653236089782 | 0.2705918490267505 |
| Schistosomula S#2 | 0.3749998848198487 | 0.2170657651771905 |
| Schistosomula S#3 | 0.5072838272904269 | 0.2395959460774846 |
| Adults | 0.6374170941009839 | 0.21084361786895003 |
### Chart
| Category | Males | Females |
|---|---|---|
| Cercariae | 8804.98359981553 | 5060.719086850892 |
| Schistosomula S#2 | 5629.35395087392 | 2364.6176323412856 |
| Adults | 7407.192222505744 | 3622.8349618320112 |*
*
3 developmental stages
5 developmental stages
| Coefficient R | P value |
| --- | --- |
| 0,8764 | 0,0002 |
21
Males 	 Females

## Slide 22
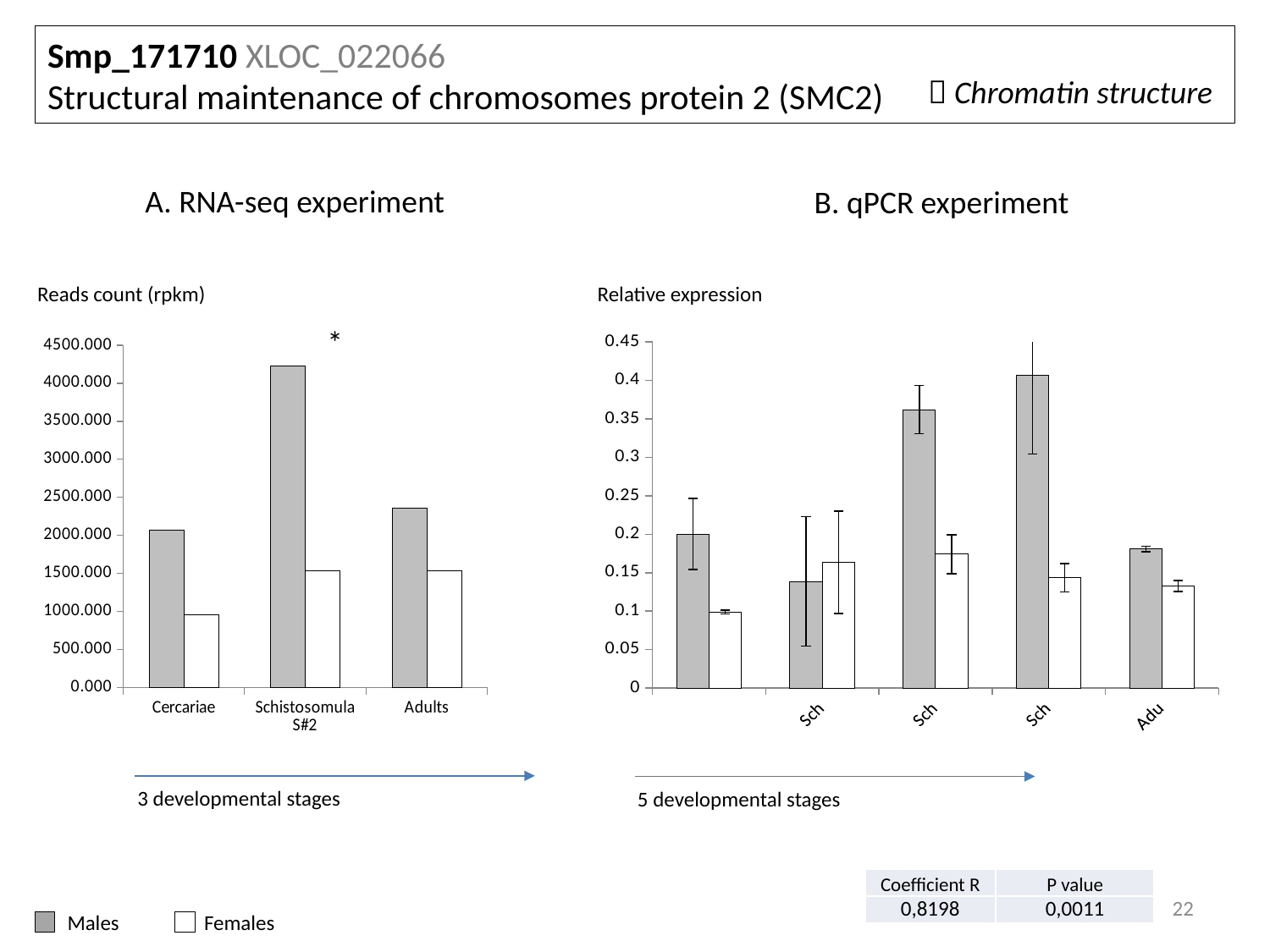

Smp_171710 XLOC_022066
Structural maintenance of chromosomes protein 2 (SMC2)
 Chromatin structure
A. RNA-seq experiment
B. qPCR experiment
Relative expression
Reads count (rpkm)
*
### Chart
| Category | Males | Females |
|---|---|---|
| Cercariae | 0.20025436536054522 | 0.09896577094426803 |
| Schistosomula S#1 | 0.13860465418384016 | 0.1634186361544465 |
| Schistosomula S#2 | 0.36201931903503654 | 0.17417840147025895 |
| Schistosomula S#3 | 0.40706678283734626 | 0.14344010803031 |
| Adults | 0.18077240576845088 | 0.1326054940599291 |
### Chart
| Category | Females | #REF! |
|---|---|---|
| Cercariae | 2068.1117814016898 | 954.041201523179 |
| Schistosomula S#2 | 4224.271362155761 | 1532.58106273678 |
| Adults | 2359.74024512064 | 1533.24252505307 |3 developmental stages
5 developmental stages
| Coefficient R | P value |
| --- | --- |
| 0,8198 | 0,0011 |
22
Males 	 Females

## Slide 23
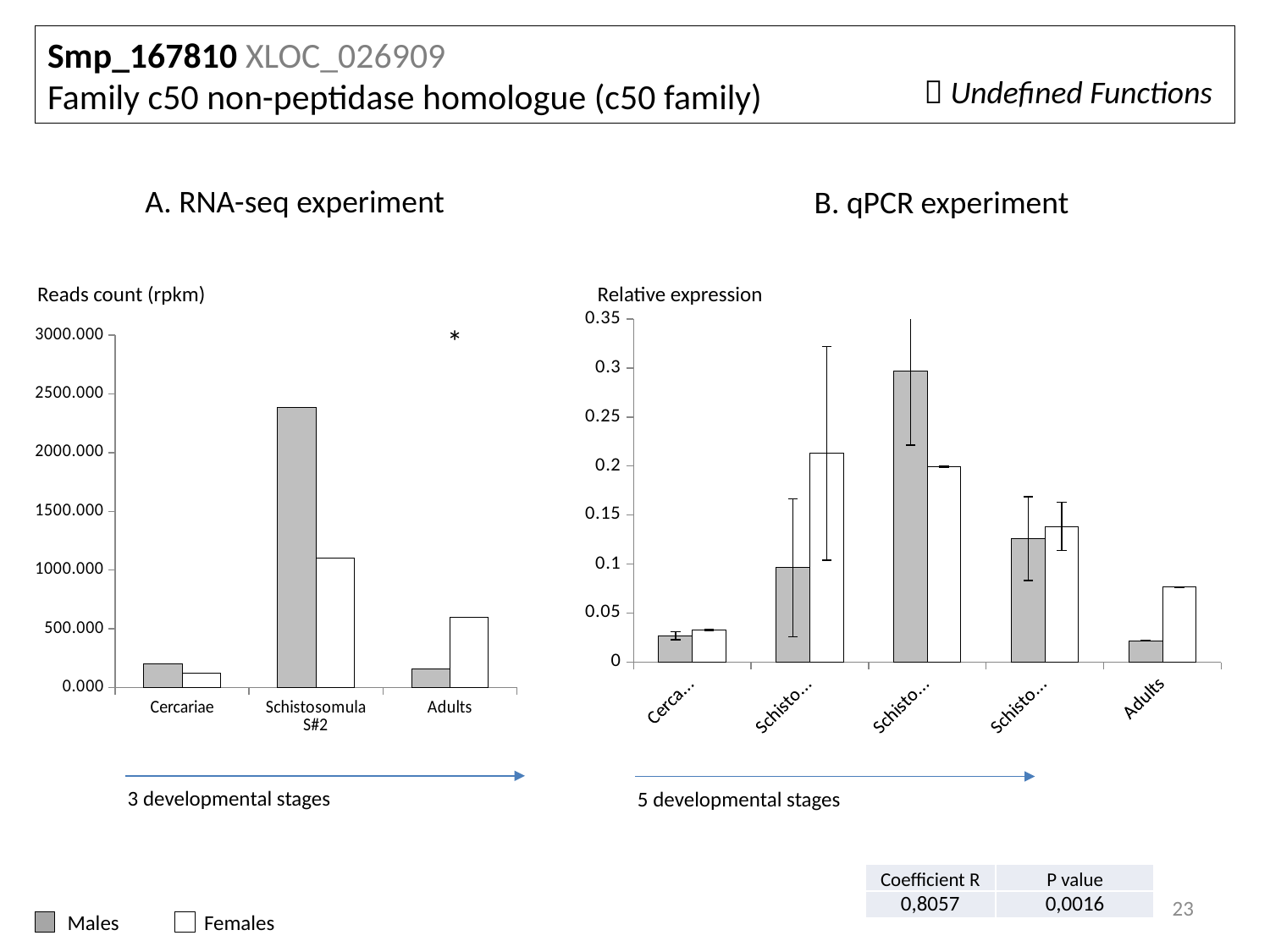

Smp_167810 XLOC_026909
Family c50 non-peptidase homologue (c50 family)
 Undefined Functions
A. RNA-seq experiment
B. qPCR experiment
### Chart
| Category | Males | Females |
|---|---|---|
| Cercariae | 0.02710582432363985 | 0.03287109062454525 |
| Schistosomula S#1 | 0.09622364644901457 | 0.21282230806433453 |
| Schistosomula S#2 | 0.29705663527476533 | 0.19914516465415832 |
| Schistosomula S#3 | 0.12599985480668663 | 0.13841635639837907 |
| Adults | 0.022017109071646456 | 0.07658195408987381 |Relative expression
Reads count (rpkm)
*
### Chart
| Category | Males | Females |
|---|---|---|
| Cercariae | 202.741135966685 | 124.490421415107 |
| Schistosomula S#2 | 2382.66901448526 | 1099.60890195357 |
| Adults | 160.38832756509245 | 595.5918796212305 |3 developmental stages
5 developmental stages
| Coefficient R | P value |
| --- | --- |
| 0,8057 | 0,0016 |
23
Males 	 Females

## Slide 24
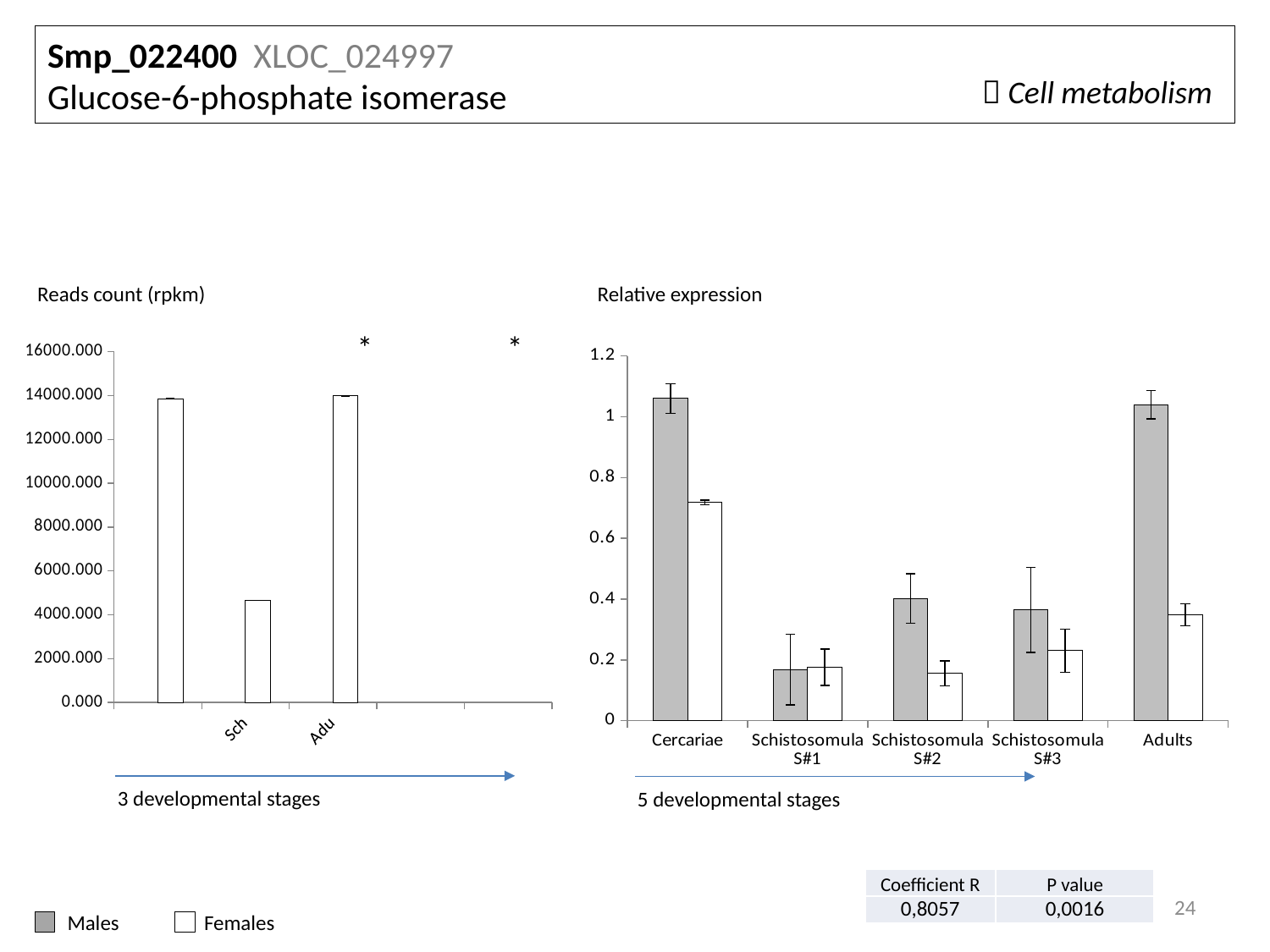

Smp_022400 XLOC_024997
Glucose-6-phosphate isomerase
 Cell metabolism
Relative expression
Reads count (rpkm)
*
*
### Chart
| Category | Males | Females |
|---|---|---|
| Cercariae | 32203.725492508 | 13855.0399352493 |
| Schistosomula S#2 | 14663.6547188777 | 4654.62550086539 |
| Adults | 30690.6180998633 | 13984.9752704573 |
### Chart
| Category | Males | Females |
|---|---|---|
| Cercariae | 1.0600311798304947 | 0.7183387037111403 |
| Schistosomula S#1 | 0.1681759216846706 | 0.17581691489688914 |
| Schistosomula S#2 | 0.402596523389899 | 0.15582696523864487 |
| Schistosomula S#3 | 0.36479202310352415 | 0.2304624069772202 |
| Adults | 1.0395103518414186 | 0.3483001220224793 |3 developmental stages
5 developmental stages
| Coefficient R | P value |
| --- | --- |
| 0,8057 | 0,0016 |
24
Males 	 Females

## Slide 25
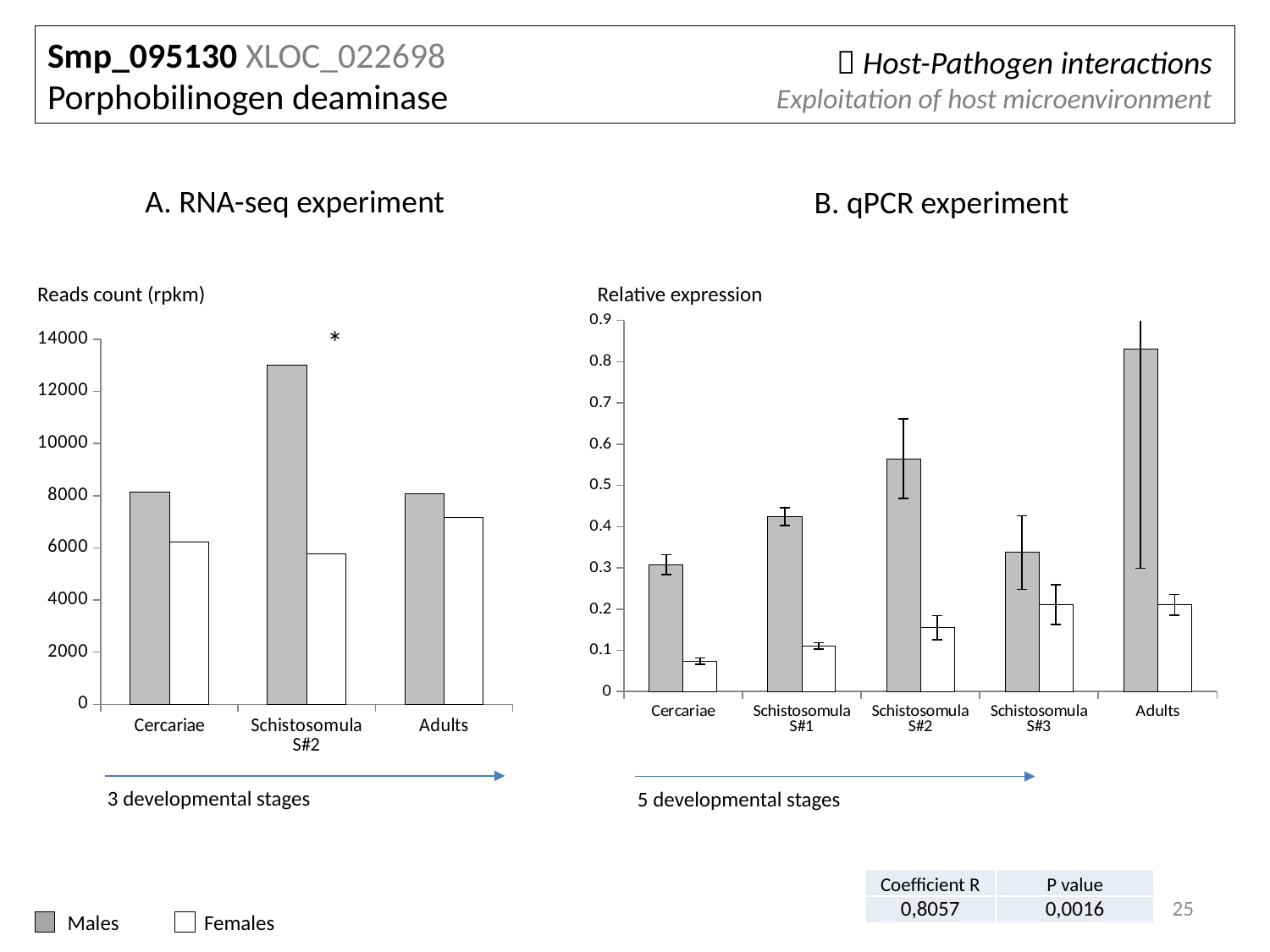

Smp_095130 XLOC_022698
Porphobilinogen deaminase
 Host-Pathogen interactions
Exploitation of host microenvironment
A. RNA-seq experiment
B. qPCR experiment
Relative expression
Reads count (rpkm)
### Chart
| Category | Males | Females |
|---|---|---|
| Cercariae | 0.30771955784494043 | 0.07409490430541503 |
| Schistosomula S#1 | 0.42441115435626103 | 0.11033546283413245 |
| Schistosomula S#2 | 0.5648195729207514 | 0.15474667088466637 |
| Schistosomula S#3 | 0.3370386656640273 | 0.21058108990499397 |
| Adults | 0.8302899373578192 | 0.21072955494432996 |*
### Chart
| Category | Males | Females |
|---|---|---|
| Cercariae | 8144.376517374169 | 6225.53505462948 |
| Schistosomula S#2 | 13003.5952719459 | 5772.65922851187 |
| Adults | 8063.5943475100385 | 7157.483147116359 |3 developmental stages
5 developmental stages
| Coefficient R | P value |
| --- | --- |
| 0,8057 | 0,0016 |
25
Males 	 Females

## Slide 26
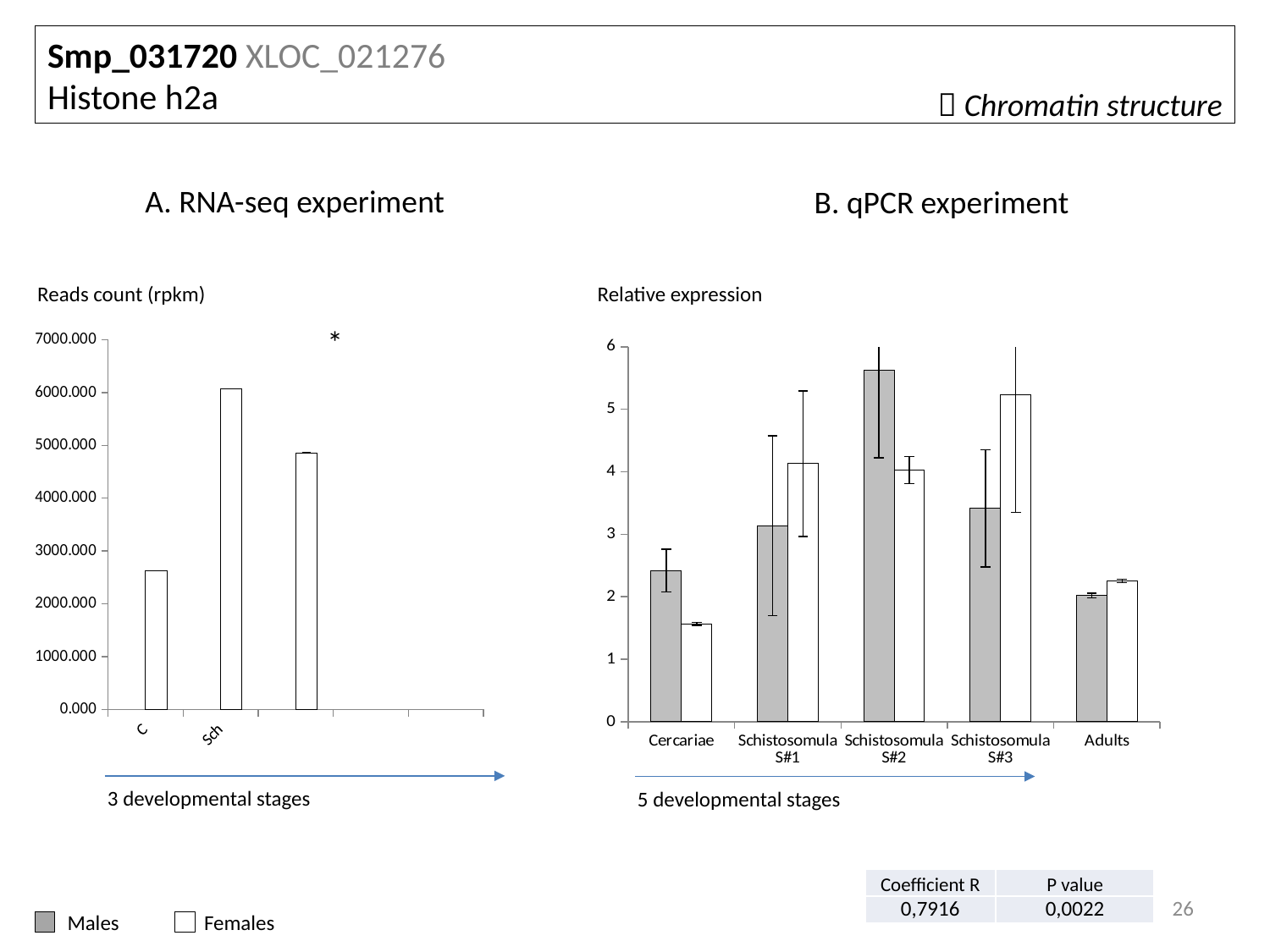

Smp_031720 XLOC_021276
Histone h2a
 Chromatin structure
A. RNA-seq experiment
B. qPCR experiment
Relative expression
Reads count (rpkm)
*
### Chart
| Category | Males | Females |
|---|---|---|
| Cercariae | 5085.47454274593 | 2625.7435773176 |
| Schistosomula S#2 | 13372.6785470465 | 6071.73459767964 |
| Adults | 5933.86499787023 | 4860.04260124461 |
### Chart
| Category | Males | Females |
|---|---|---|
| Cercariae | 2.419121907452 | 1.5668533427265494 |
| Schistosomula S#1 | 3.1371650620089575 | 4.129327135395816 |
| Schistosomula S#2 | 5.624528033209264 | 4.029462525492737 |
| Schistosomula S#3 | 3.4165660900225125 | 5.226751775669433 |
| Adults | 2.0204822911985385 | 2.255199292552884 |3 developmental stages
5 developmental stages
| Coefficient R | P value |
| --- | --- |
| 0,7916 | 0,0022 |
26
Males 	 Females

## Slide 27
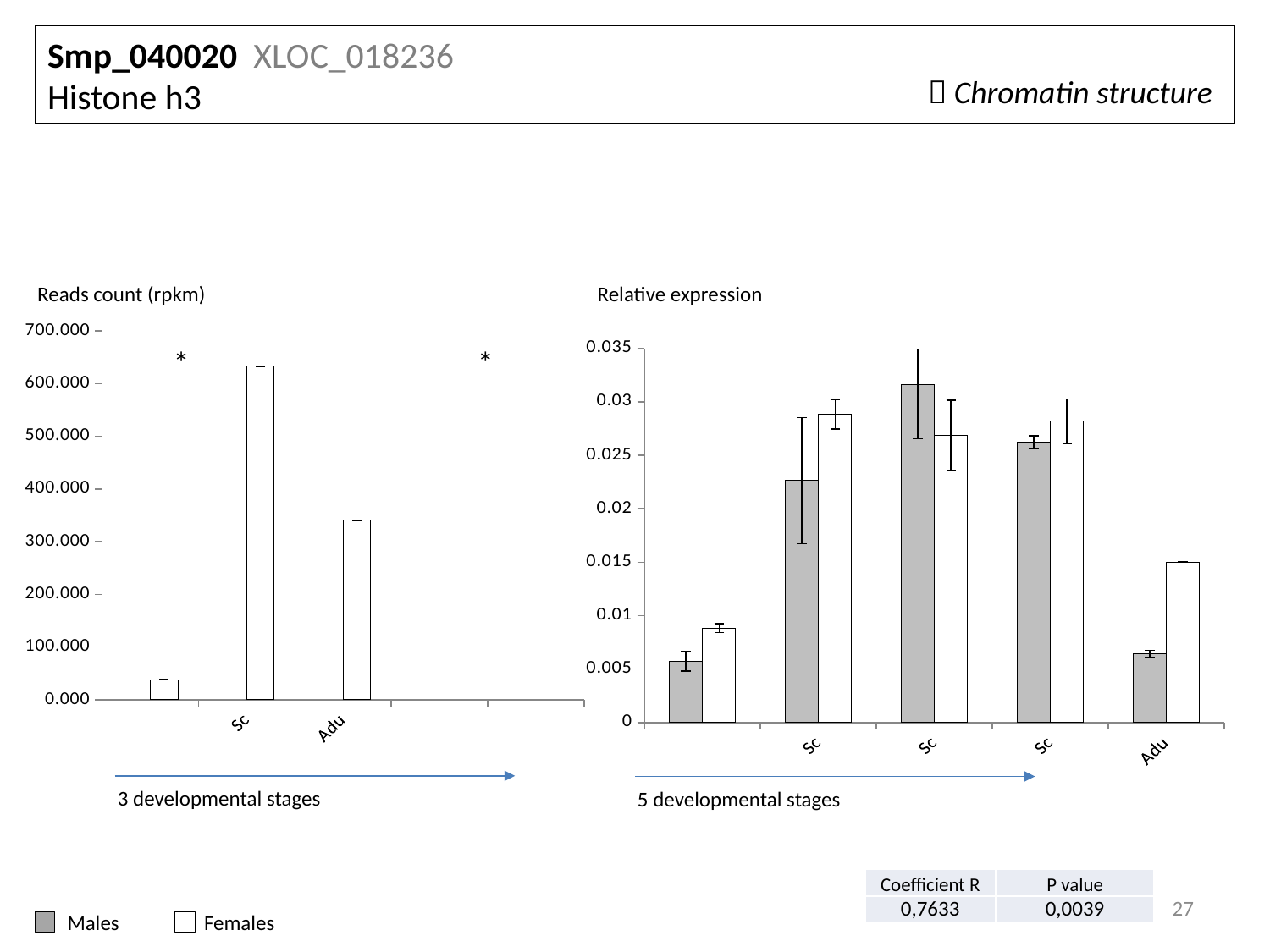

Smp_040020 XLOC_018236
Histone h3
 Chromatin structure
Relative expression
Reads count (rpkm)
### Chart
| Category | Males | Females |
|---|---|---|
| Cercariae | 134.52922974014 | 38.1886700444332 |
| Schistosomula S#2 | 988.997814569073 | 632.96252831787 |
| Adults | 111.706715263829 | 340.451799927713 |*
*
### Chart
| Category | Males | Females |
|---|---|---|
| Cercariae | 0.00572667943388843 | 0.008830509628395775 |
| Schistosomula S#1 | 0.022638544386039787 | 0.028813921681730657 |
| Schistosomula S#2 | 0.031573378579608 | 0.02682203454190951 |
| Schistosomula S#3 | 0.026213869861820394 | 0.028193640156732314 |
| Adults | 0.006444978965186648 | 0.015021245841193508 |3 developmental stages
5 developmental stages
| Coefficient R | P value |
| --- | --- |
| 0,7633 | 0,0039 |
27
Males 	 Females

## Slide 28
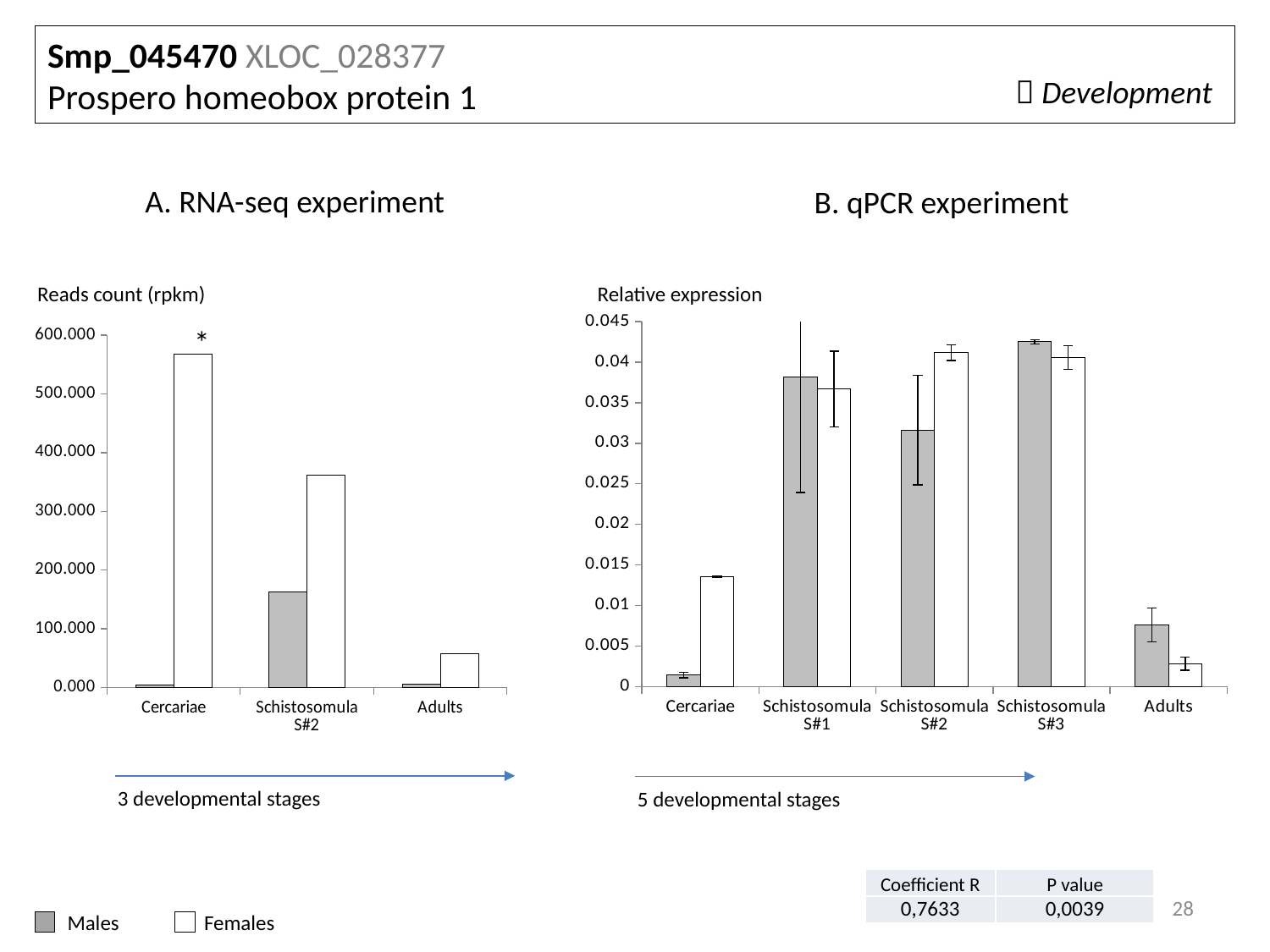

Smp_045470 XLOC_028377
Prospero homeobox protein 1
 Development
A. RNA-seq experiment
B. qPCR experiment
Relative expression
Reads count (rpkm)
### Chart
| Category | Males | Females |
|---|---|---|
| Cercariae | 0.0014270549415858194 | 0.01358063874344878 |
| Schistosomula S#1 | 0.03813484458171963 | 0.03666805852054063 |
| Schistosomula S#2 | 0.03163081216661372 | 0.041184243516935976 |
| Schistosomula S#3 | 0.042500008003263055 | 0.040590296736891185 |
| Adults | 0.007627357300862151 | 0.002835985122619648 |*
### Chart
| Category | Males | Females |
|---|---|---|
| Cercariae | 3.88841989598723 | 567.234599723751 |
| Schistosomula S#2 | 162.870213727885 | 361.149361342283 |
| Adults | 6.20856131365599 | 57.813626170666545 |3 developmental stages
5 developmental stages
| Coefficient R | P value |
| --- | --- |
| 0,7633 | 0,0039 |
28
Males 	 Females

## Slide 29
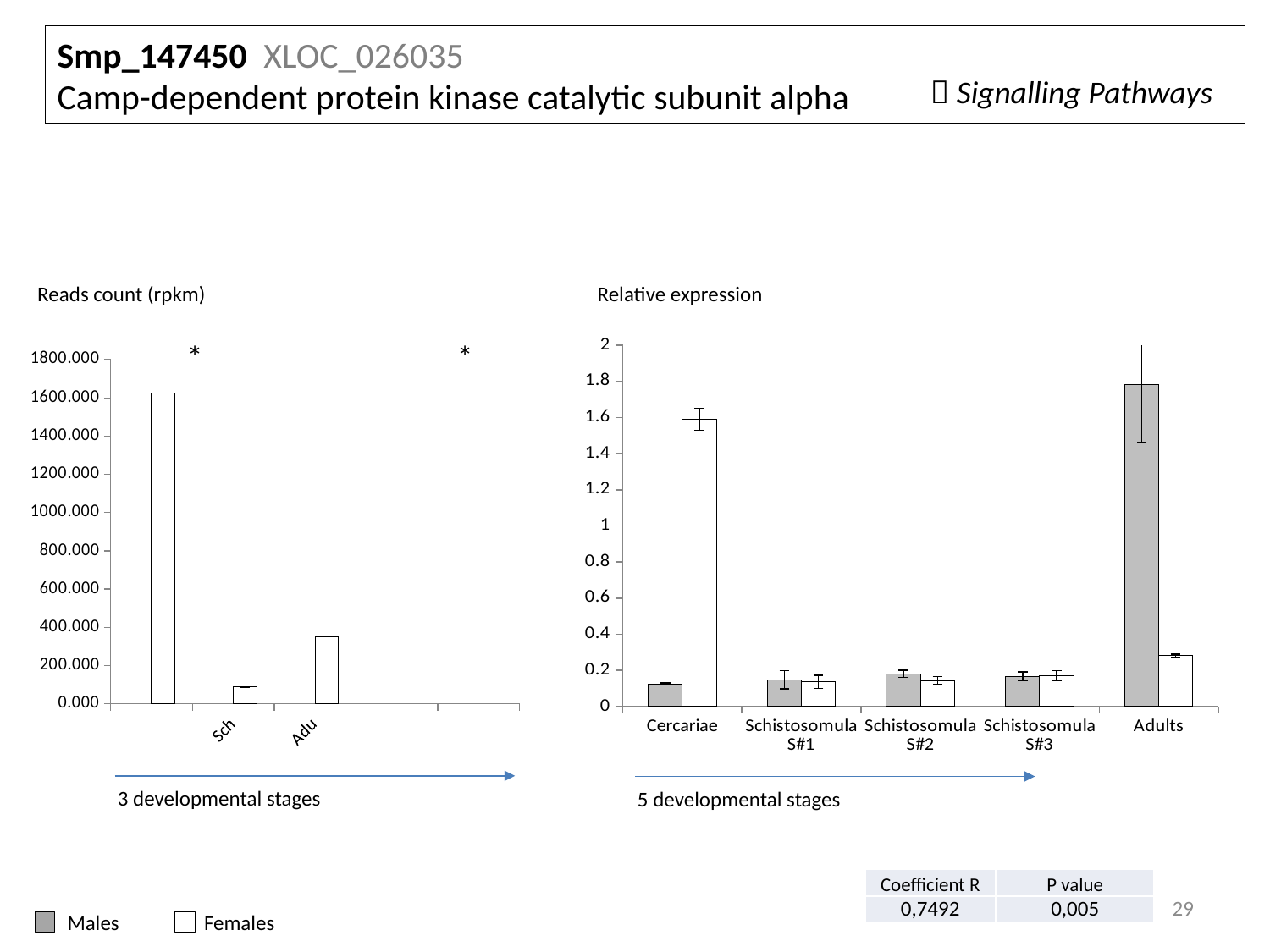

Smp_147450 XLOC_026035
Camp-dependent protein kinase catalytic subunit alpha
 Signalling Pathways
Relative expression
Reads count (rpkm)
*
*
### Chart
| Category | Males | Females |
|---|---|---|
| Cercariae | 0.12490814970532485 | 1.5896825222484288 |
| Schistosomula S#1 | 0.14770662665160836 | 0.13657342008971285 |
| Schistosomula S#2 | 0.18088225393863575 | 0.14378025657093618 |
| Schistosomula S#3 | 0.16642965122346448 | 0.17044905858969295 |
| Adults | 1.7809556436933187 | 0.2805871518639642 |
### Chart
| Category | Males | Females |
|---|---|---|
| Cercariae | 147.265415837164 | 1624.11287557226 |
| Schistosomula S#2 | 71.7395756364161 | 86.7104525316026 |
| Adults | 151.062157612176 | 351.94413412615 |3 developmental stages
5 developmental stages
| Coefficient R | P value |
| --- | --- |
| 0,7492 | 0,005 |
29
Males 	 Females

## Slide 30
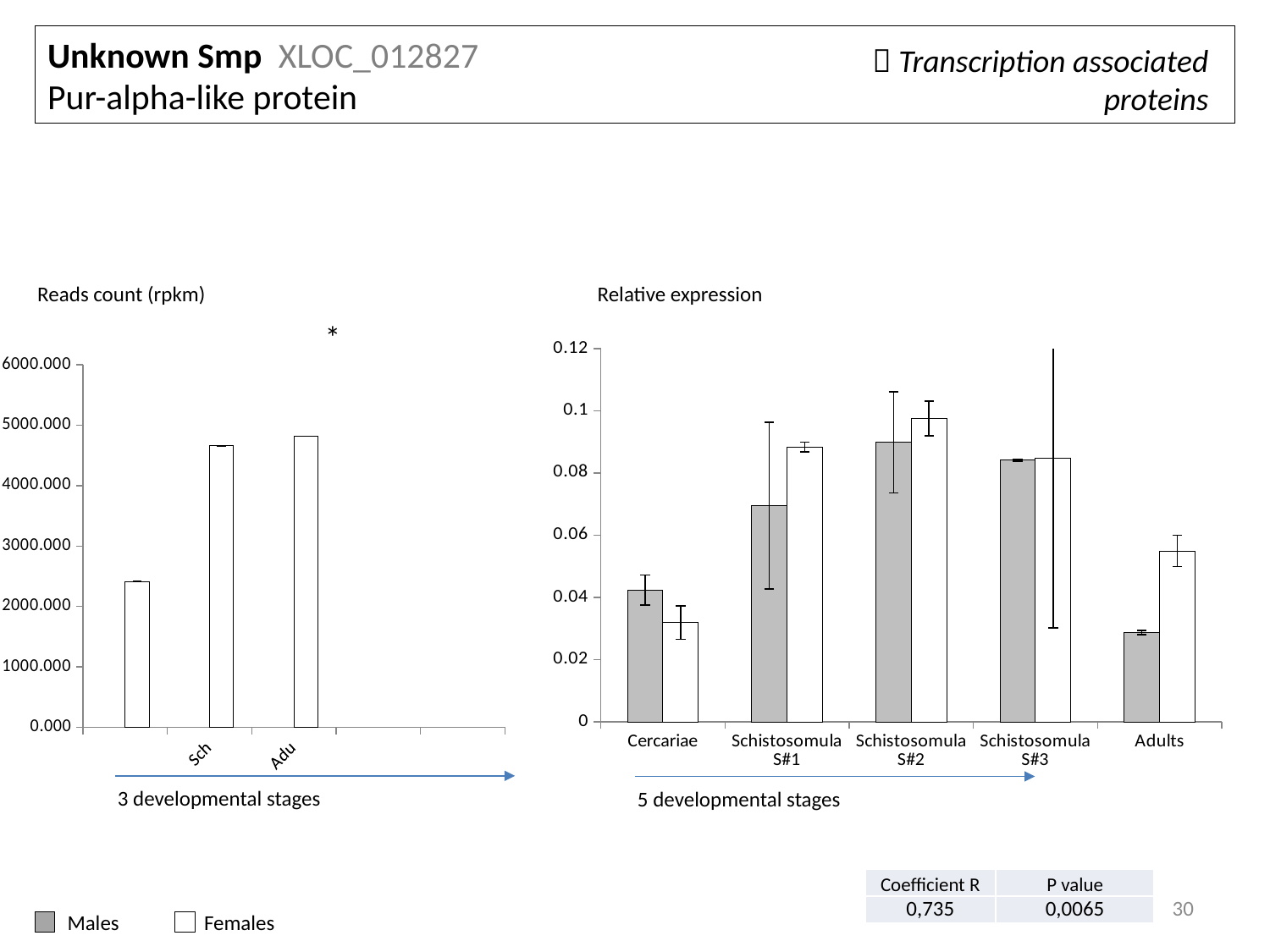

Unknown Smp XLOC_012827
Pur-alpha-like protein
 Transcription associated proteins
Relative expression
Reads count (rpkm)
*
### Chart
| Category | Males | Females |
|---|---|---|
| Cercariae | 0.04238056139607245 | 0.031919053129263505 |
| Schistosomula S#1 | 0.06950666585211485 | 0.08833020059792751 |
| Schistosomula S#2 | 0.08983051633725345 | 0.09752468675631329 |
| Schistosomula S#3 | 0.08412005404231637 | 0.0848652642753862 |
| Adults | 0.02865397504087494 | 0.05494931313006242 |
### Chart
| Category | Males | Females |
|---|---|---|
| Cercariae | 4014.55008352571 | 2414.8452746033 |
| Schistosomula S#2 | 12919.5048880543 | 4658.00507953394 |
| Adults | 4039.20168420561 | 4820.27261756061 |3 developmental stages
5 developmental stages
| Coefficient R | P value |
| --- | --- |
| 0,735 | 0,0065 |
30
Males 	 Females

## Slide 31
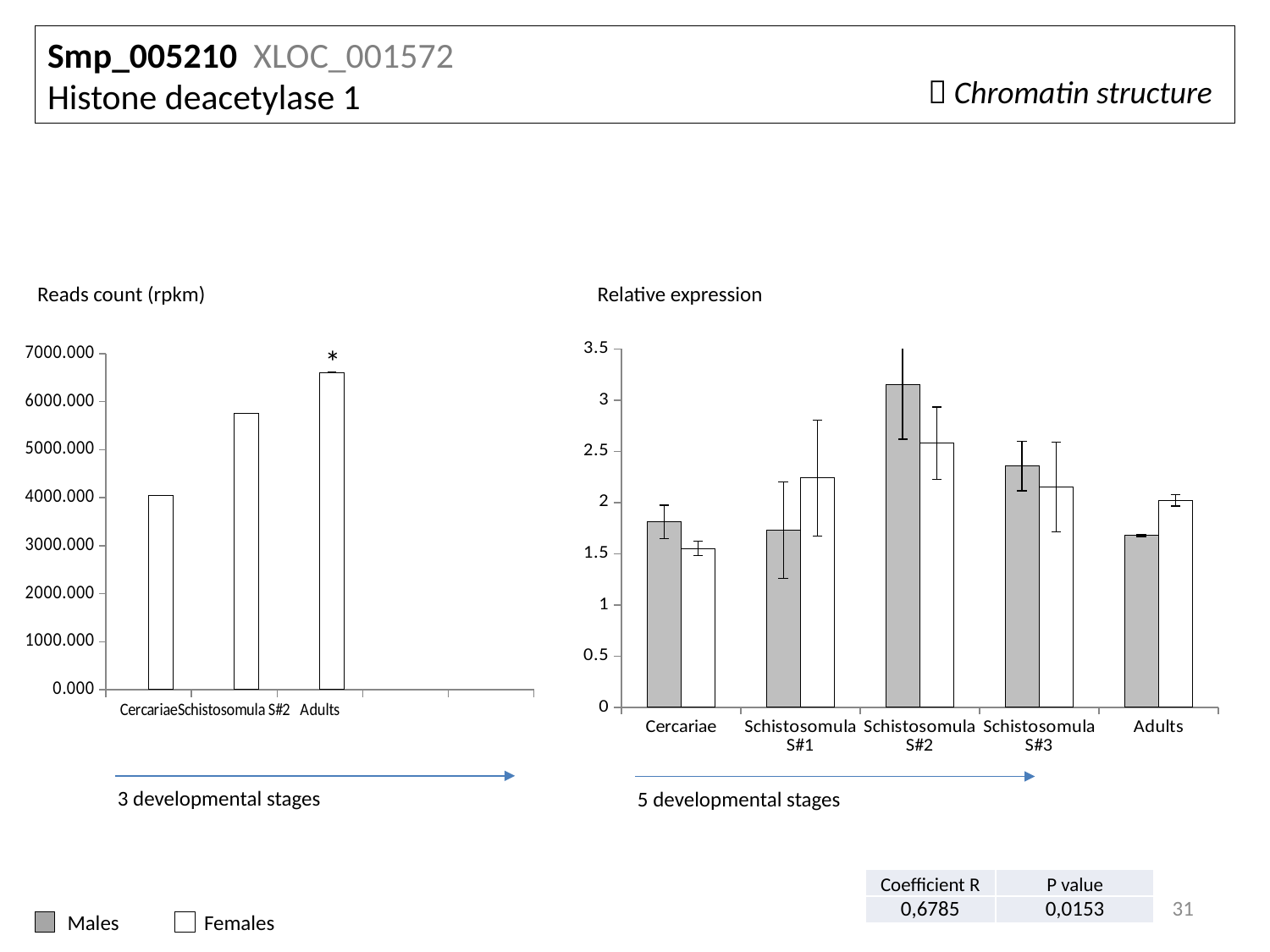

Smp_005210 XLOC_001572
Histone deacetylase 1
 Chromatin structure
Relative expression
Reads count (rpkm)
*
### Chart
| Category | Males | Females |
|---|---|---|
| Cercariae | 1.8099693675961426 | 1.5520326801464568 |
| Schistosomula S#1 | 1.731572439983712 | 2.240602983521601 |
| Schistosomula S#2 | 3.152615253916214 | 2.580865138399325 |
| Schistosomula S#3 | 2.355834971137119 | 2.153749022893139 |
| Adults | 1.6782926270350855 | 2.022594705827962 |
### Chart
| Category | Males | Females |
|---|---|---|
| Cercariae | 5350.21333212318 | 4047.90439388784 |
| Schistosomula S#2 | 13770.5010125993 | 5751.64344029965 |
| Adults | 5820.588466687 | 6612.01614637922 |3 developmental stages
5 developmental stages
| Coefficient R | P value |
| --- | --- |
| 0,6785 | 0,0153 |
31
Males 	 Females

## Slide 32
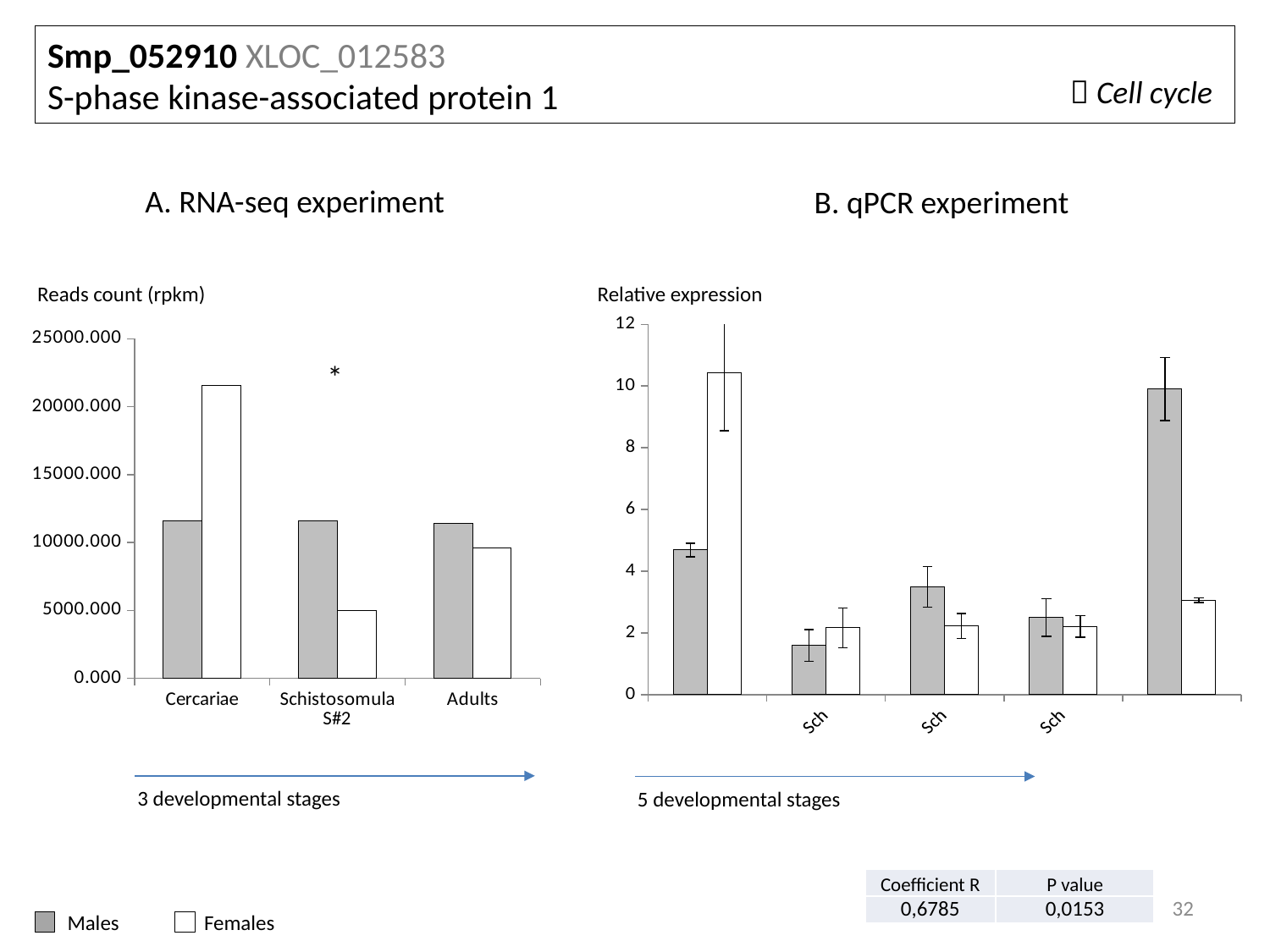

Smp_052910 XLOC_012583
S-phase kinase-associated protein 1
 Cell cycle
A. RNA-seq experiment
B. qPCR experiment
Relative expression
Reads count (rpkm)
### Chart
| Category | Males | Females |
|---|---|---|
| Cercariae | 4.688631383889348 | 10.418579766418212 |
| Schistosomula S#1 | 1.5975470027846224 | 2.1631656448858876 |
| Schistosomula S#2 | 3.4926251923088154 | 2.217924644044385 |
| Schistosomula S#3 | 2.4981354756867833 | 2.2070740456159967 |
| Adults | 9.898322651109547 | 3.0547968244915573 |
### Chart
| Category | Males | Females |
|---|---|---|
| Cercariae | 11572.406125026582 | 21567.8856389943 |
| Schistosomula S#2 | 11622.8908743443 | 5007.70633222509 |
| Adults | 11385.8854760457 | 9588.972536405081 |*
3 developmental stages
5 developmental stages
| Coefficient R | P value |
| --- | --- |
| 0,6785 | 0,0153 |
32
Males 	 Females

## Slide 33
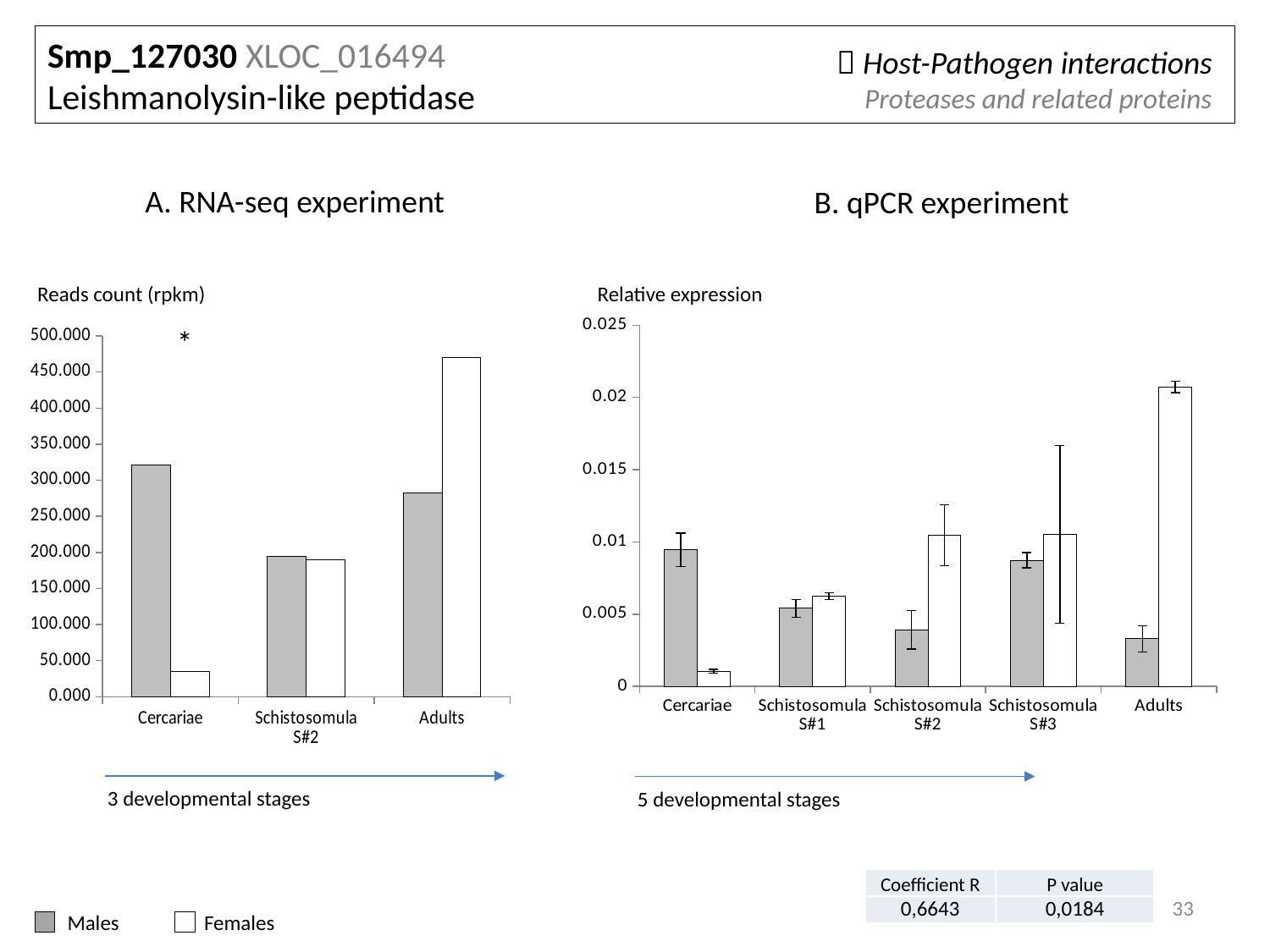

Smp_127030 XLOC_016494
Leishmanolysin-like peptidase
 Host-Pathogen interactions
Proteases and related proteins
A. RNA-seq experiment
B. qPCR experiment
Relative expression
Reads count (rpkm)
*
### Chart
| Category | Males | Females |
|---|---|---|
| Cercariae | 0.009457822807967885 | 0.0010579834462204714 |
| Schistosomula S#1 | 0.0053973367832126935 | 0.006236186677300935 |
| Schistosomula S#2 | 0.0039258101897552584 | 0.010469570714160078 |
| Schistosomula S#3 | 0.008736737053038256 | 0.010534322700589927 |
| Adults | 0.003293783149005313 | 0.020737745090630817 |
### Chart
| Category | Males | Females |
|---|---|---|
| Cercariae | 321.17977221153404 | 35.358662537710835 |
| Schistosomula S#2 | 194.4563274188603 | 190.147498143501 |
| Adults | 282.29850535649337 | 469.5227485570867 |3 developmental stages
5 developmental stages
| Coefficient R | P value |
| --- | --- |
| 0,6643 | 0,0184 |
33
Males 	 Females

## Slide 34
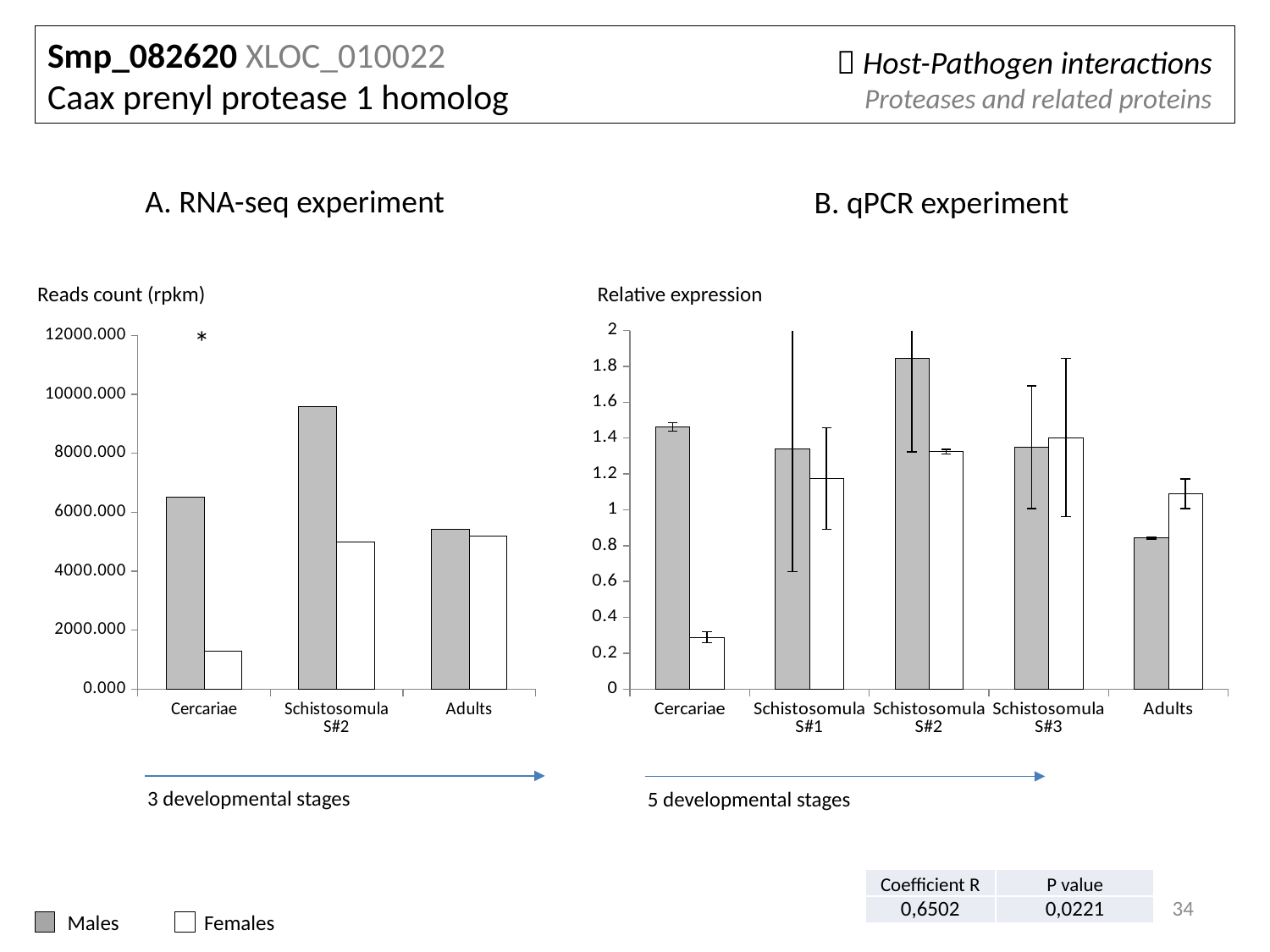

Smp_082620 XLOC_010022
Caax prenyl protease 1 homolog
 Host-Pathogen interactions
Proteases and related proteins
A. RNA-seq experiment
B. qPCR experiment
Relative expression
Reads count (rpkm)
*
### Chart
| Category | Males | Females |
|---|---|---|
| Cercariae | 1.4617977919060832 | 0.28972581462083513 |
| Schistosomula S#1 | 1.3407023919457262 | 1.1745160477904868 |
| Schistosomula S#2 | 1.8441386703343523 | 1.324944167309783 |
| Schistosomula S#3 | 1.3486060549181236 | 1.403458398779887 |
| Adults | 0.8435240628354337 | 1.0903683218130646 |
### Chart
| Category | Males | Females |
|---|---|---|
| Cercariae | 6513.864926663335 | 1281.8104639174398 |
| Schistosomula S#2 | 9569.36419581292 | 4991.584605183683 |
| Adults | 5426.68895866407 | 5184.27821302786 |3 developmental stages
5 developmental stages
| Coefficient R | P value |
| --- | --- |
| 0,6502 | 0,0221 |
34
Males 	 Females

## Slide 35
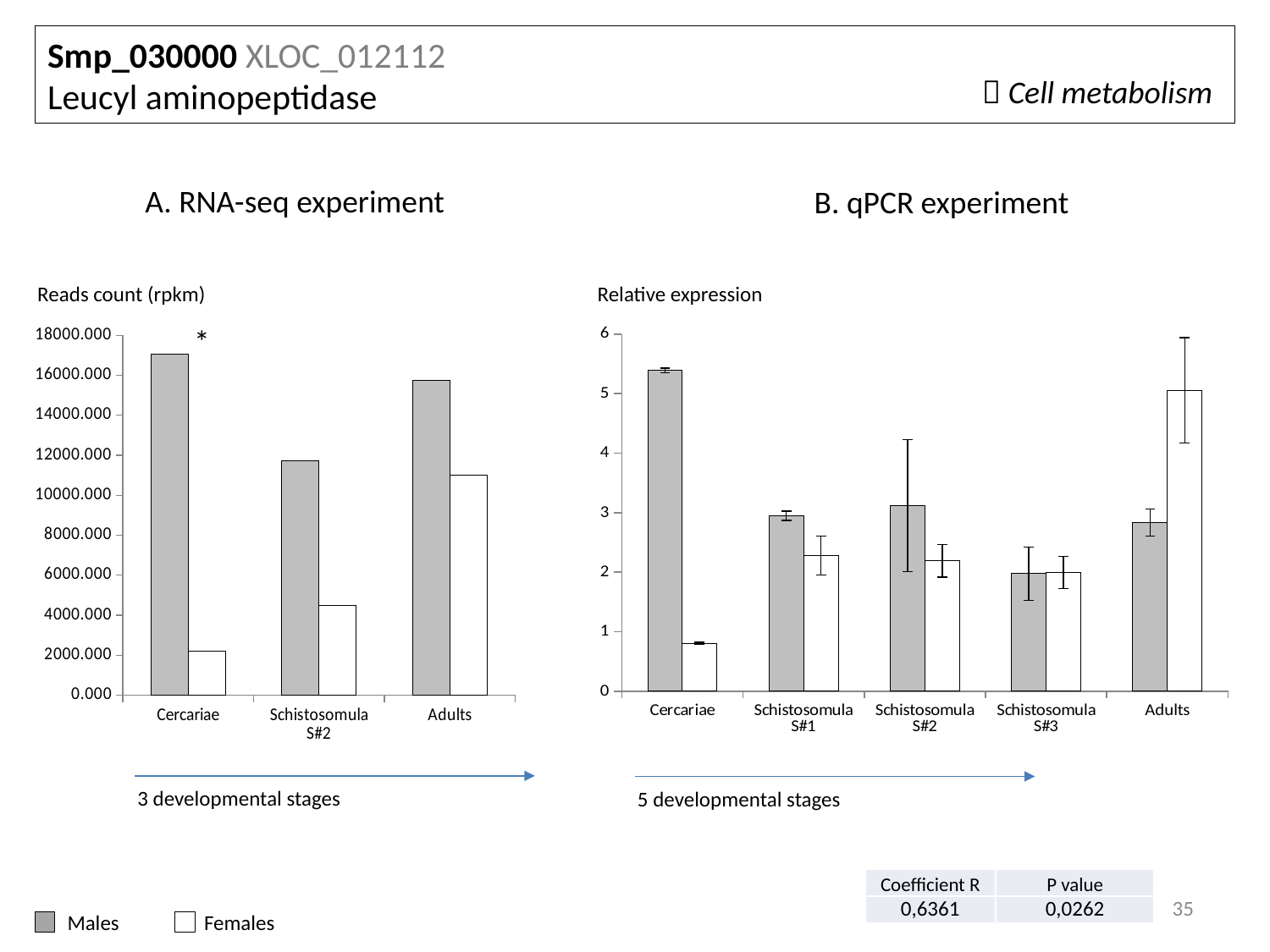

Smp_030000 XLOC_012112
Leucyl aminopeptidase
 Cell metabolism
A. RNA-seq experiment
B. qPCR experiment
Relative expression
Reads count (rpkm)
*
### Chart
| Category | Males | Females |
|---|---|---|
| Cercariae | 17032.4808303779 | 2209.6529833282098 |
| Schistosomula S#2 | 11743.983063194502 | 4468.42138062001 |
| Adults | 15751.446303158 | 11010.485178583482 |
### Chart
| Category | Males | Females |
|---|---|---|
| Cercariae | 5.389298129822045 | 0.8054647029760471 |
| Schistosomula S#1 | 2.948077673271129 | 2.280037426487584 |
| Schistosomula S#2 | 3.119100046424359 | 2.1906713294210336 |
| Schistosomula S#3 | 1.9749999597935421 | 1.9958596471077397 |
| Adults | 2.8364852623529373 | 5.056083137819265 |3 developmental stages
5 developmental stages
| Coefficient R | P value |
| --- | --- |
| 0,6361 | 0,0262 |
35
Males 	 Females

## Slide 36
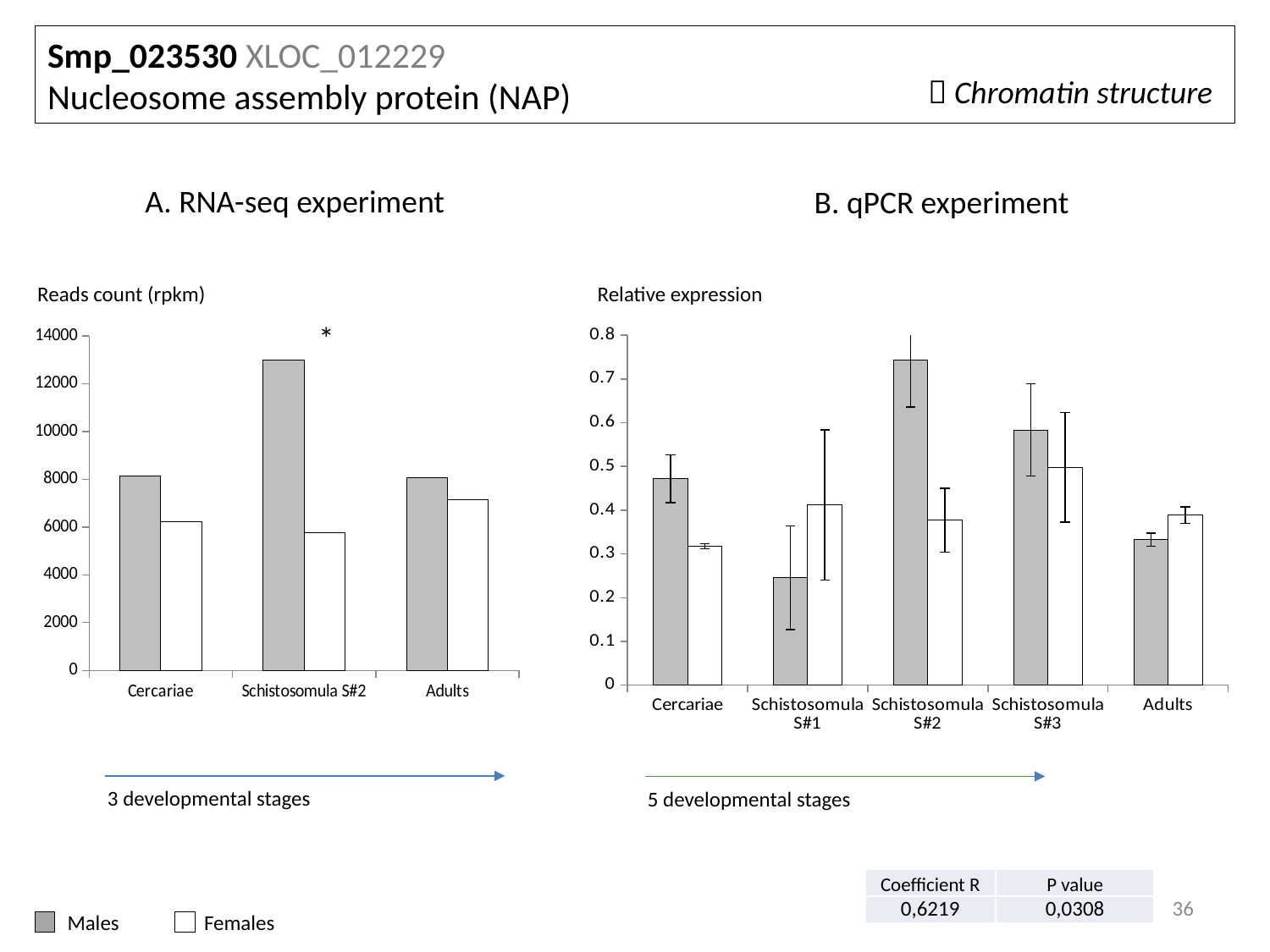

Smp_023530 XLOC_012229
Nucleosome assembly protein (NAP)
 Chromatin structure
A. RNA-seq experiment
B. qPCR experiment
Relative expression
Reads count (rpkm)
*
### Chart
| Category | Males | Females |
|---|---|---|
| Cercariae | 0.4721556468160939 | 0.3176345852900205 |
| Schistosomula S#1 | 0.2455946597330924 | 0.4121802035103983 |
| Schistosomula S#2 | 0.7434888088115439 | 0.37678876346197904 |
| Schistosomula S#3 | 0.5834019250880847 | 0.49786517536034114 |
| Adults | 0.33237968789845085 | 0.3888621517039451 |
### Chart
| Category | Males | Females |
|---|---|---|
| Cercariae | 8144.376517374169 | 6225.53505462948 |
| Schistosomula S#2 | 13003.5952719459 | 5772.659228511866 |
| Adults | 8063.5943475100385 | 7157.483147116361 |3 developmental stages
5 developmental stages
| Coefficient R | P value |
| --- | --- |
| 0,6219 | 0,0308 |
36
Males 	 Females

## Slide 37
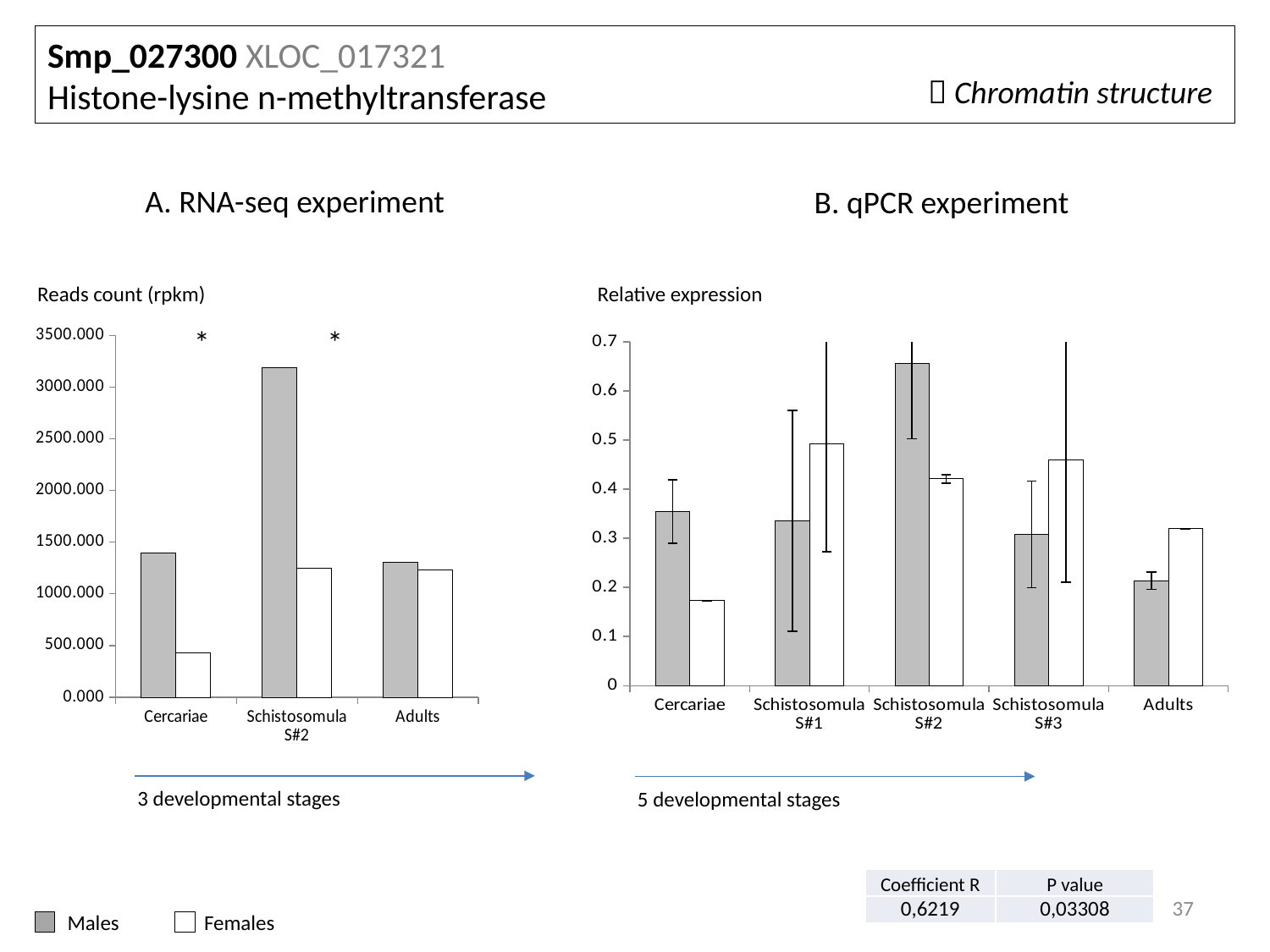

Smp_027300 XLOC_017321
Histone-lysine n-methyltransferase
 Chromatin structure
A. RNA-seq experiment
B. qPCR experiment
Relative expression
Reads count (rpkm)
*
*
### Chart
| Category | Males | Females |
|---|---|---|
| Cercariae | 1394.27048660258 | 429.38156984441895 |
| Schistosomula S#2 | 3190.13783431767 | 1247.33832543966 |
| Adults | 1307.3363601511 | 1227.20219818283 |
### Chart
| Category | Males | Females |
|---|---|---|
| Cercariae | 0.35462782161053363 | 0.17315862131000698 |
| Schistosomula S#1 | 0.33523580621415267 | 0.4919217114439916 |
| Schistosomula S#2 | 0.6553726244670611 | 0.42103806401328076 |
| Schistosomula S#3 | 0.30816400262247456 | 0.4598373238338367 |
| Adults | 0.21342581001676644 | 0.3193362601480001 |3 developmental stages
5 developmental stages
| Coefficient R | P value |
| --- | --- |
| 0,6219 | 0,03308 |
37
Males 	 Females

## Slide 38
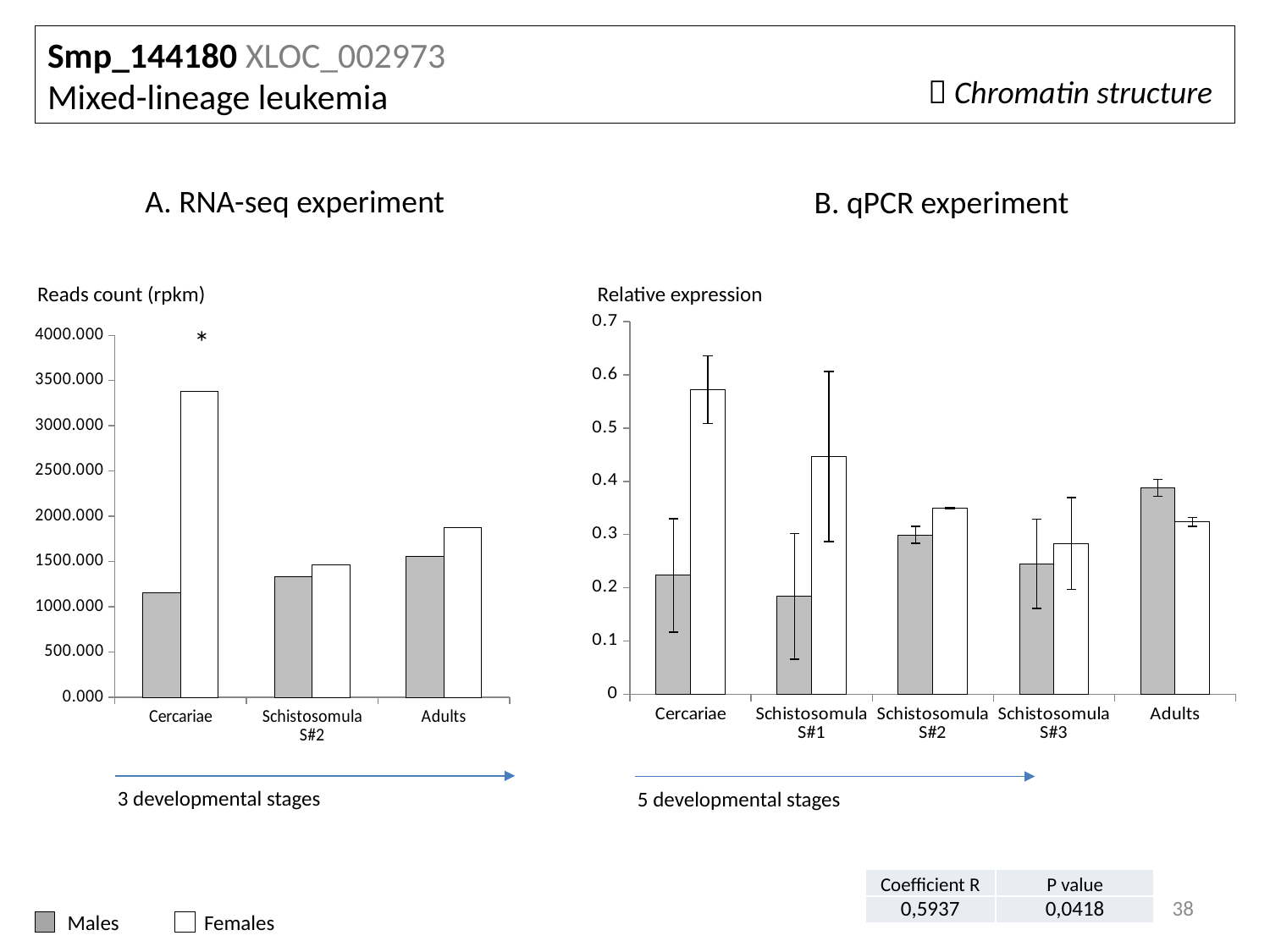

Smp_144180 XLOC_002973
Mixed-lineage leukemia
 Chromatin structure
A. RNA-seq experiment
B. qPCR experiment
Relative expression
Reads count (rpkm)
### Chart
| Category | Males | Females |
|---|---|---|
| Cercariae | 0.22330962046830385 | 0.5720537810407265 |
| Schistosomula S#1 | 0.18393066960598195 | 0.44676995981784084 |
| Schistosomula S#2 | 0.2995403990535398 | 0.3494861281768537 |
| Schistosomula S#3 | 0.24511857422687744 | 0.28309114109312483 |
| Adults | 0.3877353295546591 | 0.32390132871869404 |*
### Chart
| Category | Males | Females |
|---|---|---|
| Cercariae | 1153.1083609733 | 3380.44487580594 |
| Schistosomula S#2 | 1331.33863956676 | 1458.8966341168098 |
| Adults | 1558.6871394572997 | 1877.3013786263698 |3 developmental stages
5 developmental stages
| Coefficient R | P value |
| --- | --- |
| 0,5937 | 0,0418 |
38
Males 	 Females

## Slide 39
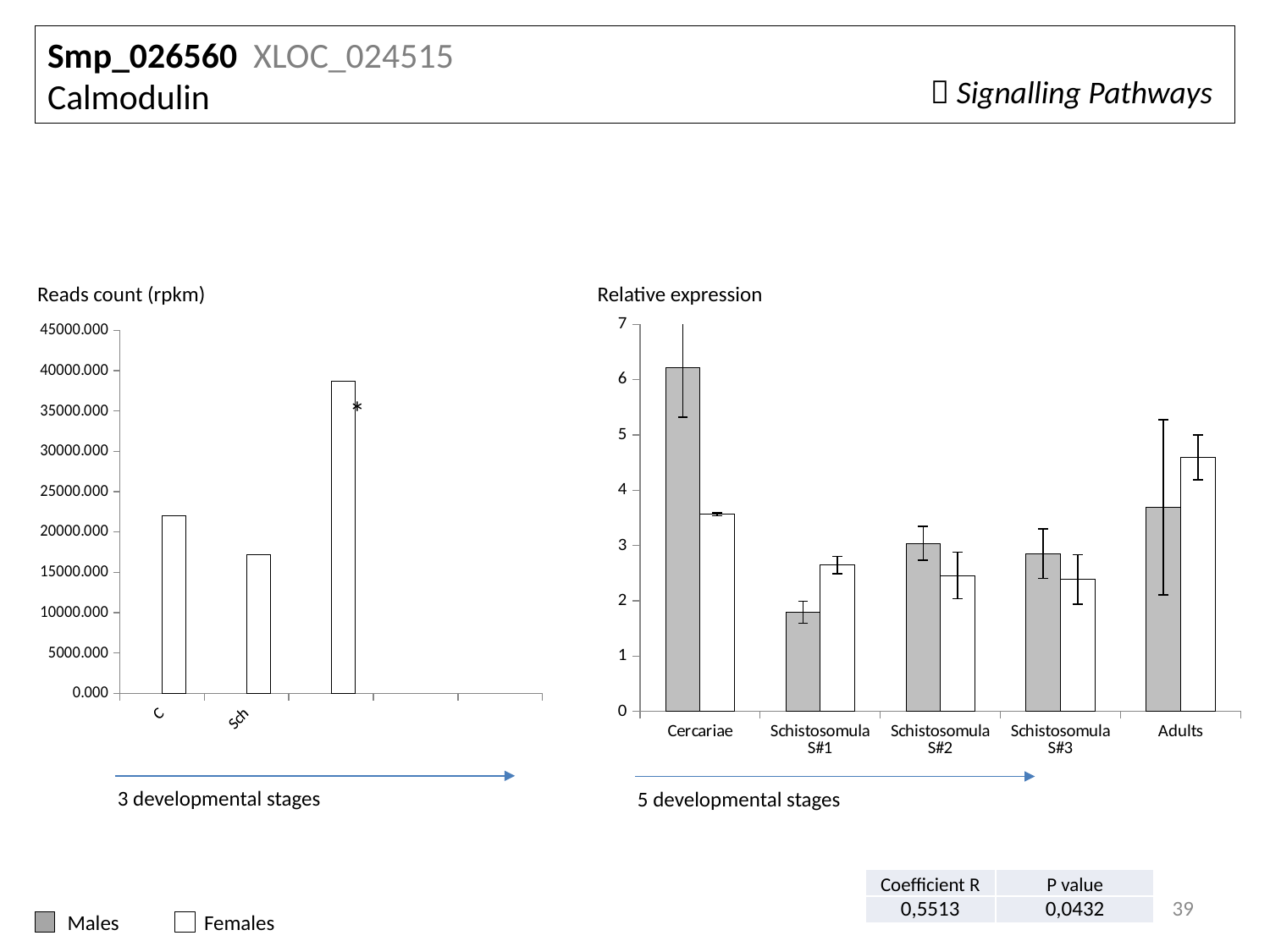

Smp_026560 XLOC_024515
Calmodulin
 Signalling Pathways
Relative expression
Reads count (rpkm)
### Chart
| Category | Males | Females |
|---|---|---|
| Cercariae | 6.212592857218697 | 3.562041676920808 |
| Schistosomula S#1 | 1.7938388070412823 | 2.6463109541230754 |
| Schistosomula S#2 | 3.039880475531638 | 2.457452004331322 |
| Schistosomula S#3 | 2.8513494283186827 | 2.3865712013893114 |
| Adults | 3.6902702708393456 | 4.590957391621549 |
### Chart
| Category | Males | Females |
|---|---|---|
| Cercariae | 56524.2986872349 | 22033.3119168266 |
| Schistosomula S#2 | 37838.7453110254 | 17167.9914489287 |
| Adults | 58424.1430156323 | 38666.4463993655 |*
3 developmental stages
5 developmental stages
| Coefficient R | P value |
| --- | --- |
| 0,5513 | 0,0432 |
39
Males 	 Females

## Slide 40
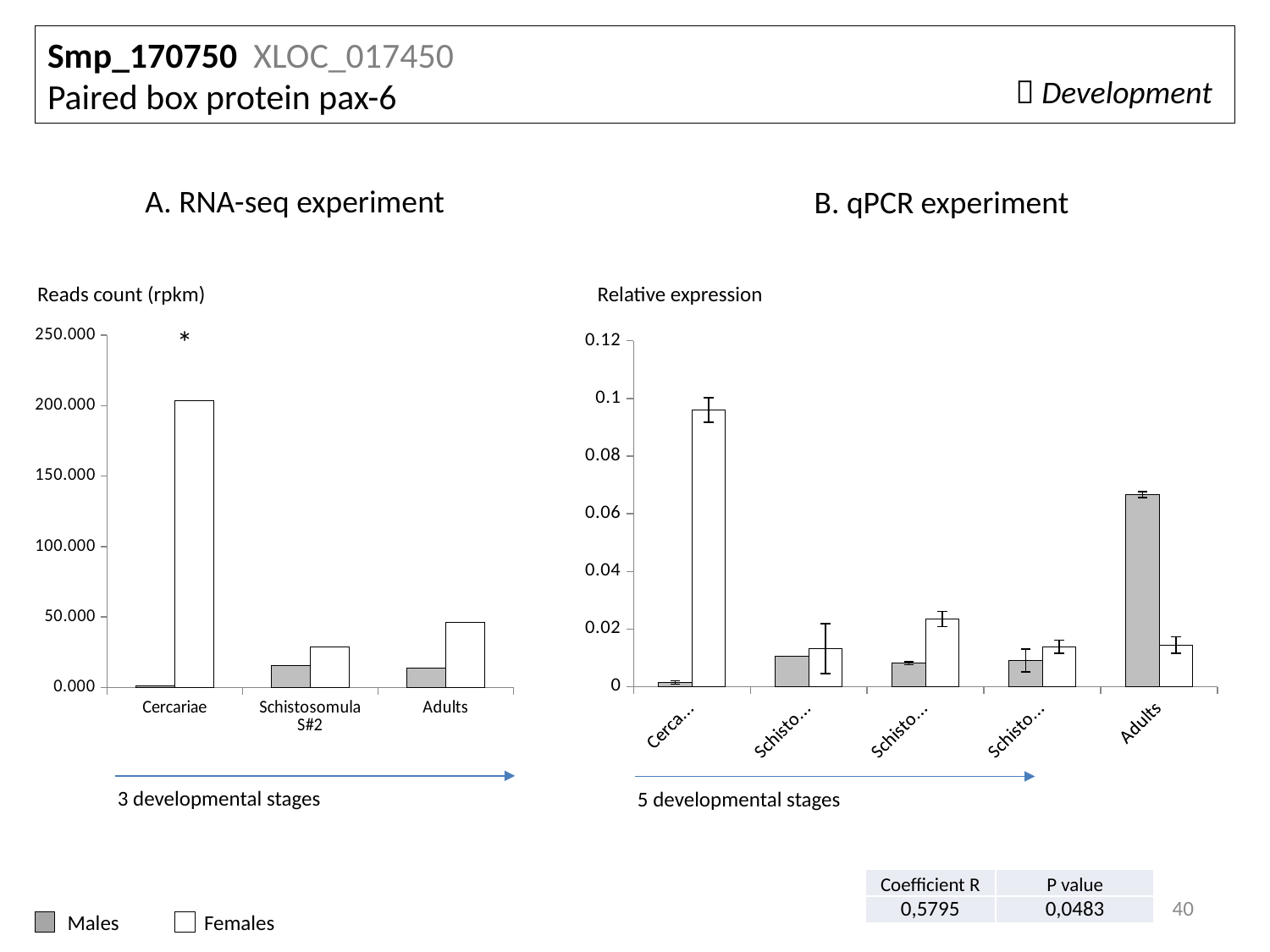

Smp_170750 XLOC_017450
Paired box protein pax-6
 Development
### Chart
| Category | Males | Females |
|---|---|---|
| Cercariae | 0.0014759630394028968 | 0.09599471887919866 |
| Schistosomula S#1 | 0.01059067352116443 | 0.013245970456836748 |
| Schistosomula S#2 | 0.008157840709857039 | 0.023469580663463725 |
| Schistosomula S#3 | 0.009084967849444247 | 0.013924328515274684 |
| Adults | 0.06662268770425848 | 0.01449354042972942 |A. RNA-seq experiment
B. qPCR experiment
Relative expression
Reads count (rpkm)
*
### Chart
| Category | Males | Females |
|---|---|---|
| Cercariae | 1.438930153204048 | 203.3994237260687 |
| Schistosomula S#2 | 15.523024670776 | 28.727479606778587 |
| Adults | 13.445465669528318 | 46.04870599742779 |3 developmental stages
5 developmental stages
| Coefficient R | P value |
| --- | --- |
| 0,5795 | 0,0483 |
40
Males 	 Females

## Slide 41
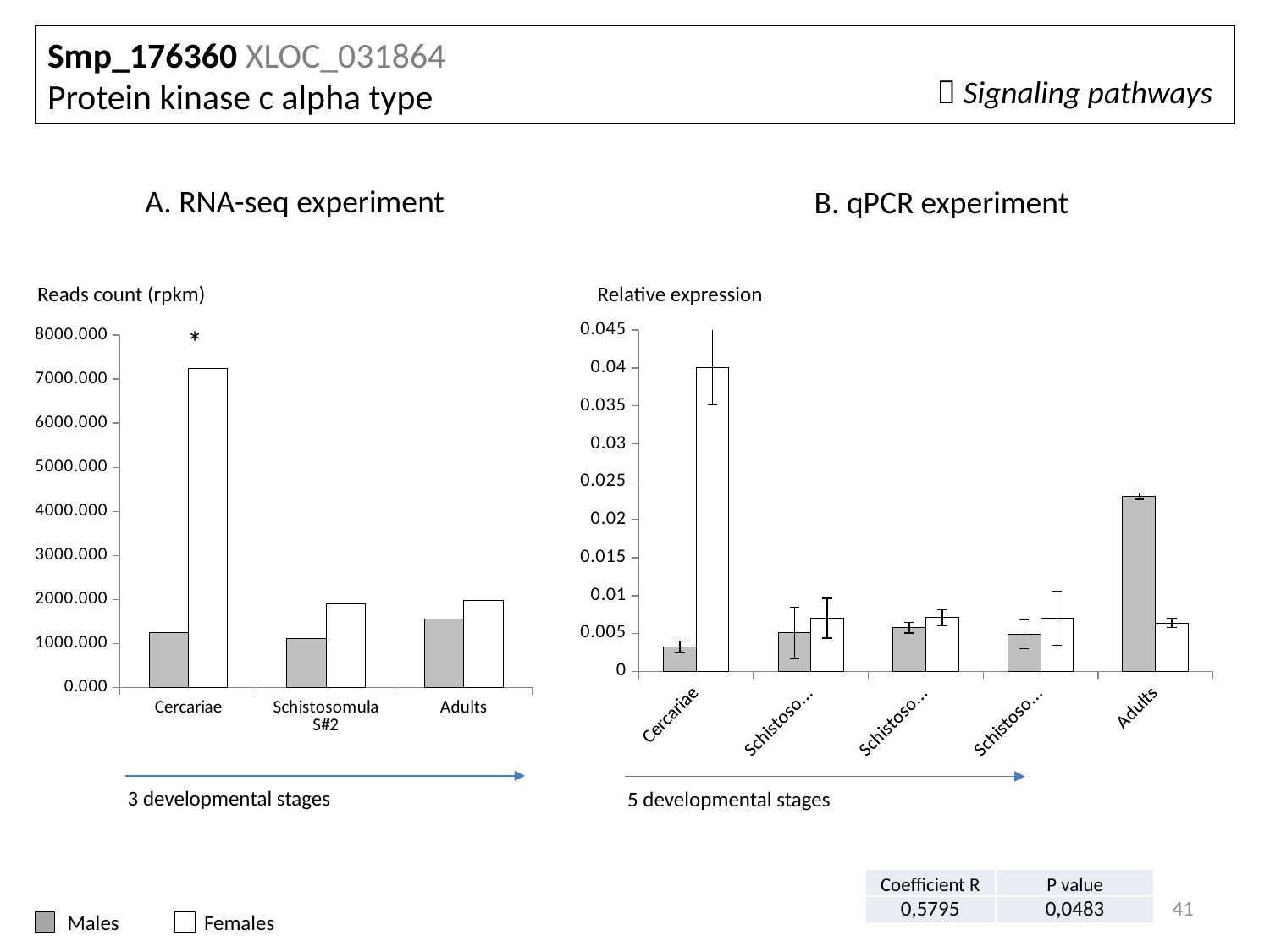

Smp_176360 XLOC_031864
Protein kinase c alpha type
 Signaling pathways
A. RNA-seq experiment
B. qPCR experiment
### Chart
| Category | Males | Females |
|---|---|---|
| Cercariae | 0.003229115069685917 | 0.04007322027623205 |
| Schistosomula S#1 | 0.005074360308391008 | 0.0070098844486563144 |
| Schistosomula S#2 | 0.005759267145195281 | 0.007081772346402537 |
| Schistosomula S#3 | 0.004905459631933094 | 0.00701093215455453 |
| Adults | 0.02311061891761892 | 0.006374251723399355 |Relative expression
Reads count (rpkm)
*
### Chart
| Category | Males | Females |
|---|---|---|
| Cercariae | 1238.67680999399 | 7248.4435338882695 |
| Schistosomula S#2 | 1118.8511828268681 | 1903.0009701522201 |
| Adults | 1554.25123769022 | 1972.661855703591 |3 developmental stages
5 developmental stages
| Coefficient R | P value |
| --- | --- |
| 0,5795 | 0,0483 |
41
Males 	 Females

## Slide 42
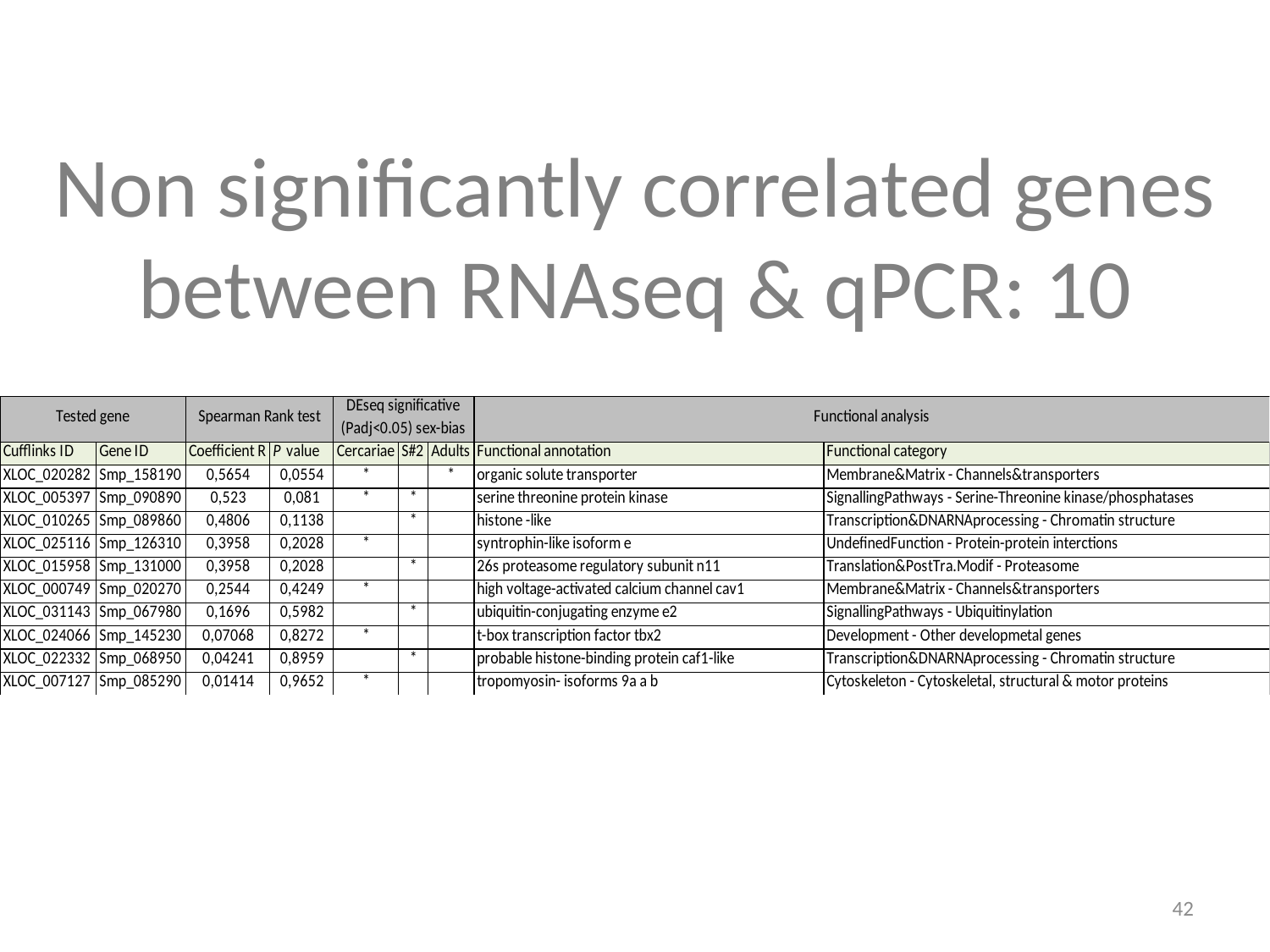

Non significantly correlated genes between RNAseq & qPCR: 10
42

## Slide 43
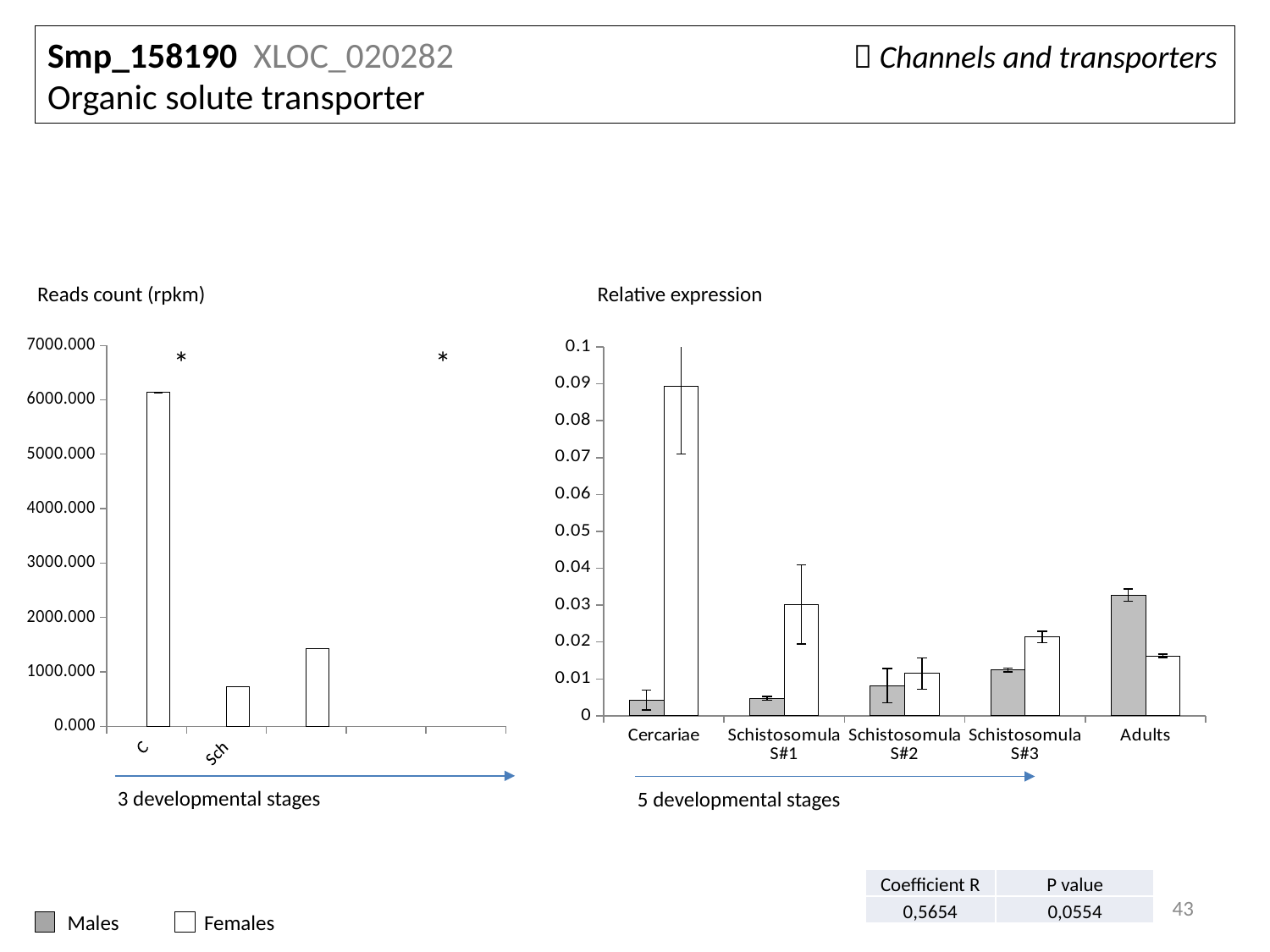

Smp_158190 XLOC_020282
Organic solute transporter
 Channels and transporters
Relative expression
Reads count (rpkm)
### Chart
| Category | Males | Females |
|---|---|---|
| Cercariae | 525.429494028534 | 6131.81655274234 |
| Schistosomula S#2 | 968.277894265741 | 729.225426537418 |
| Adults | 691.420543373174 | 1428.64517600666 |
### Chart
| Category | Males | Females |
|---|---|---|
| Cercariae | 0.004255707928822615 | 0.08940573389969703 |
| Schistosomula S#1 | 0.004740197797026897 | 0.03020273572818893 |
| Schistosomula S#2 | 0.008166577973744761 | 0.011448151739944368 |
| Schistosomula S#3 | 0.012364788550347321 | 0.021383946949194405 |
| Adults | 0.032721534201503824 | 0.0162474306447163 |*
*
3 developmental stages
5 developmental stages
| Coefficient R | P value |
| --- | --- |
| 0,5654 | 0,0554 |
43
Males 	 Females

## Slide 44
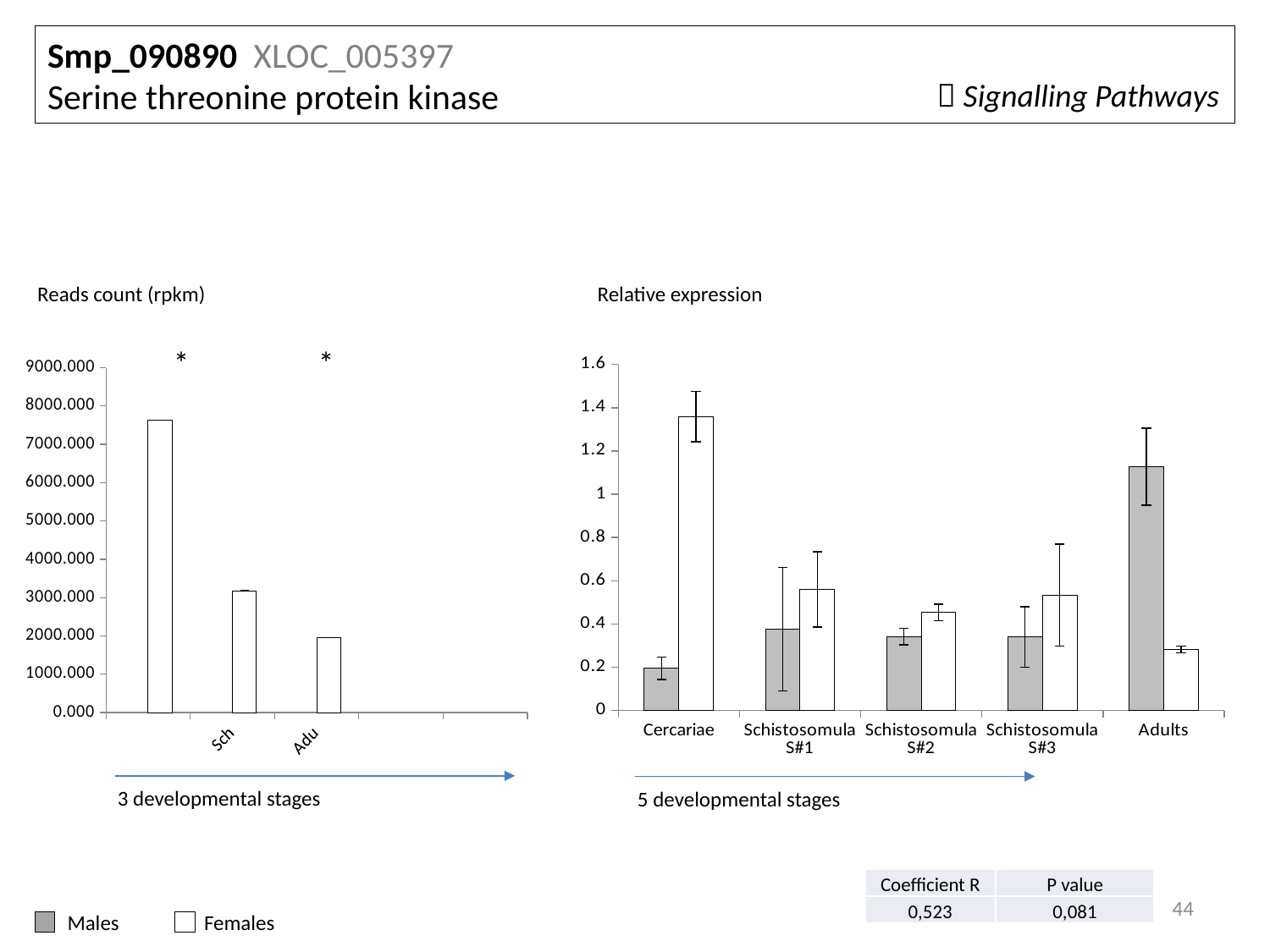

Smp_090890 XLOC_005397
Serine threonine protein kinase
 Signalling Pathways
Relative expression
Reads count (rpkm)
*
*
### Chart
| Category | Males | Females |
|---|---|---|
| Cercariae | 0.19510357655922328 | 1.3594432175825433 |
| Schistosomula S#1 | 0.3760429976421183 | 0.559610744871141 |
| Schistosomula S#2 | 0.342407015458843 | 0.45355264157985575 |
| Schistosomula S#3 | 0.3403288063138892 | 0.5342415429290897 |
| Adults | 1.1271030339438535 | 0.28279888515965046 |
### Chart
| Category | Males | Females |
|---|---|---|
| Cercariae | 1027.87209184279 | 7626.3898195323 |
| Schistosomula S#2 | 1447.5549477717 | 3176.10672234475 |
| Adults | 1527.53087421975 | 1956.03027640749 |3 developmental stages
5 developmental stages
| Coefficient R | P value |
| --- | --- |
| 0,523 | 0,081 |
44
Males 	 Females

## Slide 45
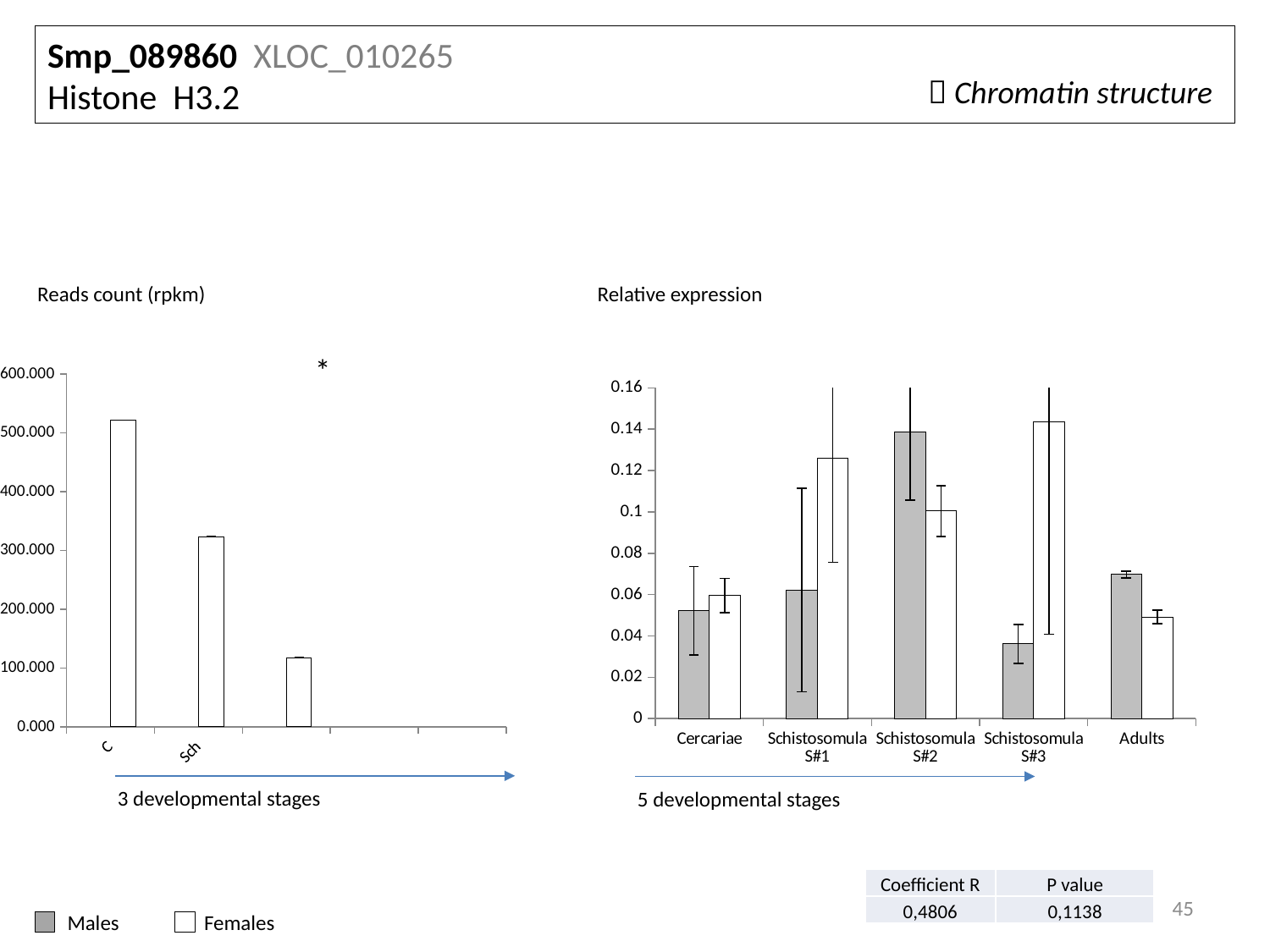

Smp_089860 XLOC_010265
Histone H3.2
 Chromatin structure
Relative expression
Reads count (rpkm)
*
### Chart
| Category | Males | Females |
|---|---|---|
| Cercariae | 0.05214656691954156 | 0.05950840758938469 |
| Schistosomula S#1 | 0.0622038989077425 | 0.12579164604180665 |
| Schistosomula S#2 | 0.13872484853532557 | 0.10047593758311088 |
| Schistosomula S#3 | 0.03612235546561782 | 0.1435469934506218 |
| Adults | 0.0697024274573125 | 0.04916543356396784 |
### Chart
| Category | Males | Females |
|---|---|---|
| Cercariae | 319.275900620957 | 522.254143886099 |
| Schistosomula S#2 | 858.035998154395 | 323.895318964348 |
| Adults | 234.597847715467 | 117.52401570728 |3 developmental stages
5 developmental stages
| Coefficient R | P value |
| --- | --- |
| 0,4806 | 0,1138 |
45
Males 	 Females

## Slide 46
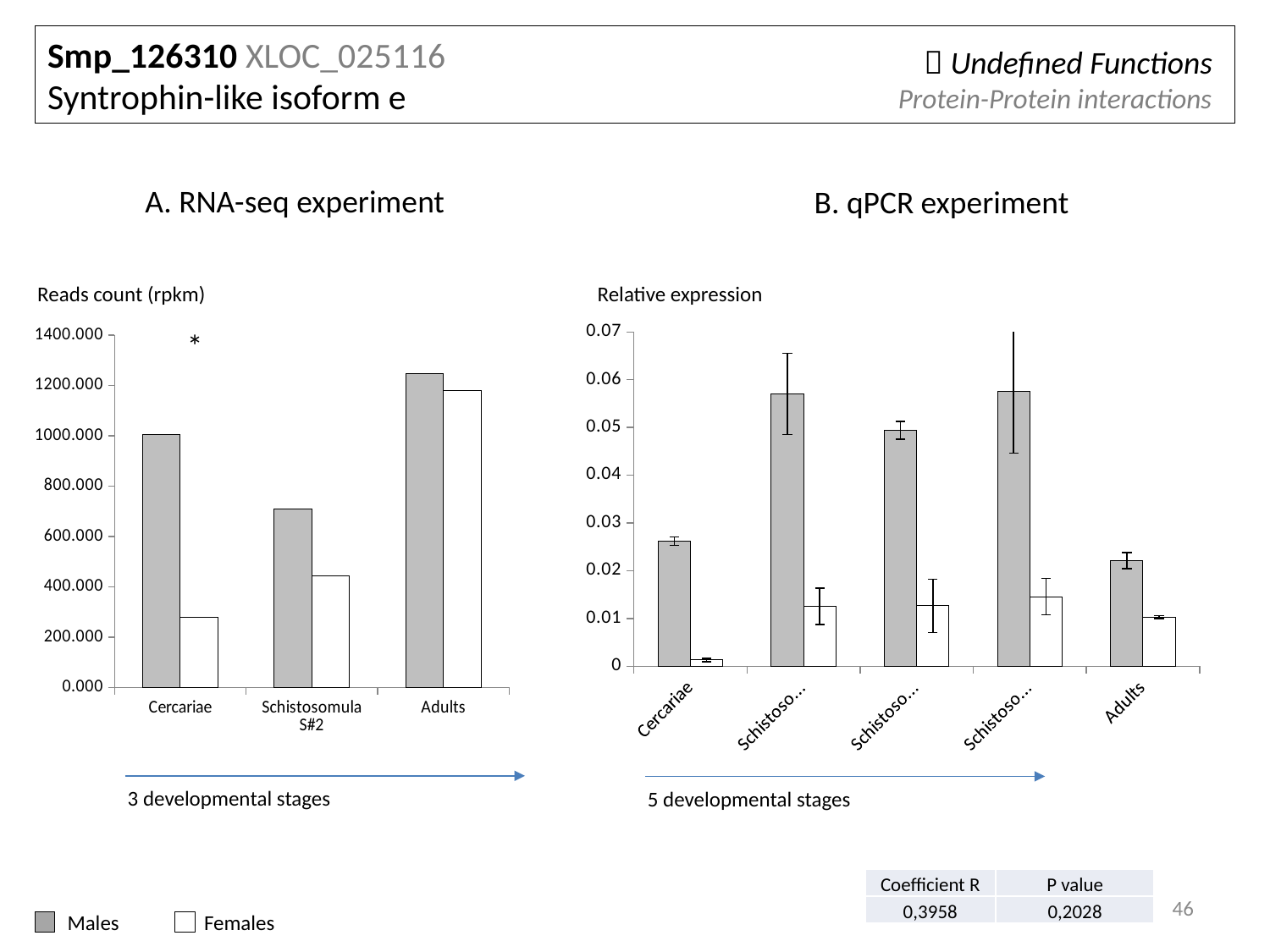

Smp_126310 XLOC_025116
Syntrophin-like isoform e
 Undefined Functions
Protein-Protein interactions
A. RNA-seq experiment
B. qPCR experiment
Relative expression
Reads count (rpkm)
### Chart
| Category | Males | Females |
|---|---|---|
| Cercariae | 0.026157369432162472 | 0.0013016642151127441 |
| Schistosomula S#1 | 0.05703605885749718 | 0.012539958685913889 |
| Schistosomula S#2 | 0.04939040718638118 | 0.012657595186976716 |
| Schistosomula S#3 | 0.0575406069825055 | 0.01457540518293162 |
| Adults | 0.022117003344525273 | 0.010264192857621608 |*
### Chart
| Category | Males | Females |
|---|---|---|
| Cercariae | 1005.7843104567513 | 279.2331382099372 |
| Schistosomula S#2 | 709.834982335742 | 445.208400722836 |
| Adults | 1248.26469356822 | 1181.8171064753 |3 developmental stages
5 developmental stages
| Coefficient R | P value |
| --- | --- |
| 0,3958 | 0,2028 |
46
Males 	 Females

## Slide 47
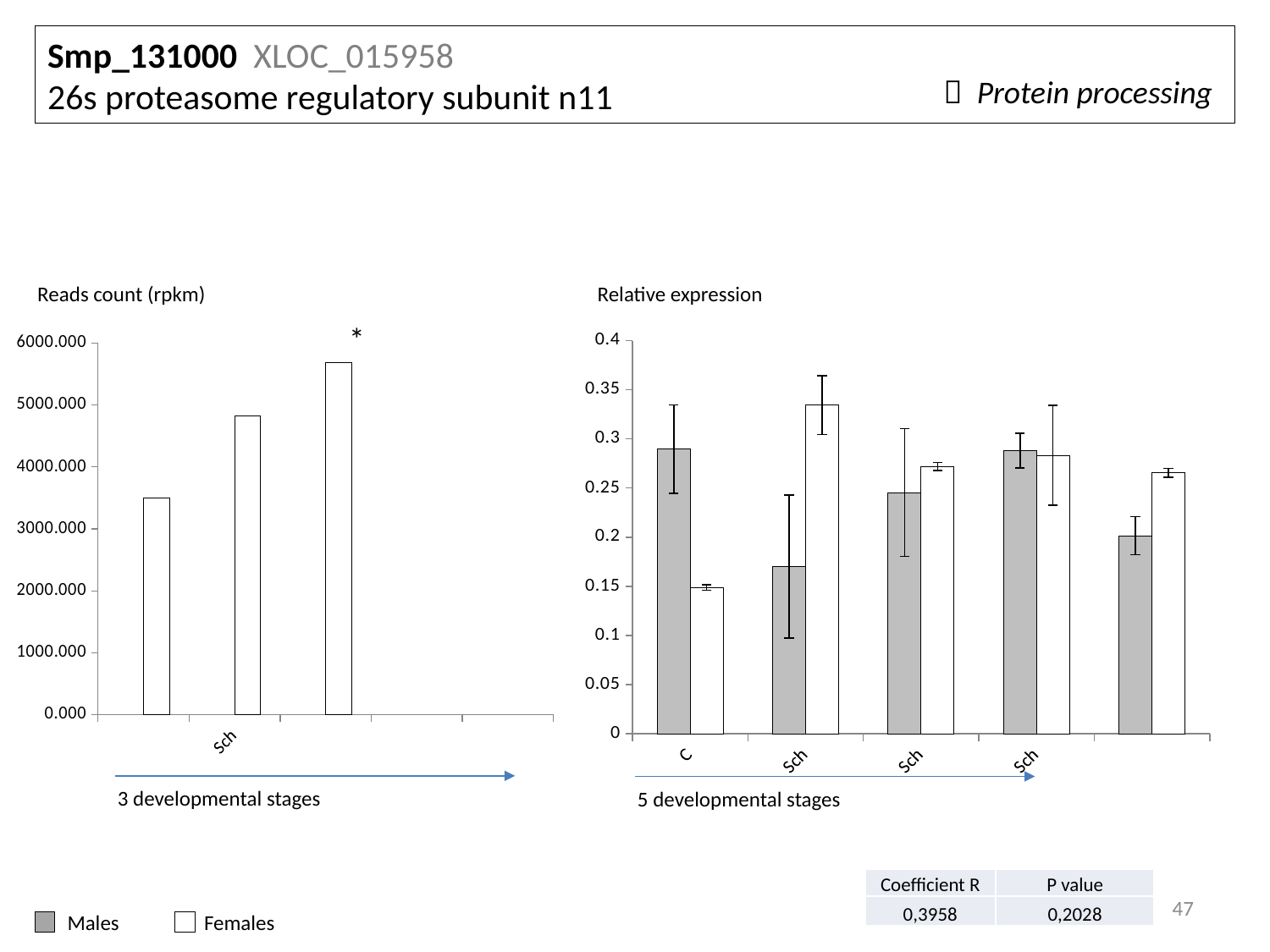

Smp_131000 XLOC_015958
26s proteasome regulatory subunit n11
 Protein processing
Relative expression
Reads count (rpkm)
*
### Chart
| Category | Males | Females |
|---|---|---|
| Cercariae | 0.28970016882440786 | 0.14869360194244885 |
| Schistosomula S#1 | 0.17017393558936036 | 0.33440741319631007 |
| Schistosomula S#2 | 0.24523100591252978 | 0.27175443284137857 |
| Schistosomula S#3 | 0.28793324379397933 | 0.28322758592789915 |
| Adults | 0.2014618961485256 | 0.26532964961008865 |
### Chart
| Category | Males | Females |
|---|---|---|
| Cercariae | 7068.42476652168 | 3499.01686805411 |
| Schistosomula S#2 | 11022.5824222303 | 4823.03448819045 |
| Adults | 6074.8666935965 | 5688.17126794112 |3 developmental stages
5 developmental stages
| Coefficient R | P value |
| --- | --- |
| 0,3958 | 0,2028 |
47
Males 	 Females

## Slide 48
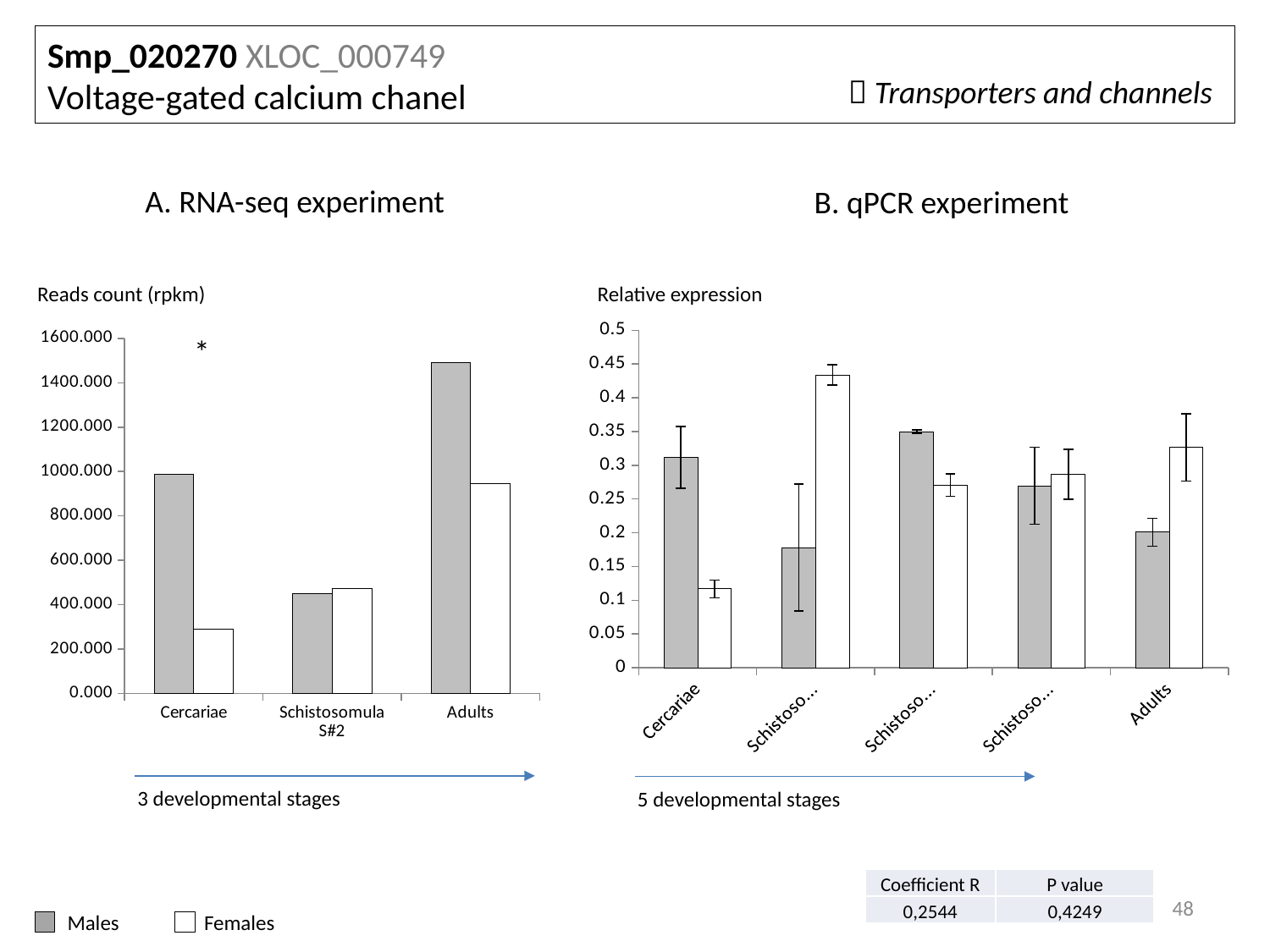

Smp_020270 XLOC_000749
Voltage-gated calcium chanel
 Transporters and channels
A. RNA-seq experiment
B. qPCR experiment
### Chart
| Category | Males | Females |
|---|---|---|
| Cercariae | 0.3117034573697016 | 0.11679124987604896 |
| Schistosomula S#1 | 0.1781545515499124 | 0.43367858303815165 |
| Schistosomula S#2 | 0.3499684286921759 | 0.2707594604436064 |
| Schistosomula S#3 | 0.2696957554603432 | 0.2868657416440679 |
| Adults | 0.2009026002124238 | 0.3265271937561167 |Relative expression
Reads count (rpkm)
*
### Chart
| Category | Males | Females |
|---|---|---|
| Cercariae | 987.184445035956 | 287.7676802998843 |
| Schistosomula S#2 | 451.474146088652 | 470.983606032504 |
| Adults | 1491.63189774835 | 945.325090061845 |3 developmental stages
5 developmental stages
| Coefficient R | P value |
| --- | --- |
| 0,2544 | 0,4249 |
48
Males 	 Females

## Slide 49
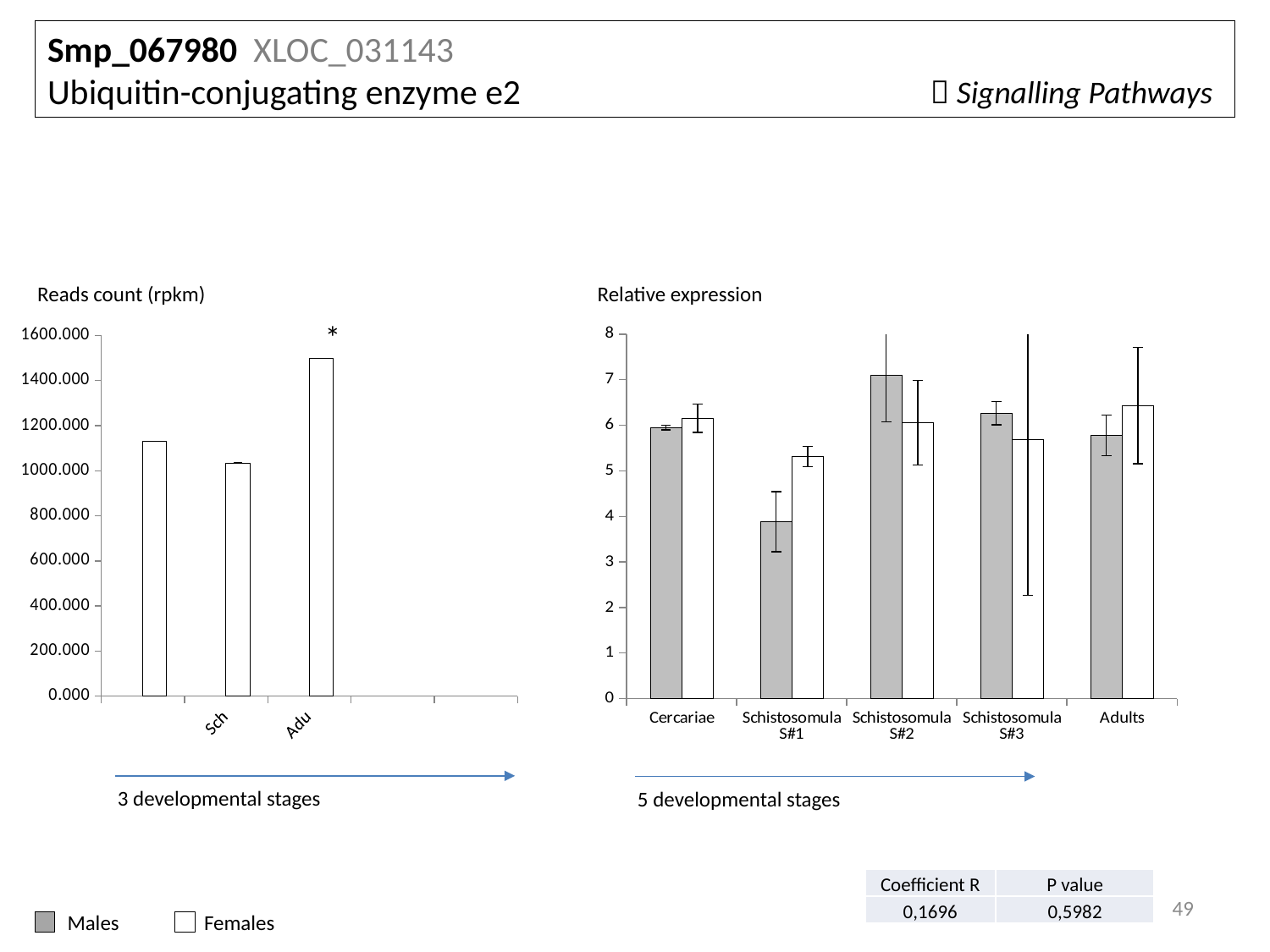

Smp_067980 XLOC_031143
Ubiquitin-conjugating enzyme e2
 Signalling Pathways
Relative expression
Reads count (rpkm)
*
### Chart
| Category | Males | Females |
|---|---|---|
| Cercariae | 1556.53467499386 | 1130.33097760857 |
| Schistosomula S#2 | 2678.00495028924 | 1034.22106303328 |
| Adults | 1240.34863173321 | 1497.96288247691 |
### Chart
| Category | Males | Females |
|---|---|---|
| Cercariae | 5.948833739932435 | 6.156112214698428 |
| Schistosomula S#1 | 3.884971076366685 | 5.315046494536003 |
| Schistosomula S#2 | 7.101725471916705 | 6.055751926230375 |
| Schistosomula S#3 | 6.264827348857647 | 5.6801576270719405 |
| Adults | 5.780922521361113 | 6.429245322647764 |3 developmental stages
5 developmental stages
| Coefficient R | P value |
| --- | --- |
| 0,1696 | 0,5982 |
49
Males 	 Females

## Slide 50
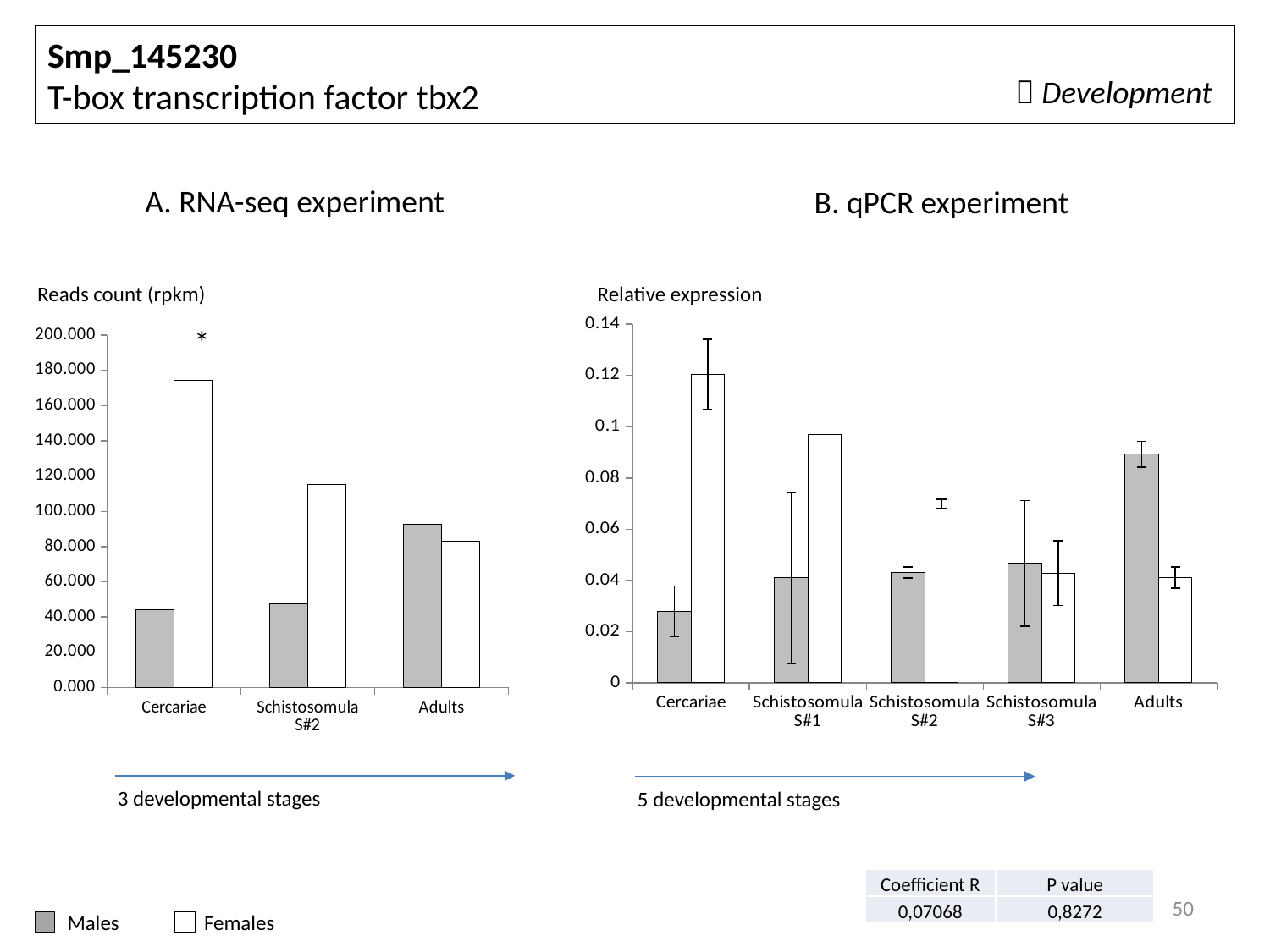

Smp_145230
T-box transcription factor tbx2
 Development
A. RNA-seq experiment
B. qPCR experiment
Relative expression
Reads count (rpkm)
### Chart
| Category | Males | Females |
|---|---|---|
| Cercariae | 0.028011143606320267 | 0.12049156798670146 |
| Schistosomula S#1 | 0.041049328386811884 | 0.09697735829898446 |
| Schistosomula S#2 | 0.043108248992451595 | 0.0698959459230477 |
| Schistosomula S#3 | 0.04666910736759705 | 0.042831979094928876 |
| Adults | 0.08926816815479163 | 0.04111037866028948 |*
### Chart
| Category | Males | Females |
|---|---|---|
| Cercariae | 44.16337248083001 | 174.346259038076 |
| Schistosomula S#2 | 47.27880138297779 | 115.452258859622 |
| Adults | 92.5357611760784 | 82.90380029165 |3 developmental stages
5 developmental stages
| Coefficient R | P value |
| --- | --- |
| 0,07068 | 0,8272 |
50
Males 	 Females

## Slide 51
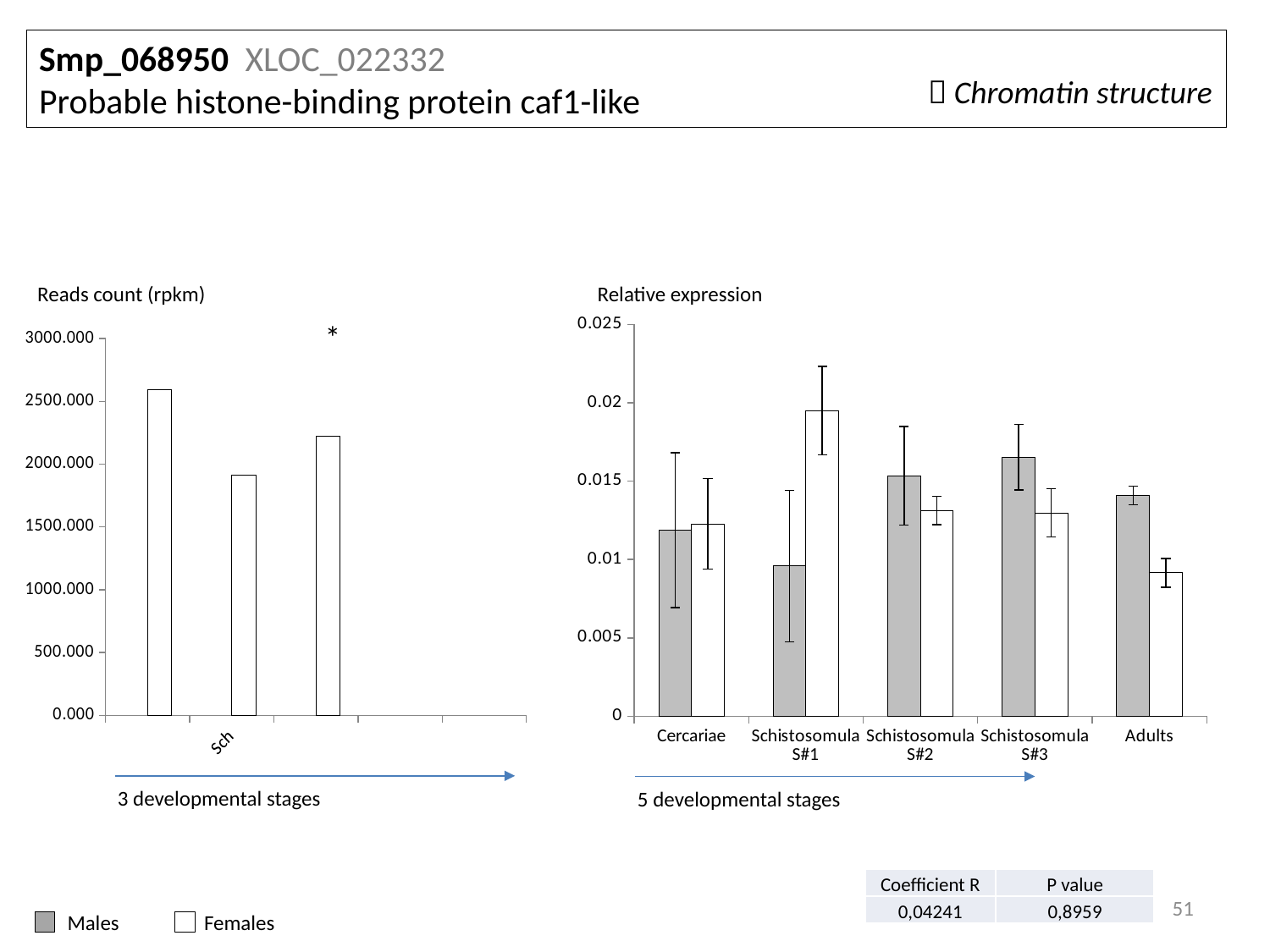

Smp_068950 XLOC_022332
Probable histone-binding protein caf1-like
 Chromatin structure
Relative expression
Reads count (rpkm)
*
### Chart
| Category | Males | Females |
|---|---|---|
| Cercariae | 0.011877020077084707 | 0.012271537411189401 |
| Schistosomula S#1 | 0.009587529379162091 | 0.019492426519094642 |
| Schistosomula S#2 | 0.015337239569843786 | 0.01312396460507143 |
| Schistosomula S#3 | 0.016518821327099965 | 0.012972394791564697 |
| Adults | 0.014089332143128866 | 0.009149409464578567 |
### Chart
| Category | Males | Females |
|---|---|---|
| Cercariae | 3850.90733180071 | 2591.84082489843 |
| Schistosomula S#2 | 5890.50545203415 | 1910.85459931619 |
| Adults | 3606.34604156558 | 2222.33969273036 |3 developmental stages
5 developmental stages
| Coefficient R | P value |
| --- | --- |
| 0,04241 | 0,8959 |
51
Males 	 Females

## Slide 52
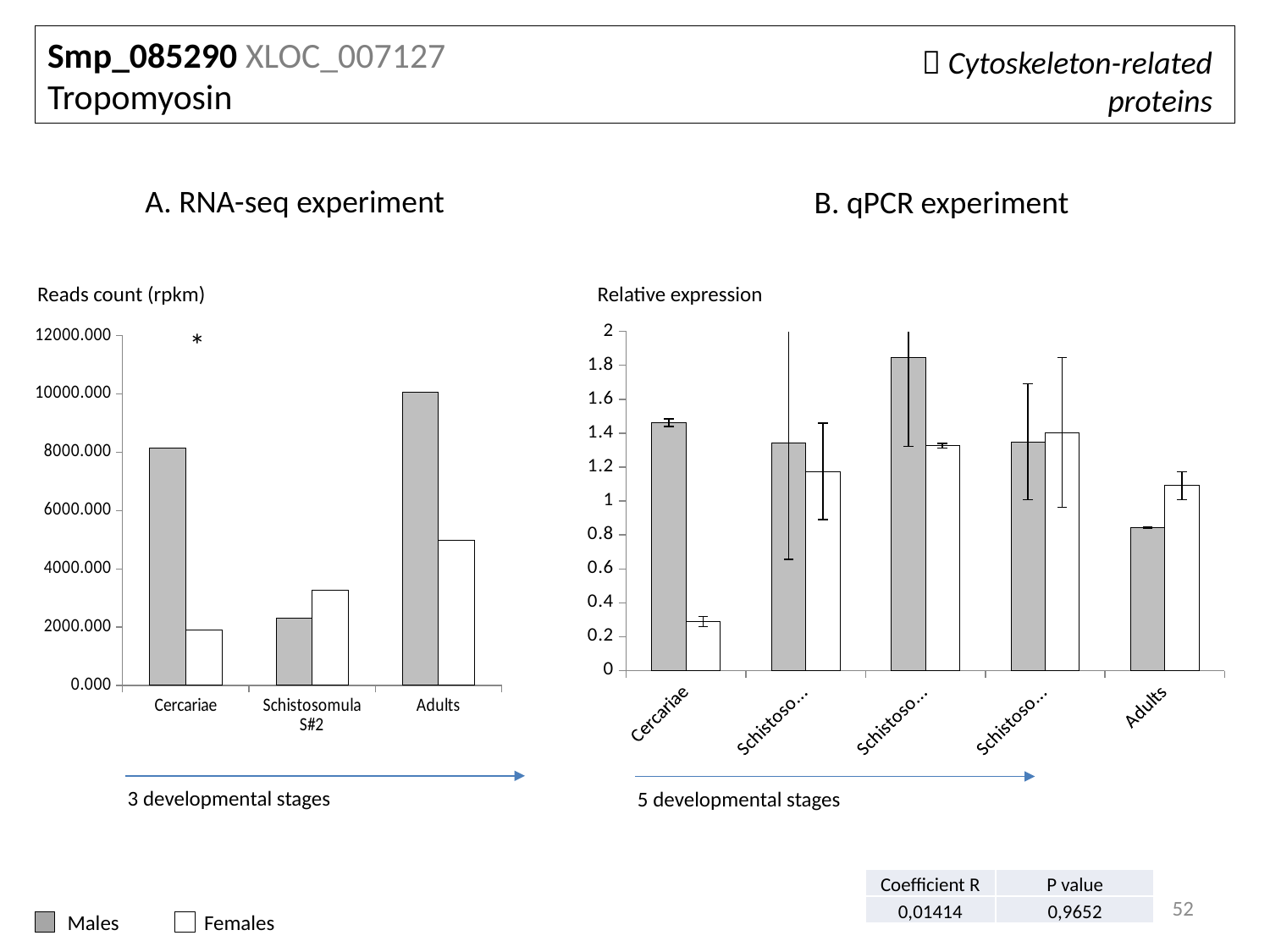

Smp_085290 XLOC_007127
Tropomyosin
 Cytoskeleton-related proteins
A. RNA-seq experiment
B. qPCR experiment
### Chart
| Category | Males | Females |
|---|---|---|
| Cercariae | 1.4617977919060832 | 0.28972581462083513 |
| Schistosomula S#1 | 1.3407023919457262 | 1.1745160477904868 |
| Schistosomula S#2 | 1.8441386703343523 | 1.324944167309783 |
| Schistosomula S#3 | 1.3486060549181236 | 1.403458398779887 |
| Adults | 0.8435240628354337 | 1.0903683218130646 |Relative expression
Reads count (rpkm)
*
### Chart
| Category | Males | Females |
|---|---|---|
| Cercariae | 8129.7422625138215 | 1887.3441363113764 |
| Schistosomula S#2 | 2302.03366099924 | 3261.7584885677743 |
| Adults | 10050.331993134489 | 4983.58577766939 |3 developmental stages
5 developmental stages
| Coefficient R | P value |
| --- | --- |
| 0,01414 | 0,9652 |
52
Males 	 Females

## Slide 53
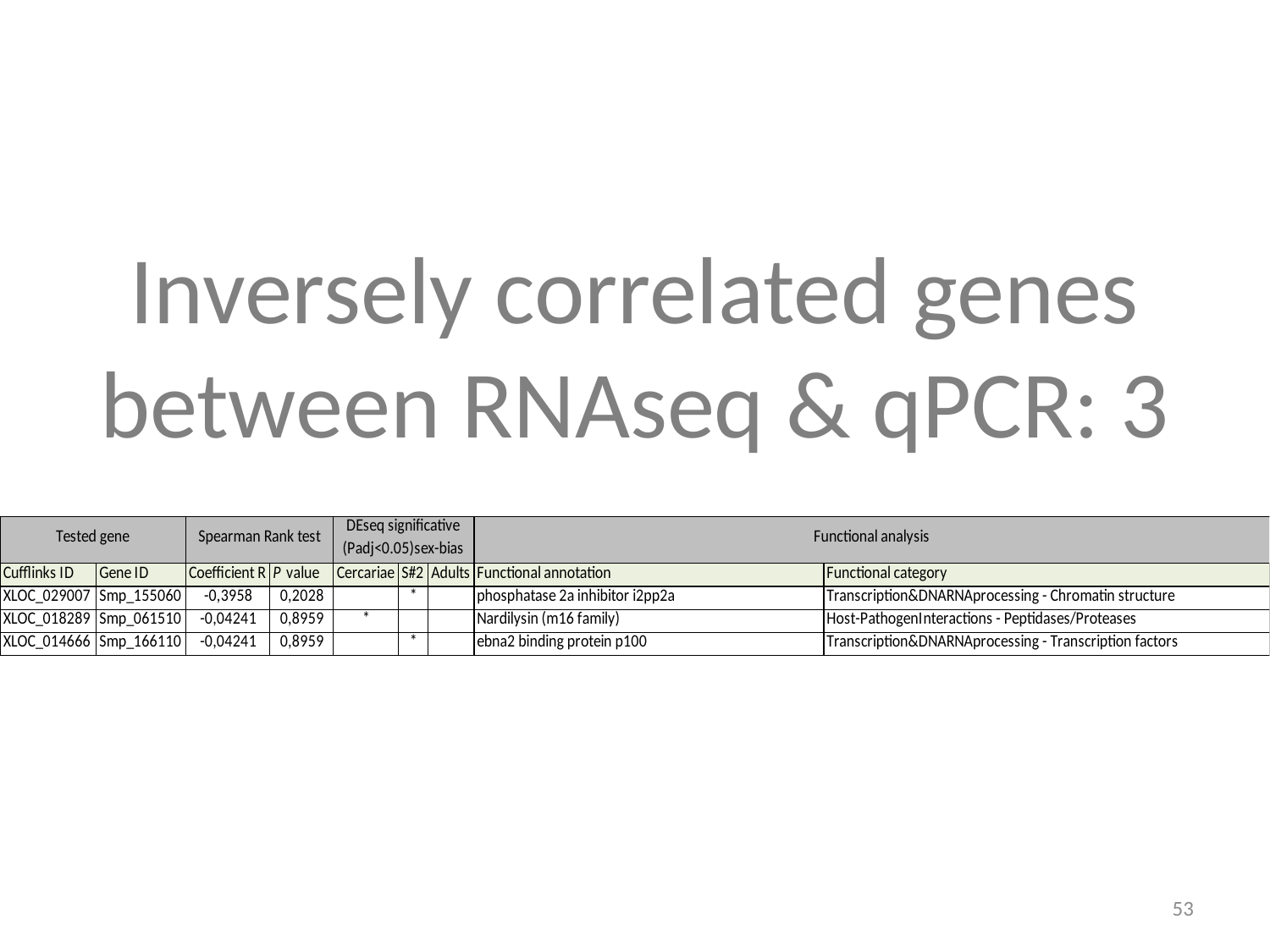

Inversely correlated genes between RNAseq & qPCR: 3
53

## Slide 54
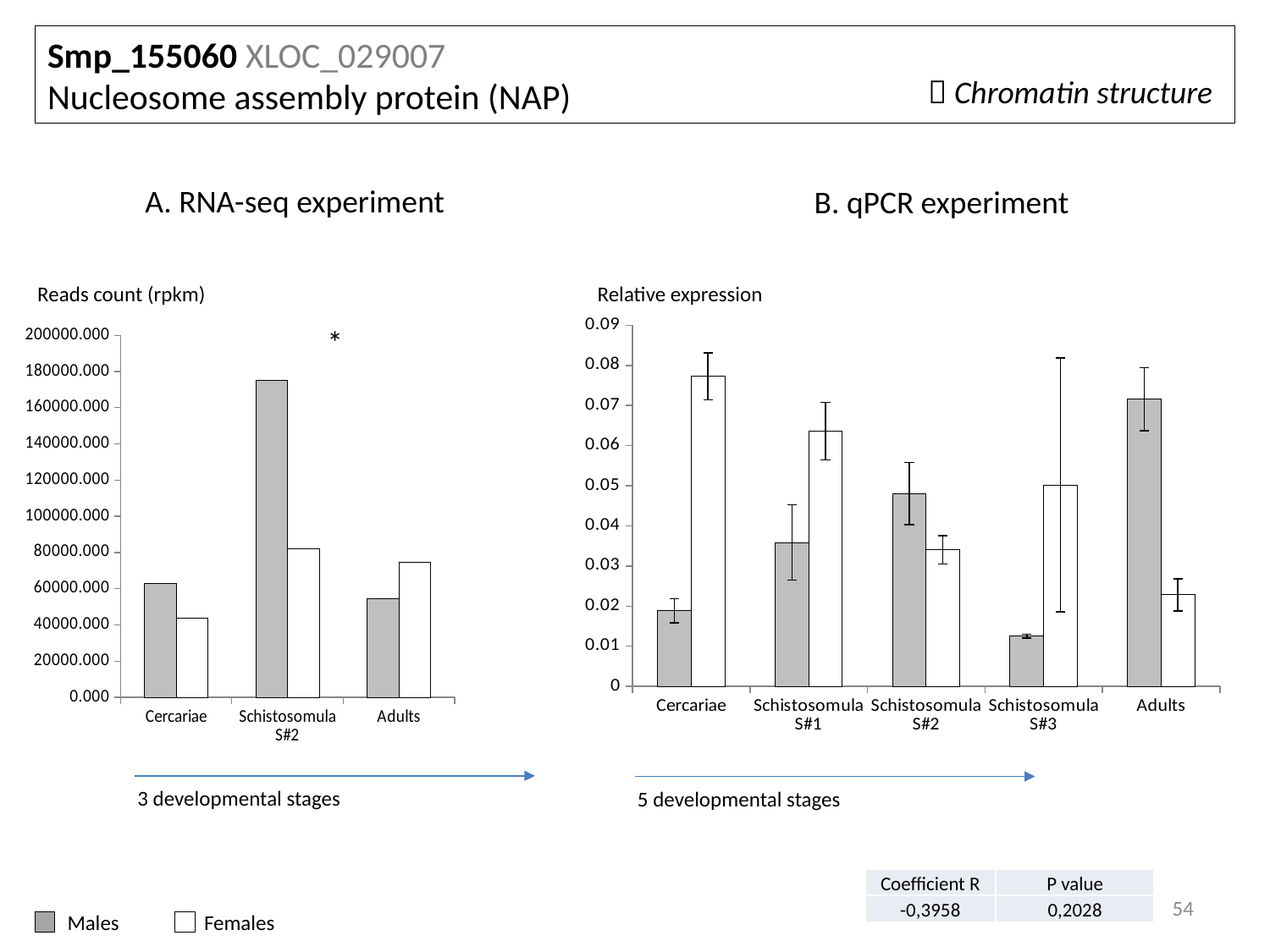

Smp_155060 XLOC_029007
Nucleosome assembly protein (NAP)
 Chromatin structure
A. RNA-seq experiment
B. qPCR experiment
Relative expression
Reads count (rpkm)
*
### Chart
| Category | Males | Females |
|---|---|---|
| Cercariae | 0.018820080231943585 | 0.0773102663277394 |
| Schistosomula S#1 | 0.03584904046656495 | 0.06361280649087236 |
| Schistosomula S#2 | 0.0480601639247265 | 0.03404998654172504 |
| Schistosomula S#3 | 0.012535625247105622 | 0.05019370781807669 |
| Adults | 0.07157630941056803 | 0.022846394280057122 |
### Chart
| Category | Males | Females |
|---|---|---|
| Cercariae | 62936.1289020496 | 43872.36023102517 |
| Schistosomula S#2 | 174900.852904689 | 81850.15741638906 |
| Adults | 54349.8635267364 | 74331.83086357276 |3 developmental stages
5 developmental stages
| Coefficient R | P value |
| --- | --- |
| -0,3958 | 0,2028 |
54
Males 	 Females

## Slide 55
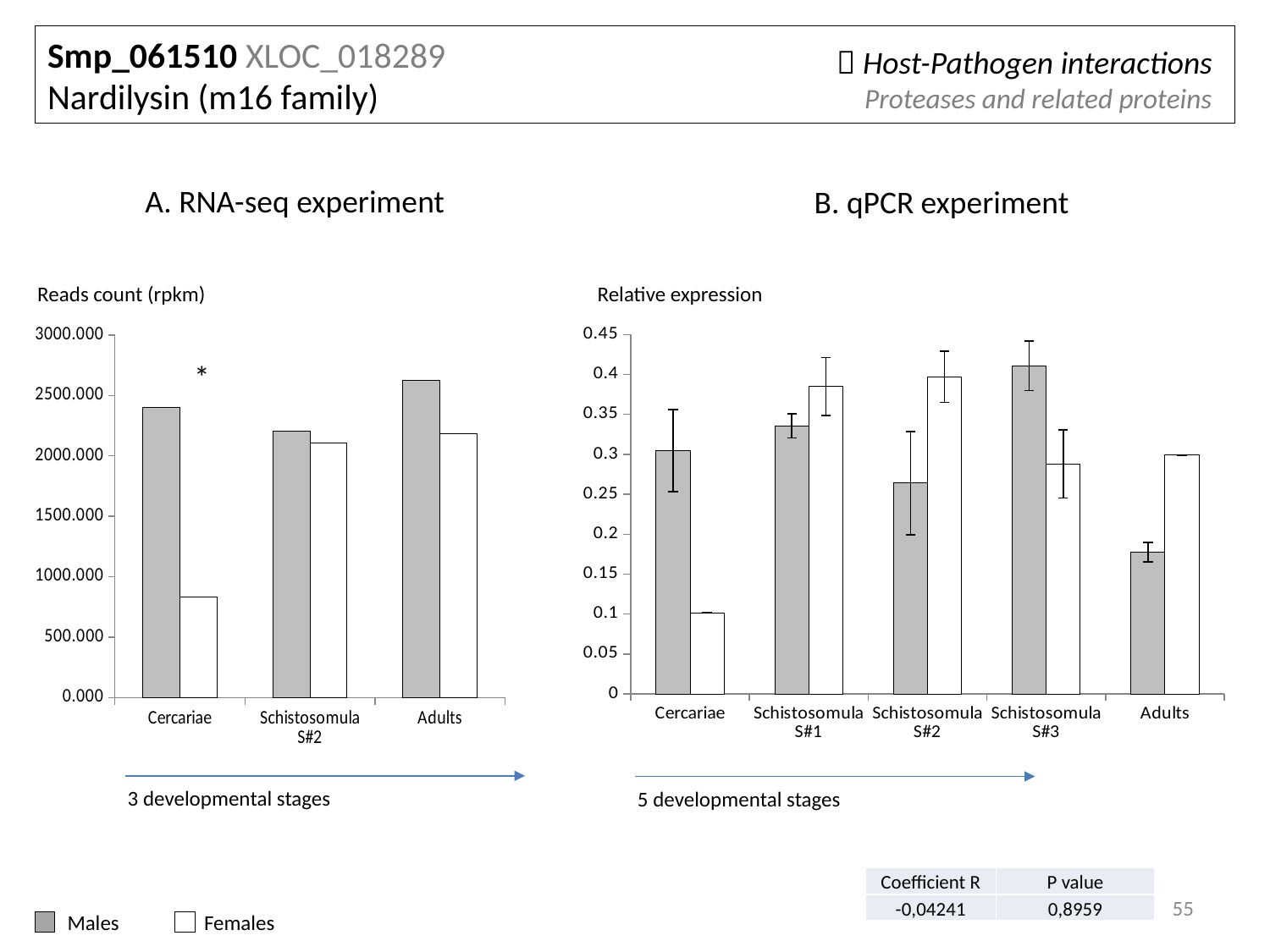

Smp_061510 XLOC_018289
Nardilysin (m16 family)
 Host-Pathogen interactions
Proteases and related proteins
A. RNA-seq experiment
B. qPCR experiment
Relative expression
Reads count (rpkm)
### Chart
| Category | Males | Females |
|---|---|---|
| Cercariae | 2404.08833117678 | 828.2670408447251 |
| Schistosomula S#2 | 2204.76195734765 | 2106.485952416512 |
| Adults | 2622.900375902 | 2181.621762680514 |
### Chart
| Category | Males | Females |
|---|---|---|
| Cercariae | 0.30478638157333193 | 0.10172005774147361 |
| Schistosomula S#1 | 0.3358010973000273 | 0.3849843879436314 |
| Schistosomula S#2 | 0.2639358395976089 | 0.3972889642819326 |
| Schistosomula S#3 | 0.41113313272409424 | 0.2881390265605966 |
| Adults | 0.177428493100136 | 0.29898567797609366 |*
3 developmental stages
5 developmental stages
| Coefficient R | P value |
| --- | --- |
| -0,04241 | 0,8959 |
55
Males 	 Females

## Slide 56
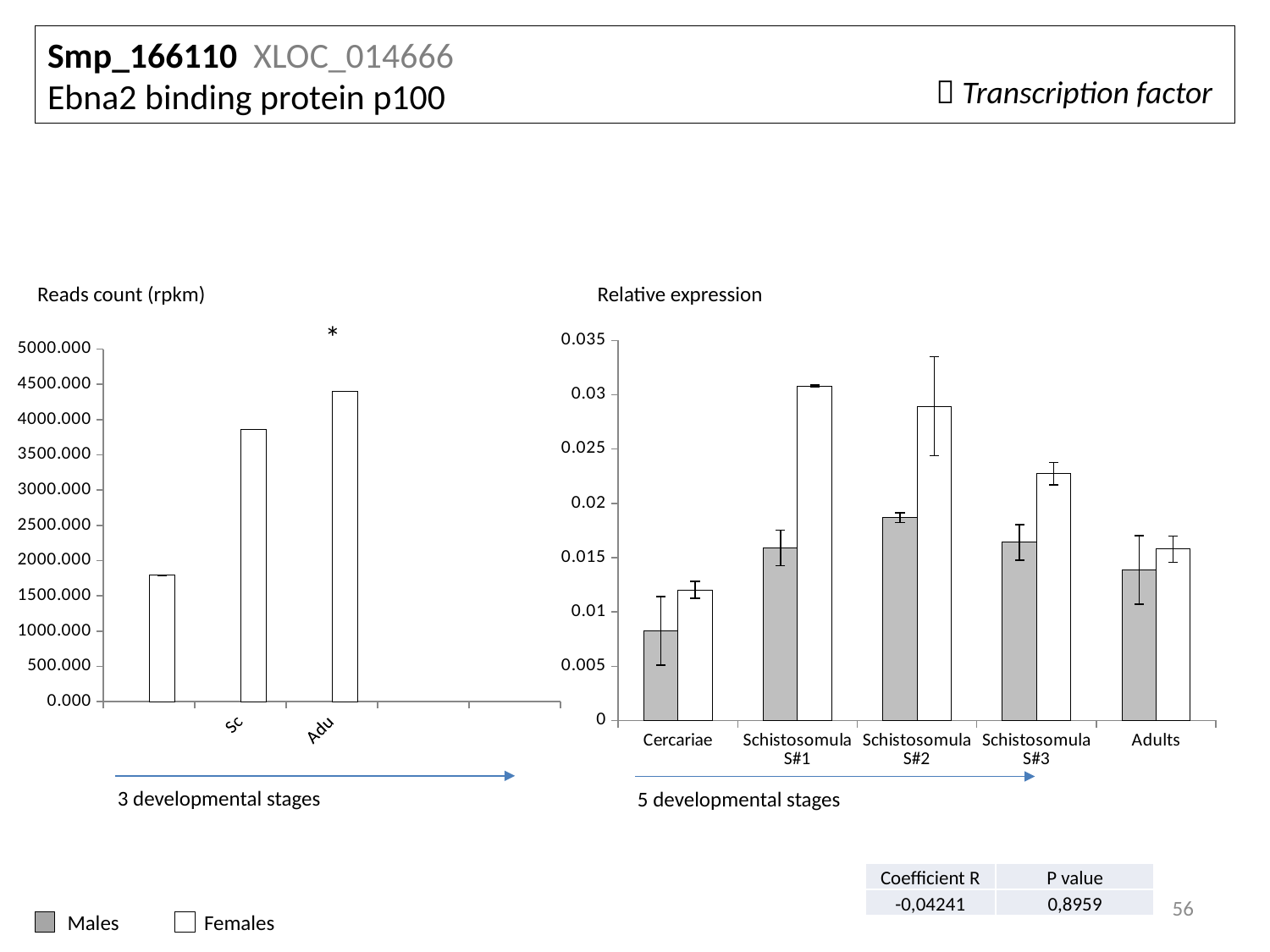

Smp_166110 XLOC_014666
Ebna2 binding protein p100
 Transcription factor
Relative expression
Reads count (rpkm)
*
### Chart
| Category | Males | Females |
|---|---|---|
| Cercariae | 0.008251543146315501 | 0.012033258535937792 |
| Schistosomula S#1 | 0.015890239821418427 | 0.030795045912032193 |
| Schistosomula S#2 | 0.018687477807759964 | 0.02894125484087219 |
| Schistosomula S#3 | 0.016410161348201613 | 0.022723208488900394 |
| Adults | 0.013870631784130432 | 0.0157867144222601 |
### Chart
| Category | Males | Females |
|---|---|---|
| Cercariae | 4919.03813702014 | 1791.69182388597 |
| Schistosomula S#2 | 11021.669708179 | 3860.39250871675 |
| Adults | 5524.54444523767 | 4401.74547396173 |3 developmental stages
5 developmental stages
| Coefficient R | P value |
| --- | --- |
| -0,04241 | 0,8959 |
56
Males 	 Females
